# Tendon transfer to unossified bone in a porcine model: potential implications for early tibialis anterior tendon transfers in children with clubfeet

**DOI:** 10.1007/s11832-016-0799-4

**Published:** 2016-11-30

**Authors:** Matthew A. Halanski, Sam Abrams, Rachel Lenhart, Ellen Leiferman, Teresa Kaiser, Emily Pierce, Rachel Rebekah Franklin, Dayton Opel, Kenneth J. Noonan, Thomas D. Crenshaw

**Affiliations:** 1 American Family Children’s Hospital, University of Wisconsin, Madison, WI USA; 2 Department of Animal Science, University of Wisconsin, Madison, USA

**Keywords:** Clubfoot, Tibialis anterior tendon transfer, Unossified, Apophysis, Porcine model, Tendon transfer

## Abstract

**Purpose:**

Tibialis anterior tendon transfers (TATT) are commonly performed in young children following Ponseti casting for clubfeet. The classic TATT involves advancing the tendon through a hole drilled in the ossified cuneiform. The aim of this study was to determine if tendons transferred through unossified bones have untoward effects on subsequent bone development.

**Method:**

Twenty-five piglets underwent one of five surgical procedures. An 18-gauge needle was then used to place a tunnel through the bony or cartilaginous portion of the calcaneus (through direct visualization) and isolated slips of the *flexor digitorum superficialis* (FDS) were placed through the tunnels, as determined by surgical procedure. Radiographic and/or histologic evaluations of the calcaneal apophyses were then performed. A discrete (1–4) and dichotomous “Normal” or “Abnormal” scoring system was developed and its reliability assessed to grade the appearance of the calcanei. Calcaneal appearances following the surgical procedures were then compared with controls. The average load to failure of a subset of transferred tendons was then compared using an MTS machine.

**Results:**

The proposed apophyseal grading system (1–4) demonstrated an intraclass correlational coefficient (ICC) for consistency of 0.92 [95% confidence interval (CI) 0.88 < ICC < 0.95] and ICC for agreement of 0.91 (95% CI 0.86 < ICC < 0.95), indicating strong agreement and consistency. Similarly, Fleiss’ kappa for the 1–4 scoring system was found to be 0.67, indicating substantial agreement between reviewers. When the 1–4 system was translated into the dichotomous scheme “Normal” and “Abnormal”, the kappa value increased to 0.94, indicating strong agreement. Forty-six apophyses (13 control and 33 operative) were assessed using this scoring scheme. Apophyseal transfers were significantly more abnormal than controls (*p* < 0.0001), while no difference in abnormalities was found following tunnel placement alone (*p* = 1). Mechanical testing of the tendons transferred to bone or through the cartilaginous apophysis demonstrated no significant differences (*p* = 0.2).

**Conclusion:**

Tendon transfers through unossified bones altered subsequent bone development.

**Significance:**

While the long-term consequence of these structural changes is unknown, these findings suggest that tendon transfers through unossified bones should be avoided and alternative methods of tendon fixation explored.

**Electronic supplementary material:**

The online version of this article (doi:10.1007/s11832-016-0799-4) contains supplementary material, which is available to authorized users.

## Background

The Ponseti method has revolutionized the treatment of clubfoot deformity [1–3]. Despite its success in obtaining deformity correction, up to 40% of children treated in Ponseti’s own hands went on to need a tibialis anterior tendon transfer (TATT) [4, 5]. Current teaching suggests that TATT should be delayed until the ossific nucleus of the accepting cuneiform reaches a sufficient size on radiographs to accept the tendon [6, 7]. Many of these patients have a dynamic supination deformity that is treated with transfer of the tibialis anterior tendon to the third cuneiform [6–10]. A review of the literature revealed that, while this delay is adhered to clinical practice, there have been no studies performed on humans or animals to test this assertion. The objectives of this study were to discern if tendon transfer to “pre-ossified” bone had: (1) any effect on subsequent ossification and (2) if such transfers differed in pullout strength when compared to tendon transfers to ossified bone. Our null hypotheses were that transfers to pre-ossified bone would have no effect on subsequent ossification and that such transfers would demonstrate no significant difference in load to failure when compared to the standard osseous transfers.

## Methods

### Choice of model

**Fig. 1:**
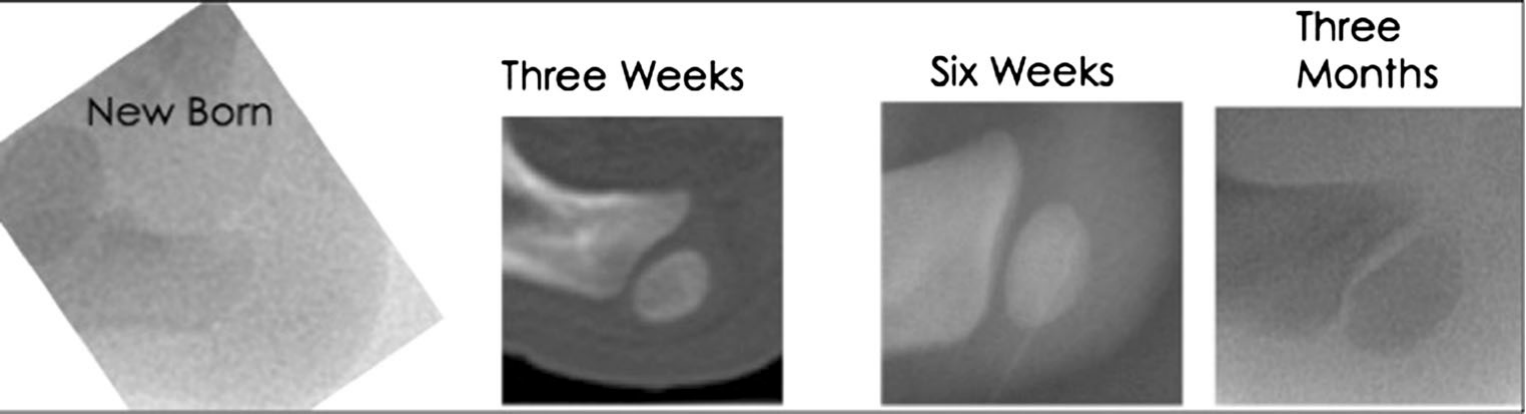
Radiographic appearance of porcine calcaneal apophysis at different ages

After evaluating the rat, rabbit, and piglet, the newborn piglet calcaneal apophysis was chosen as the model for this project. This model provided an easily accessible ossification center that had not yet radiographically begun to ossify at the time of surgery. Baseline samples demonstrated that ossification did occur soon after birth, at ~3 weeks (Fig. 1).

**Fig. 2:**
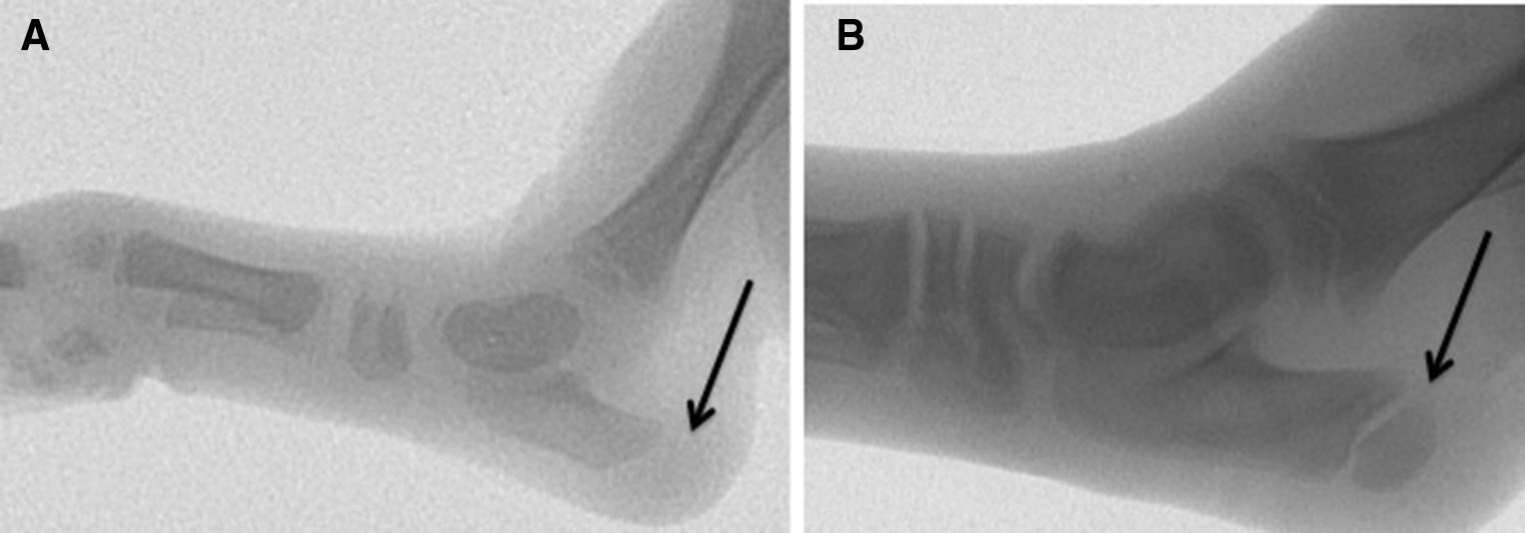
Lateral radiographs of porcine hind limbs. Newborn (**a**) and 9 weeks old (**b**) demonstrate the “pre-ossified” calcaneal apophysis and subsequent ossification (*arrows*)

**Fig. 3:**
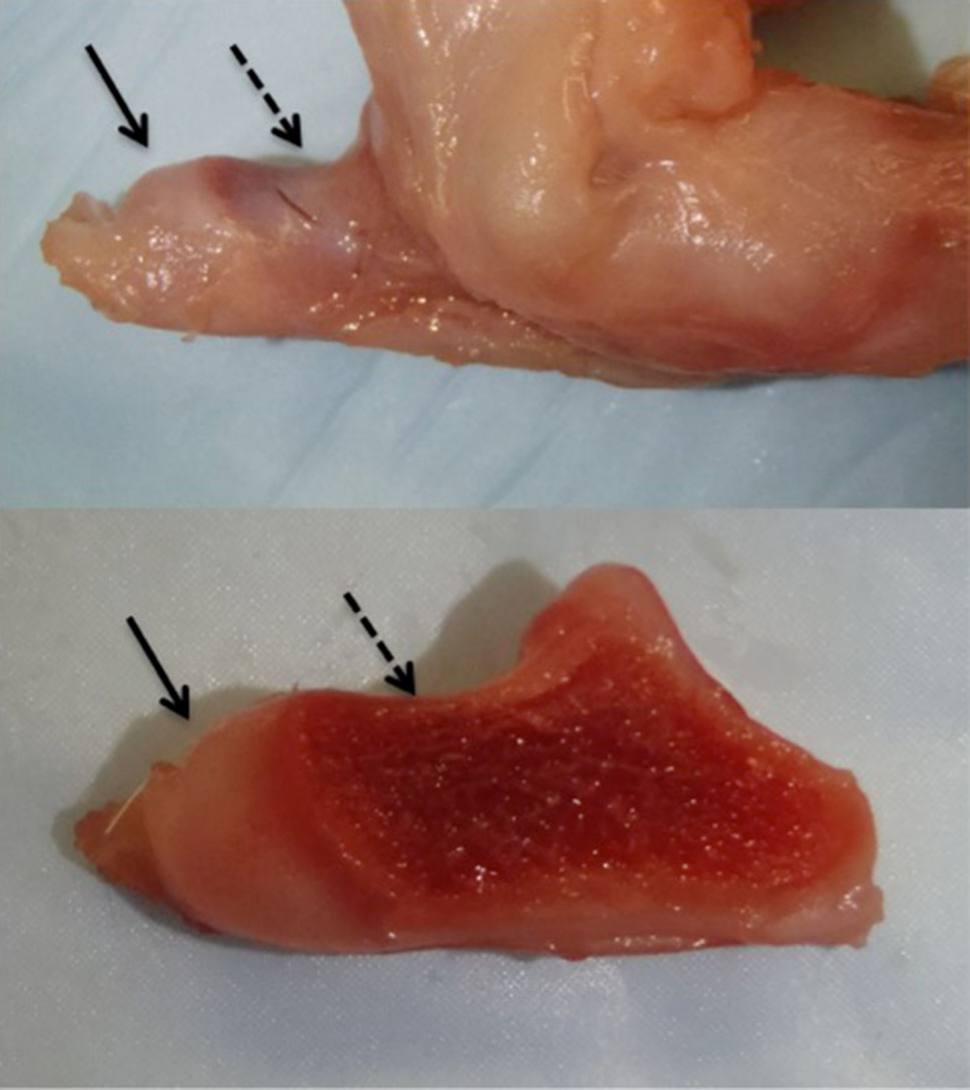
Dissection of a newborn piglet *os calcis* and a transected view. The *solids arrow* point to the cartilaginous portion and the *dotted arrows* indicate the ossified calcaneus

**Fig. 4:**
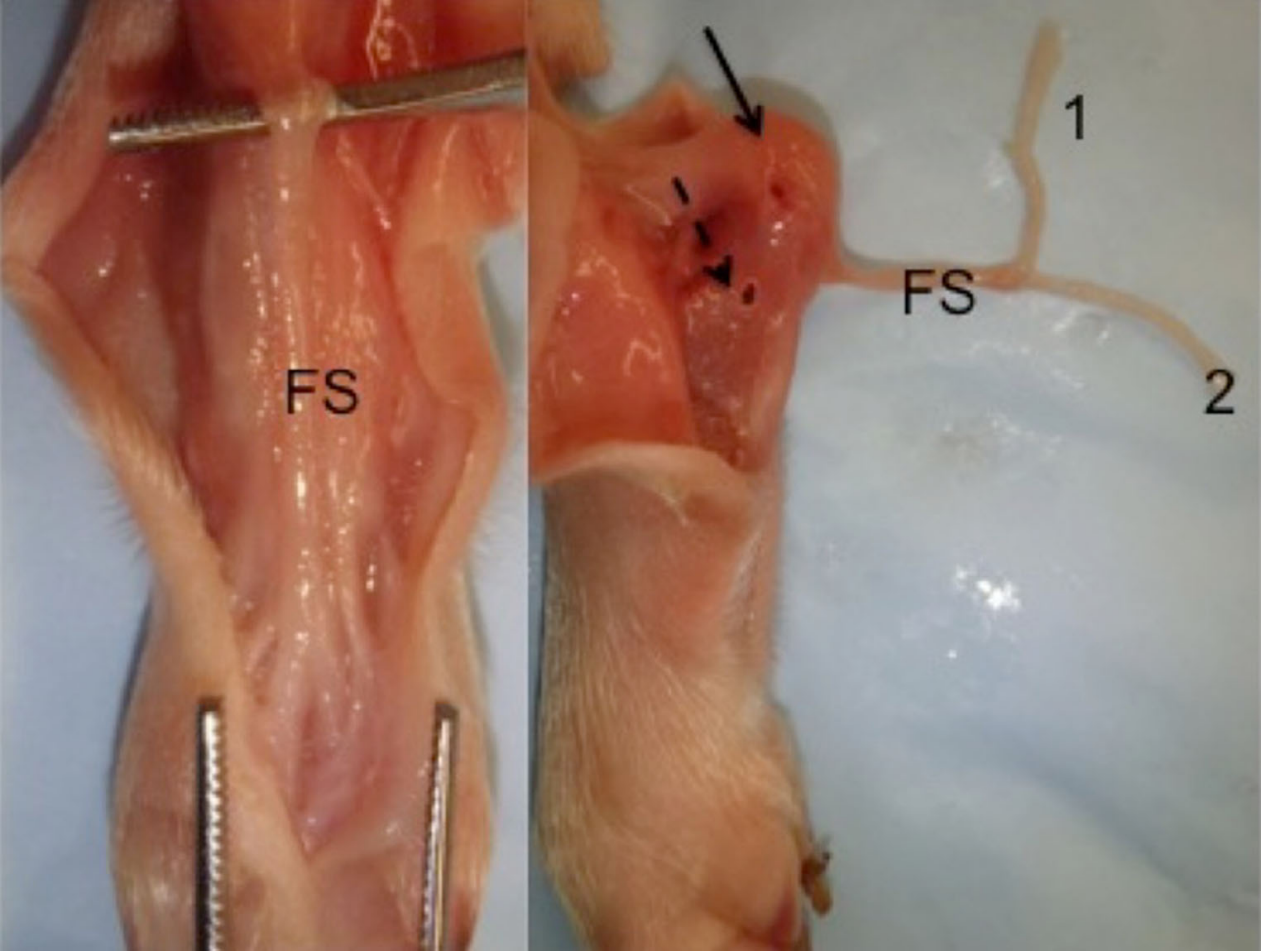
Posterior approach to the hind limb of a cadaveric newborn piglet. The flexor superficialis (*FS*) is marked in the figure on the *left*. The picture on the right demonstrates the holes in the ossified (*dotted arrow*) or cartilaginous (*solid arrow*) calcaneus. Numbers *1* and *2* demonstrate the two slips of the tendon to be transferred in procedure 4

In all procedures described, tendon transfers were performed on the calcanei of newborn (<48 h of age) pigs (Figs. 2 and 3). Piglets were anesthetized, placed in a prone position, and the lower extremities prepped and draped for surgery. For all procedures, a midline posterior incision was performed extending proximal to the *os calcis* distal towards the midfoot. Posterior dissection was carried down until the *flexor digitorum superficialis* (FDS) was identified crossing superficial to the tendo-Achilles. The FDS was freed from the underlying tendo-Achilles, then followed and dissected free from the surrounding soft tissues distally to the level of the metatarsals (Fig. 4). The individual tendon slips (*N* = 4 each going to a separate digit) were then transected. Single tendon slips were then transferred to either the unossified calcaneal apophysis and/or through the ossified calcaneal body, as indicated by the specific procedure (dual procedure had a single slip transferred through each location). All tunnels were placed under direct vision (Figs. 2 and 3), without radiographic imaging. Following all surgeries, piglets were recovered and returned to their sow, without any weight-bearing restrictions or immobilization.

### Procedures

#### Dual transfer

**Fig. 5:**
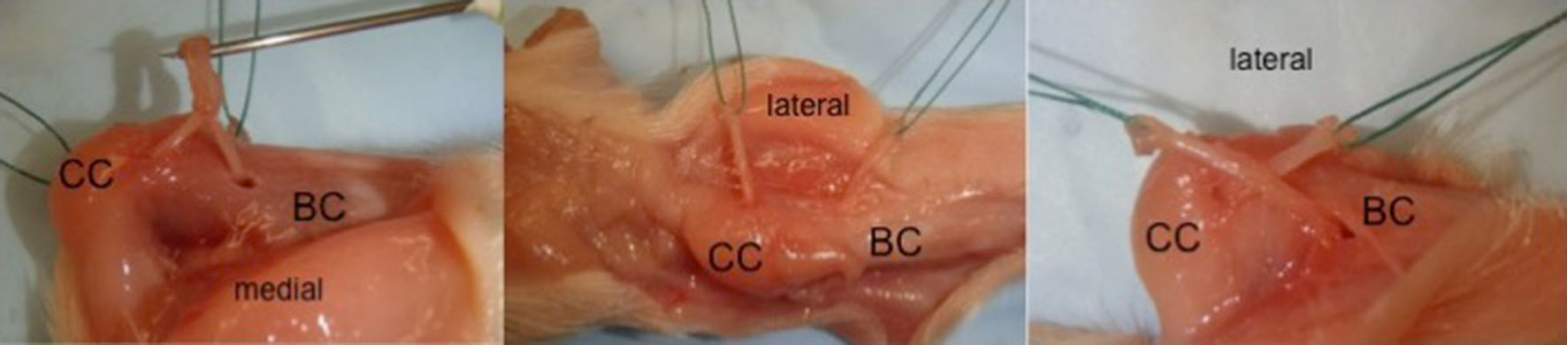
Dual transfer procedure. The tendon slips are pulled through from medial to lateral (*left* and *center*). The tendons are then sutured to one another on the lateral side of the calcaneus. *BC* bony calcaneus, *CC* cartilaginous calcaneus

The FDS tendon was harvested as described above. The individual tendon slips were visually inspected and two slips similar in size were chosen for this procedure. One slip was utilized for a transfer through the cartilaginous apophysis and the other for transfer through the osseous calcaneal body. An 18-gauge needle was then used to create two tunnels, one through the cartilaginous apophysis and the other through the ossified body of the calcaneus. One tendon slip was then passed through each tunnel (medial to lateral), and the two tendon slips were sutured to one another on the lateral side of the calcaneus (Fig. 5).

This method was initially chosen as it would eliminate the need for any internal fixation, help ensure equal tension was being applied to each tendon slip, allow for a single limb to be used in mechanical testing (each slip could be used to test bony healing and cartilaginous healing), and allow for the non-operative limb to serve as an anatomic control (to evaluate apophyseal ossification). This dual transfer was performed on the right calcaneus of six piglets, while the left limb served as a non-operative control. Pigs were recovered and sacrificed 6 weeks after the index procedure. The limbs were harvested; however, the robust inflammatory and healing response made identification of the isolated tendon slips for mechanical testing difficult. Therefore, the decision was made to evaluate the calcanei by Faxitron and histology. These studies demonstrated irregularities in the apophyses following these tendon transfers. Using this information, the investigators were forced to develop alternative procedures to assess the effects of different aspects of the procedure (tunnel placement, free tendon placement, intact tendon placement) on apophyseal development, while trying to assess differences in pullout strength between bony and cartilaginous transfers.

#### Empty tunnel and free tendon segment transfers

**Fig. 6:**
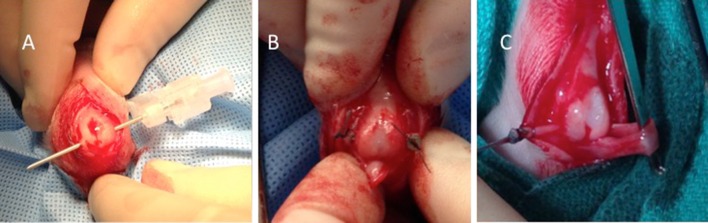
Three of the other procedures performed. **a** Empty tunnel/coring: needle passed through the apophysis. **b** Free FDS tendon segment passed through tunnel held in place with buttons. **c** Single tendon passed through either apophysis (shown) or ossified calcaneus

To evaluate whether changes noted in the apophyses following our initial dual procedures were due to the trauma of the tunnel placed through the apophysis or due to the presence of the tendon in the apophyseal tunnel, five piglets underwent placement of an empty tunnel through the cartilaginous calcaneal apophysis using the 18-gauge needle on one limb (Fig. 6a), while the contralateral limb had a free FDS segment placed through the tunnel that was held in place medially and laterally by custom-made stainless steel buttons (Fig. 6b). These piglets were recovered and harvested at 6 weeks and the limbs evaluated using Faxitron radiographs.

#### Single tendon transfers: to cartilaginous apophysis or to ossified calcaneus

Taking into account the difficulty in separating the FDS tendon slips from the surrounding soft tissue in the initial dual transfer procedure described above, the authors decided to perform single tendon slip transfers to either the apophysis or the bony calcaneus. In addition, the decision was made to decrease the recovery time to3–4 weeks in hopes of better identifying the transferred tendons from the surrounding inflammatory tissue.

The identical approach and steps described for the dual transfer were performed; however, only a single tunnel (apophyseal or bony) was made and a single slip of FDS transferred through the tunnel. The tendon was held in placed by suturing the free end of the tendon slip to small custom-made stainless steel buttons that prevented it from sliding back into the tunnel (Fig. 6c). No excessive tension was applied to any of the tendon transfers. Four piglets underwent unilateral apophyseal transfer, while four others underwent bony transfer. Three piglets in each cohort also had a biodegradable (polycaprolactone, PCL) sheet wrapped around the proximal FDS tendon in hopes of keeping it from adhering to surrounding tissues; however, this was unsuccessful and the practice abandoned. These eight piglets were sacrificed 3 weeks post-procedure. Six of the eight piglets had their calcanei evaluated by portable radiograph and/or split Faxitron, with the contralateral limb serving as a 3-week non-operative control. Imaging of two pigs, one apophyseal and one bony, was unavailable for final review. Six of the eight (*N* = 4 bone, *N* = 2 apophysis) had the tendon transfers dissected free and samples available for mechanical testing. During the tissue preparation for mechanical testing, the investigators were concerned that some of the apophyseal tendon healing may have been disrupted during dissections while isolating the tendon (healing found to be broad and at the cartilage surface). Therefore, five additional piglets underwent bilateral apophyseal only transfers that were harvested at 4 weeks with minimal soft tissue dissection performed prior to mechanical testing.

### Radiography

After collection, the calcanei were evaluated either with high-resolution Faxitron imaging (samples were either whole or split longitudinally) or standard portable digital radiography

### Histology

The calcanei were split and grossly evaluated, and then the samples processed for standard histological processing including hematoxylin and eosin staining. These were only used qualitatively.

### Scoring the appearance of the calcanei

**Fig. 7:**
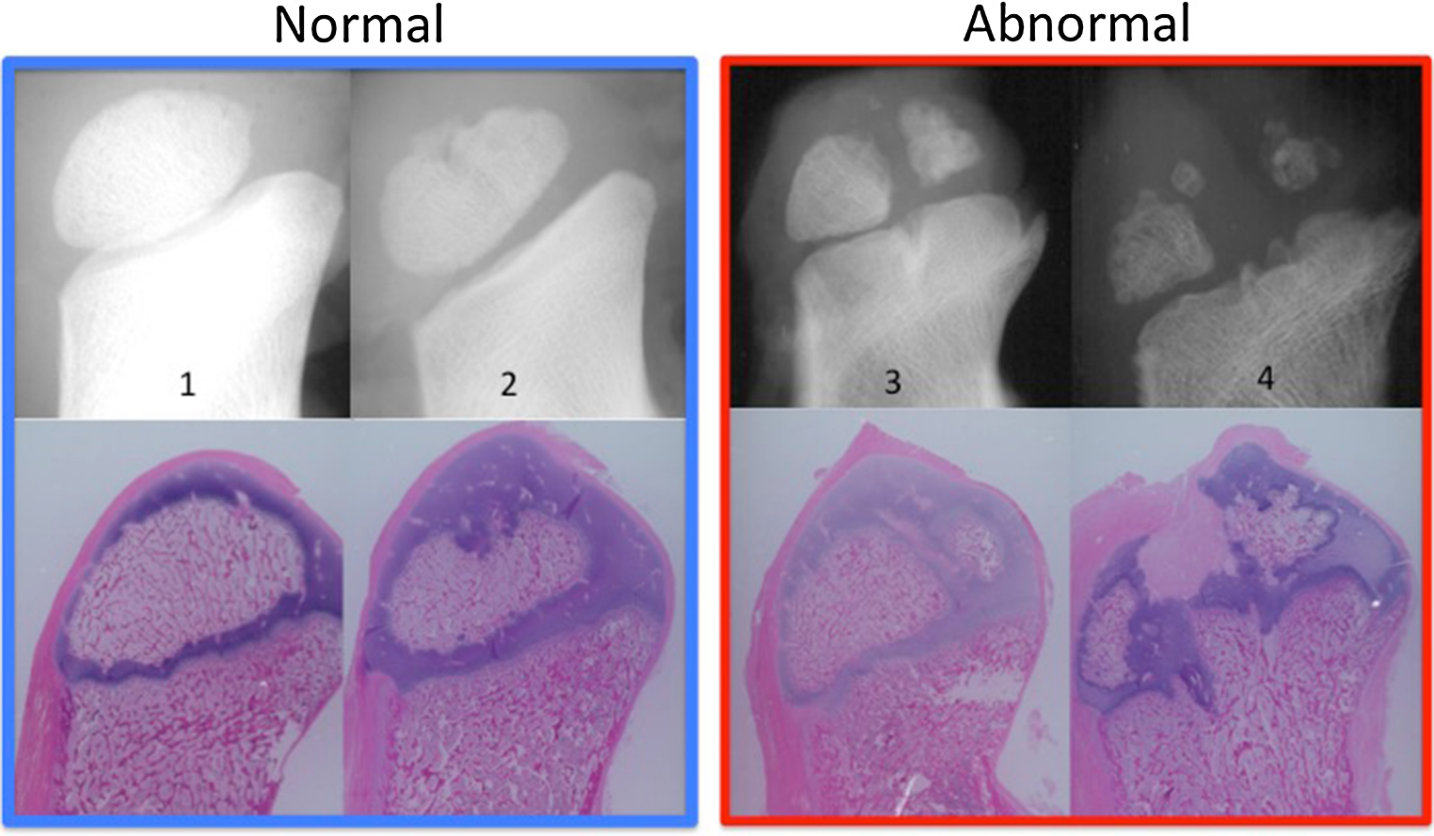
Examples of the radiographic and histological appearance of various apophyses termed “Normal” versus “Abnormal”

Using the radiographic and/or histologic images, a grading system (1–4) was developed. The apophyses were graded as “Normal” [smooth round contour (1) or slightly irregular with small ossicles (2)] or “Abnormal” [split (3) or fragmented (4)] (shown in Fig. 7). The scoring system and a set of images for each sample were then provided to three independent reviewers who scored each of the samples in triplicate (images available in the supplemental material). Fleiss’ kappa was calculated as a measure of agreement between the raters using the (1–4) scale and the dichotomous “Normal” versus “Abnormal” scale. Intraclass correlational coefficients (ICCs) for reader consistency and agreements using the 1–4 scale were also performed. A scheme was then employed in which the aggregated median scores of each individual reader were determined. The aggregated median of the individual medians was then taken as the final (1–4) scoring for the sample. This scheme preserved the discrete nature of the (1–4) scoring system. After a final (1–4) score was given to the sample, scores were then converted to the dichotomous “Normal” or “Abnormal”. Based on these dichotomous scores, a Fisher’s exact test was then used to compare the effects of the procedures on calcaneal appearance.

### Mechanical testing

**Fig. 8:**
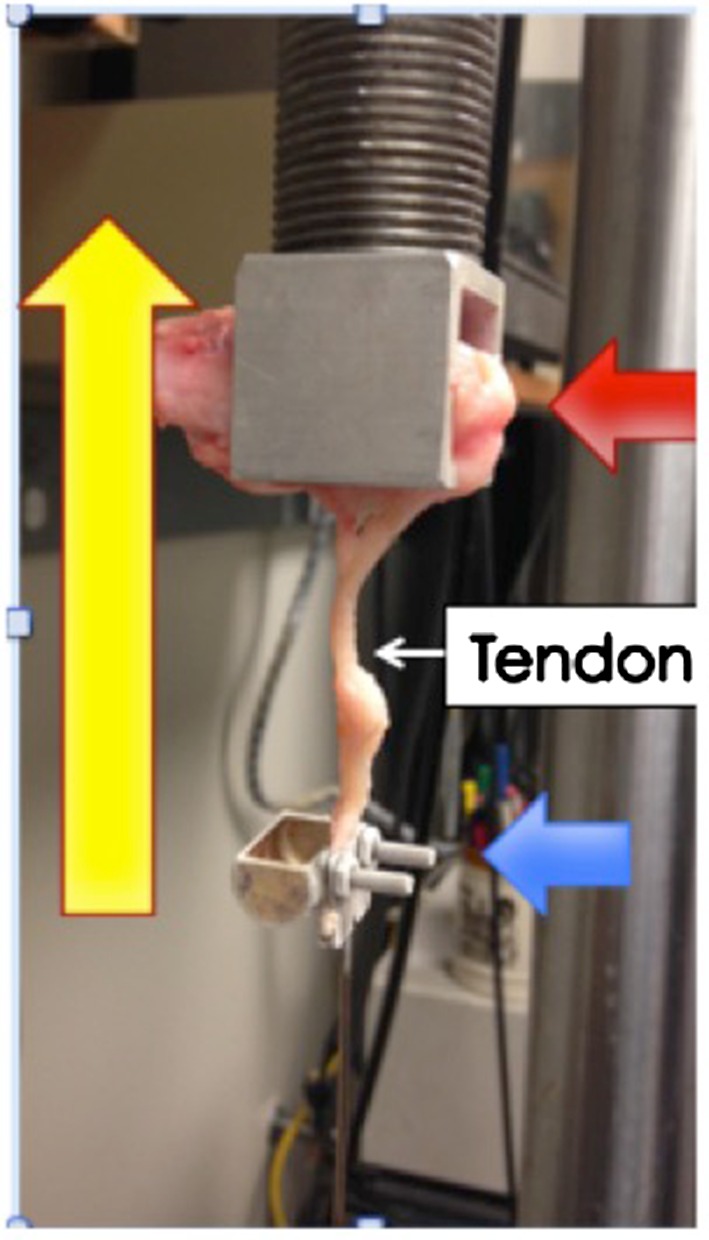
Example of the calcaneus in the MTS jig (*red arrow*) and tendon in the freeze grips (*blue arrow*). The *yellow arrow* denotes the direction of force

The transferred tendon in 16 of the harvested samples were able to be dissected free and capable of being used for mechanical testing. The calcanei of these samples were mounted on a jig in an MTS machine. The custom jig was developed with freeze grips to hold the tendon to prevent slippage (Fig. 8). Load versus displacement curves were obtained and the maximum load to failure recorded. Comparisons between bone transfers (*N* = 4), apophyseal transfers with (*N* = 2), and without (*N* = 10) extensive soft tissue dissection were performed. An unpaired Student’s *t*-test was utilized to compare the average maximum loads between tendons transferred to bone or to tendon.

Table 1 lists all the animals, procedures, ages at sacrifice, and evaluations performed in this study.

**Table 1:** List of piglets, procedure, age at time of procedure and harvest, and evaluation method

Pig	Limb	Surgical procedure	Age at surgery (days)	Days post surgery	Evaluation method	Mechanical testing	PCL membrane
1	R	Dual transfer	2	45	HE/Faxitron		
	L	Non-operative control			HE		
2	R	Dual transfer	2	45	Faxitron		
	L	Non-operative control			Faxitron		
3	R	Dual transfer	2	45	HE		
	L	Non-operative control			HE		
4	R	Dual transfer	2	44	HE and Faxitron		
	L	Non-operative control			HE		
5	R	Dual transfer	2	44	Faxitron		
	L	Non-operative control			Faxitron		
6	R	Dual transfer	2	44	HE/Faxitron		
	L	Non-operative control			HE		
7	R	Empty tunnel	2	23	Faxitron		
	L	Non-operative control			Faxitron		
8	L	Empty tunnel	0	41	Faxitron		
	R	Empty tunnel			Faxitron		
9	L	Free tendon segment	0	41	Faxitron		
	R	Empty tunnel			Faxitron		
10	L	Free tendon segment	0	41	Faxitron		
	R	Empty tunnel			Faxitron		
11	L	Free tendon segment	0	41	Faxitron		
	R	Empty tunnel			Faxitron		
12	L	Free tendon segment	0	41	Faxitron		
	R	Empty tunnel			Faxitron		
13	R	Bone transfer (single)	2	23	None available for review	Y	
	L	Non-operative control			None available for review		
14	R	Bone transfer (single)	0	21	Faxitron/PX	Y	Y
	L	Non-operative control			PX		
15	R	Bone transfer (single)	0	21	Faxitron/PX	Y	Y
	L	Non-operative control			PX		
16	R	Bone transfer (single)	0	21	PX	Y	Y
	L	Non-operative control			PX		
8	R	Apophyseal transfer (single)	2	23	None available for review		
	L	Non-operative control			None available for review		
10	R	Apophyseal transfer (single)	0	21	Faxitron/PX		Y
	L	Non-operative control			PX		
11	R	Apophyseal transfer (single)	0	21	Faxitron/PX	Y	Y
	L	Non-operative control			PX		
12	R	Apophyseal transfer (single)	0	21	Faxitron	Y	Y
	L	Non-operative control			PX		
16	R	Apophyseal transfer (single)	1	30	Faxitron	Y	
	L	Apophyseal transfer (single)			Faxitron	Y	
17	R	Apophyseal transfer (single)	1	30	Faxitron	Y	
	L	Apophyseal transfer (single)			Faxitron	Y	
18	R	Apophyseal transfer (single)	1	30	Faxitron	Y	
	L	Apophyseal transfer (single)			Faxitron	Y	
19	R	Apophyseal transfer (single)	1	30	Faxitron	Y	
	L	Apophyseal transfer (single)			Faxitron	Y	
20	R	Apophyseal transfer (single)	1	30	Faxitron	Y	
	L	Apophyseal transfer (single)	0		Faxitron	Y	

Piglets are grouped by procedure performed, not necessarily in chronological order: *HE* histology, Faxitron (intact or cut samples (prior to histology), *PX* portable X-ray

## Results

### Radiography and histology

The ICCs for the proposed scoring system (1–4) were 0.92 [95% confidence interval (CI) 0.88 < ICC < 0.95] for consistency and 0.91 (95% CI 0.86 < ICC < 0.95) for agreement. Similarly, the inter-reader reliability defined by Fleiss’ kappa was 0.67, indicating substantial agreement. When the (1–4) rating system was converted into a dichotomous “Normal” versus “Abnormal” scheme (shown in Fig. 7), the kappa value increased to 0.94, indicating strong inter-reader agreement. Using the scheme described above, the median of the triplicate scoring of three independent reviewers was utilized to define each sample as “Normal” or “Abnormal”. The effects that each procedure had on the apophyses can be found in Table 2. Summarizing these data demonstrates that abnormal apophyses developed more often when the tendon was placed through the apophyses (17/23) than in controls (0/13) or when no tendon was present in the apophyses (1/10). All non-operative control apophyses were found to be normal regardless of follow-up (3 or 6 weeks). For statistical comparisons, samples in which PCL was utilized (*N* = 6; 3 osseous transfers and 3 apophyseal transfers) were excluded from the analysis to minimize its potential confounding effects. Despite this exclusion criteria, the small sample size at each time point for each surgical cohort precludes statistical comparison between each group. However, some individual statistical comparisons can be made. All of the apophyseal transfers (10/10) but none of the controls (0/7) were abnormal at the 3–4 weeks follow-up (*p* < 0.0001). Conversely, no significant abnormalities or differences were found between controls (0/6) and empty tunnel samples (1/5) at 6 weeks (*p* = 1). Similarly, grouping and comparing the apophyseal appearance between procedures in which no tendon was present with procedures in which it was present demonstrated significant differences (Table 3).

**Table 2:** Summary of the apophyseal appearance based on procedure and length of follow-up

	Normal	Abnormal	Normal	Abnormal
	3–4 Weeks	3–4 Weeks	6 Weeks	6 Weeks
Control	7	0	6	0
Empty tunnel	1	0	5	1
Bone tendon transfer (single) with PCL membrane	3	0		
*Free FDS segment*			*1*	*3*
*Apophyseal tendon transfer (single) with PCL membrane*	*2*	*1*		
*Apophyseal tendon transfer (single)*	*0*	*10*		
*Dual transfer*			*2*	*4*

*Italics* indicates those procedures in which tendon is present in the apophysis

**Table 3:** Grouped comparisons between procedures in which tendon was present in the apophysis compared with those in which it was not (Irregardless of the length of follow-up or presence of PCL membrane)

	Normal	Abnormal	*p*-Value*
Control	13	0	<0.0001
Needle	6	1	<0.005
Bone	3	0	0.02
*Tendon present*	5	18	

*Italic* indicates those procedures in which tendon is present in the apophysis

* Fisher’s exact test (vs. tendon present)

### Mechanical testing

Sixteen specimens underwent mechanical testing. Four of the tendons were transferred to bone, two were to the apophysis dissected free, while ten apophyseal transfers were tested following minimal dissection. Mechanical testing of the tendons that could be dissected free demonstrated no significant differences (*p* = 0.5) in load to failure between bony (27 ± 11 N) and cartilaginous (18 ± 15 N) transfer. Similarly, the load to failure of the ten additional apophyseal transfers with minimal soft tissue dissection demonstrated no significant difference (*p* = 0.2) when compared to the bony transfers (50 ± 33 N).

## Discussion

TATT has classically been performed for a clinically diagnosed dynamic supination deformity in older children [6–10]. Performing this surgery early is thought to prevent later varus recurrence in the hind foot. While dynamic supination has been the classic indication for this procedure in Ponseti-treated patients, the deforming force of the tibialis anterior likely exists before the child establishes normal gait. Recent studies have also shown that there may be a muscle imbalance between the peroneals laterally and the tibialis anterior medially [11–13]. If patients with an overpowering tibialis anterior could be identified earlier, transferring this tendon might prevent recurrences and balance the foot more normally. However, it has been widely assumed that tendon transfers should only be considered when the ideal point of transfer is a bone that is at least partially ossified [6–7]. As such, it has become common practice to delay surgical treatment. The current literature does not reveal any studies that support this assertion, in either humans or animal models.

This current study demonstrates that early tendon transfer, through unossified bone, does appear to alter normal ossification. The long-term consequences of these changes are unknown, as is the ability of the bone to remodel from these changes over time. This will be the focus of further studies in this model. Interestingly, the load to failure of this transfer did not appear significantly different from the transfer to normal bone and actually trended towards being greater in the unossified transfer. Some of this trend difference may be due to the difficulty in dissecting the distinct tendon insertion site in the unossified transfer due to the robust surface healing in these specimens.

Limitations of this study should be pointed out. The model chosen does not use the tendon nor the bone in question in the human procedure. However, the authors’ preliminary investigation included screening rats and rabbits. These smaller animals have ossified tarsal bones very early in life. Similarly, the majority of tarsal bones in the porcine model were ossified at the time of birth. However, the calcaneal apophysis was unique as it was unossified at birth, its surgical exposure simple, and in close proximity of the flexor tendon used for transfer. Another potential limitation is the relatively rapid ossification of this apophysis, as ossification began by 3 weeks of age instead of the 3 years it typically takes for the middle cuneiform to ossify in humans. While a potential limitation, this rapid maturation will allow for simple future investigations into the long-term effects of these transfers and the effects of alternative techniques. Also, the tendon transfers in these animals were not placed under tension and were not immobilized, as is routine when the procedure is performed in children. As these piglets were only a day old, they needed to be able to be recovered and allowed to nurse in a pen with their mother and littermates during the study period. It was felt that any immobilization and/or any materials used for immobilization might jeopardize the safety of the piglets.

Another limitation is in the study design. Originally, the authors had planned on a well-designed study with internal controls using the dual transfer procedure. This would have allowed comparison of the mechanical properties of bony versus cartilaginous transfers, while, at the same time, compared the radiographic/histologic effects between the operative and non-operative limbs. This would have allowed the use of a minimal number of animals. However, after our initial cohort, we learned the limitations of the dual procedure. While it did not allow mechanical testing, it did provide new data which appeared to show that tendon transferred through unossified bone alters subsequent ossification. The authors then devised procedures to better answer the question, “Is it the trauma or the presence of the tendon that causes the apophyseal changes” and “how does tendon–cartilage healing compare to tendon–bone healing (mechanically)”. This, unfortunately, required more procedures and more animals than initially anticipated. The argument could be made that the data would be cleaner if fewer procedures and more uniform imaging and analysis was performed, and the authors acknowledge this fact. However, this was a preliminary study, using a model and procedures never before described. While the investigators could have eliminated certain cohorts from the analysis or publication, the findings throughout all the samples from 3–6 weeks post-operative cohort are consistent in that the presence of the tendon in the cartilaginous tunnel appears to adversely affect early ossification. Despite this limitation, this exploratory study provides excellent data by which future studies can be appropriately powered.

In closing, until the long-term effects of these transfers on the developing bone are better understood, the investigators feel that these findings support the current recommendations to hold off on tendon transfer until the ossification center appears. However, as needle boring caused very minimal damage, alternative, less invasive methods of tendon transfer and fixation such as suture anchors or surface attachments may allow these transfers to be performed without apophyseal damage. The long-term effects of these transfers will be the focus of the next stage of future investigation.

## Electronic supplementary material

Below is the link to the electronic supplementary material.

The instructional slide and images assessed by the reviewers scoring the samples (PPTX 63532 kb)
