# Supplementary material for: Tendon transfer to unossified bone in a porcine model: potential implications for early tibialis anterior tendon transfers in children with clubfeet
Source: J Child Orthop. 2016 Nov 30;10(6):705–14. doi: 10.1007/s11832-016-0799-4 (PMC5145849; doi:10.1007/s11832-016-0799-4)
Supplement: Supplementary file 1 — The instructional slide and images assessed by the reviewers scoring the samples (PPTX 63532 kb) [file 11832_2016_799_MOESM1_ESM.pptx]

## Slide 1
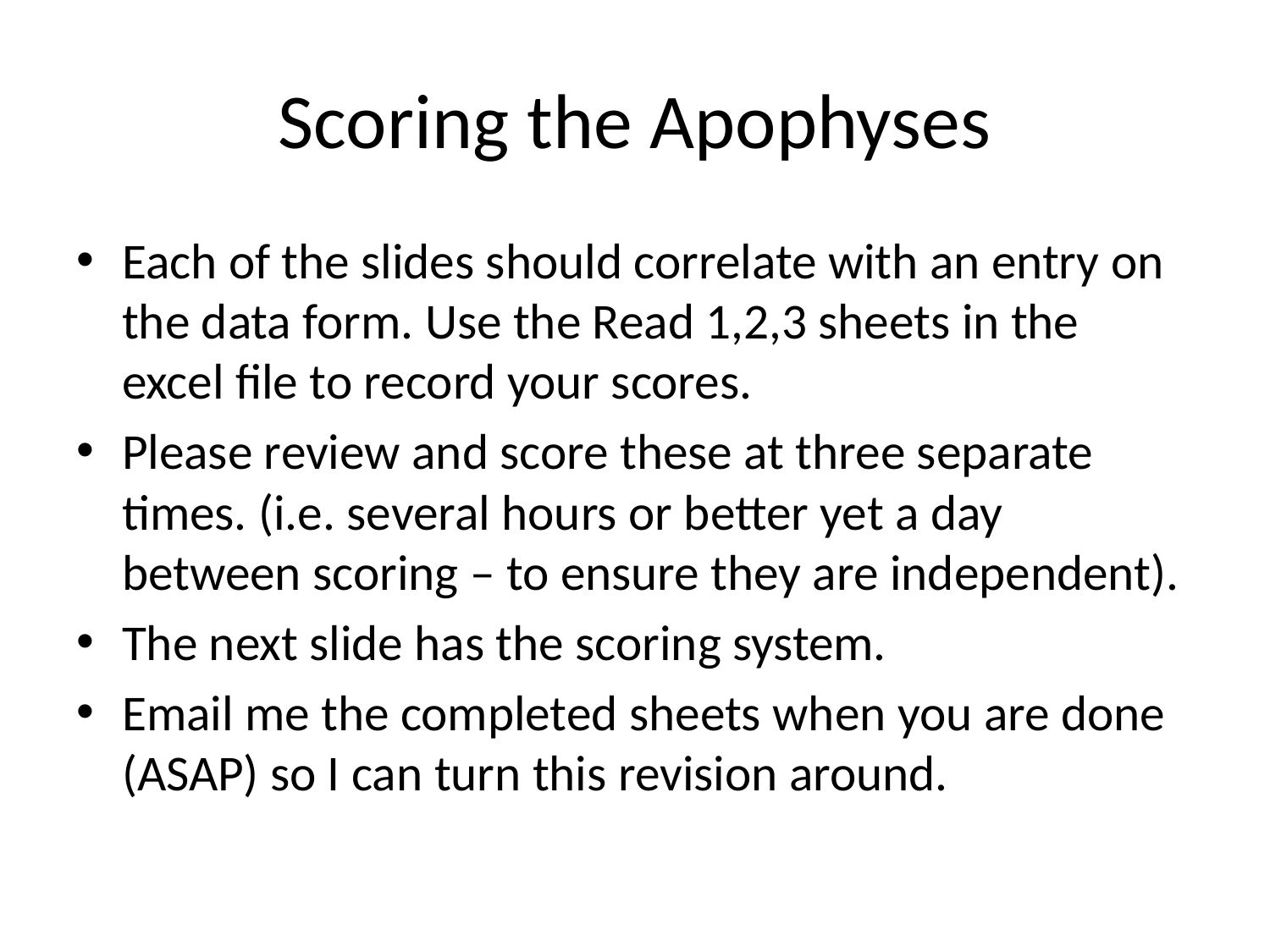

# Scoring the Apophyses
Each of the slides should correlate with an entry on the data form. Use the Read 1,2,3 sheets in the excel file to record your scores.
Please review and score these at three separate times. (i.e. several hours or better yet a day between scoring – to ensure they are independent).
The next slide has the scoring system.
Email me the completed sheets when you are done (ASAP) so I can turn this revision around.

## Slide 2
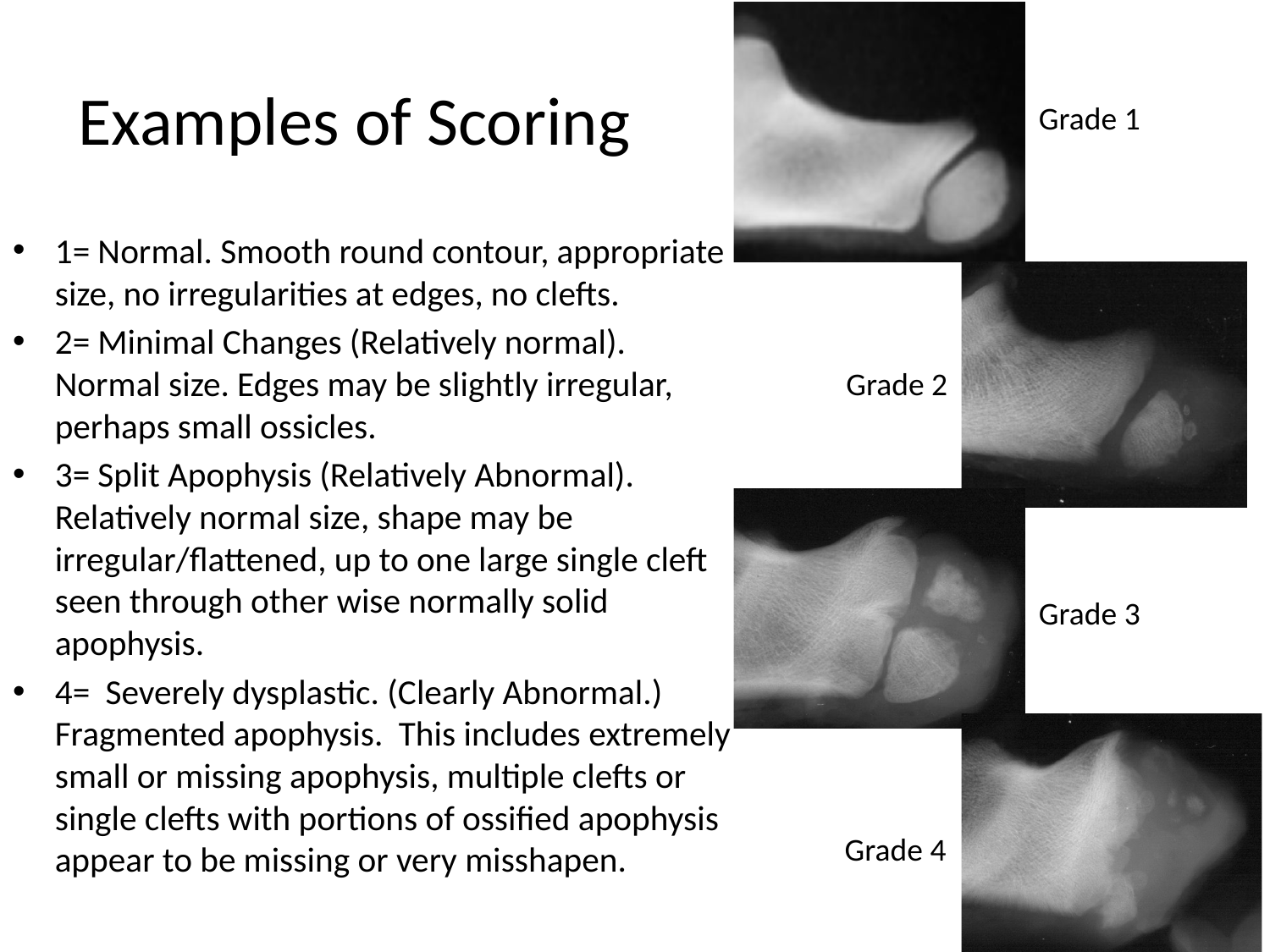

# Examples of Scoring
Grade 1
1= Normal. Smooth round contour, appropriate size, no irregularities at edges, no clefts.
2= Minimal Changes (Relatively normal). Normal size. Edges may be slightly irregular, perhaps small ossicles.
3= Split Apophysis (Relatively Abnormal). Relatively normal size, shape may be irregular/flattened, up to one large single cleft seen through other wise normally solid apophysis.
4= Severely dysplastic. (Clearly Abnormal.) Fragmented apophysis. This includes extremely small or missing apophysis, multiple clefts or single clefts with portions of ossified apophysis appear to be missing or very misshapen.
Grade 2
Grade 3
Grade 4

## Slide 3
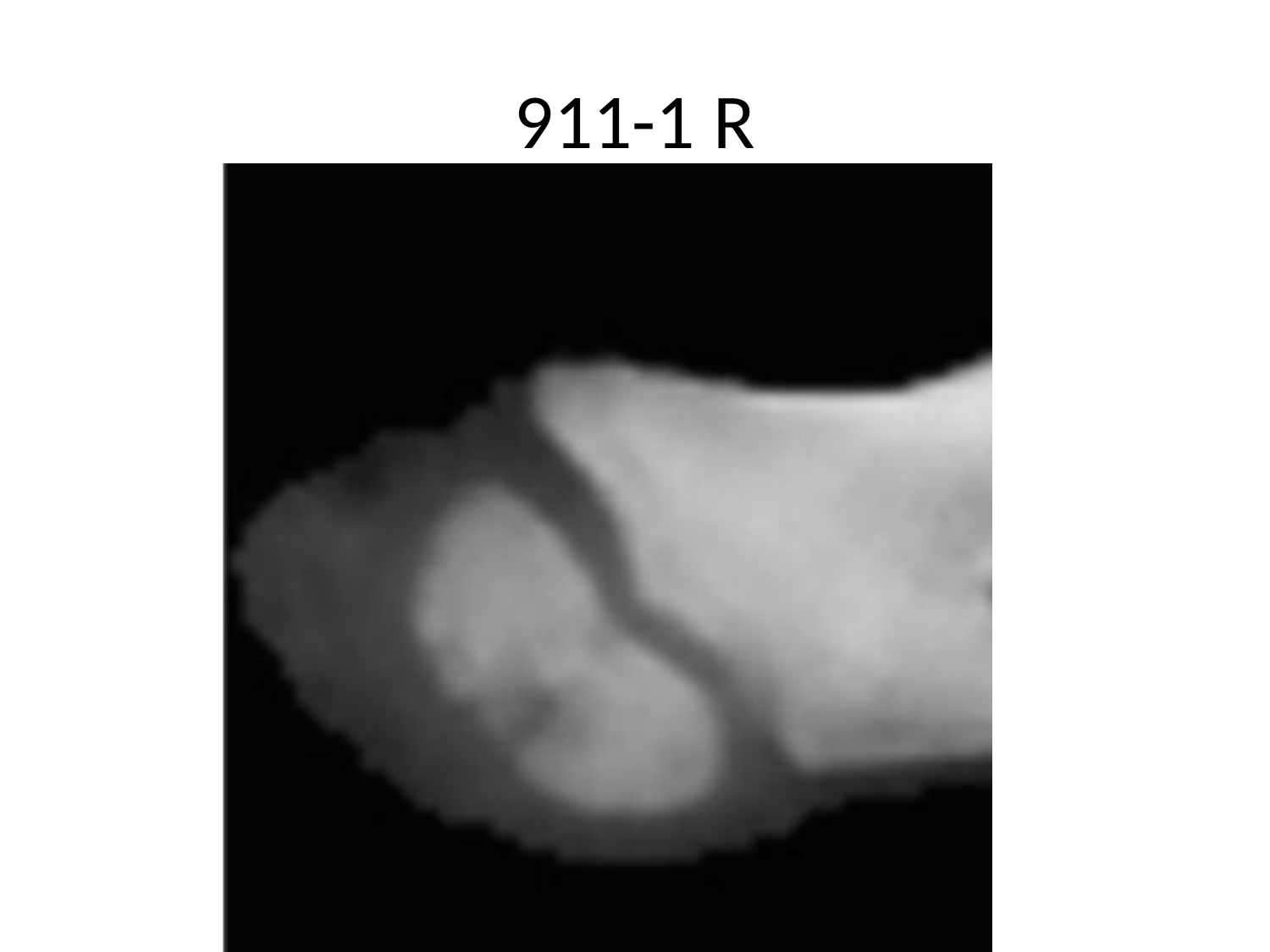

# 911-1 R

## Slide 4
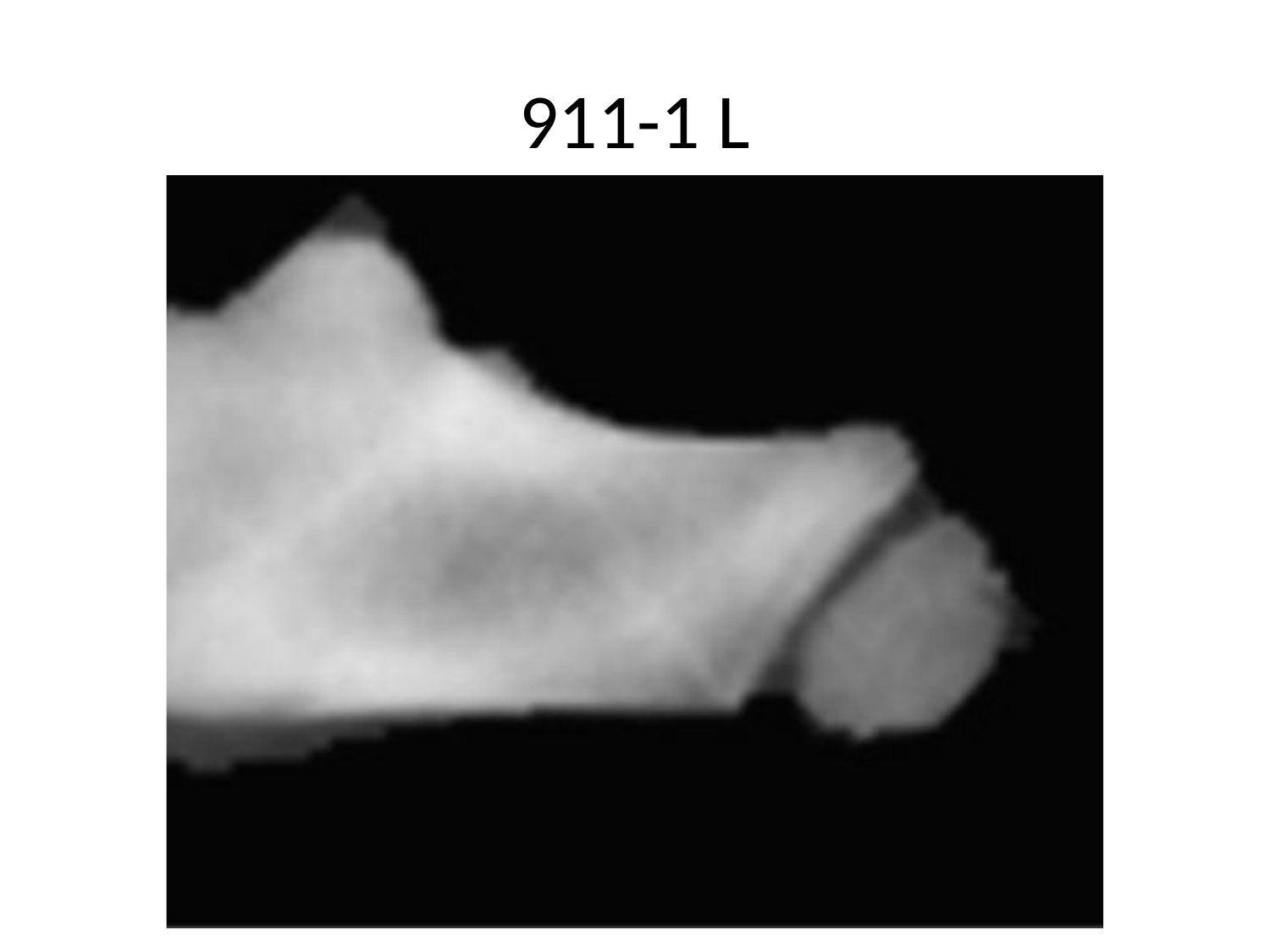

# 911-1 L

## Slide 5
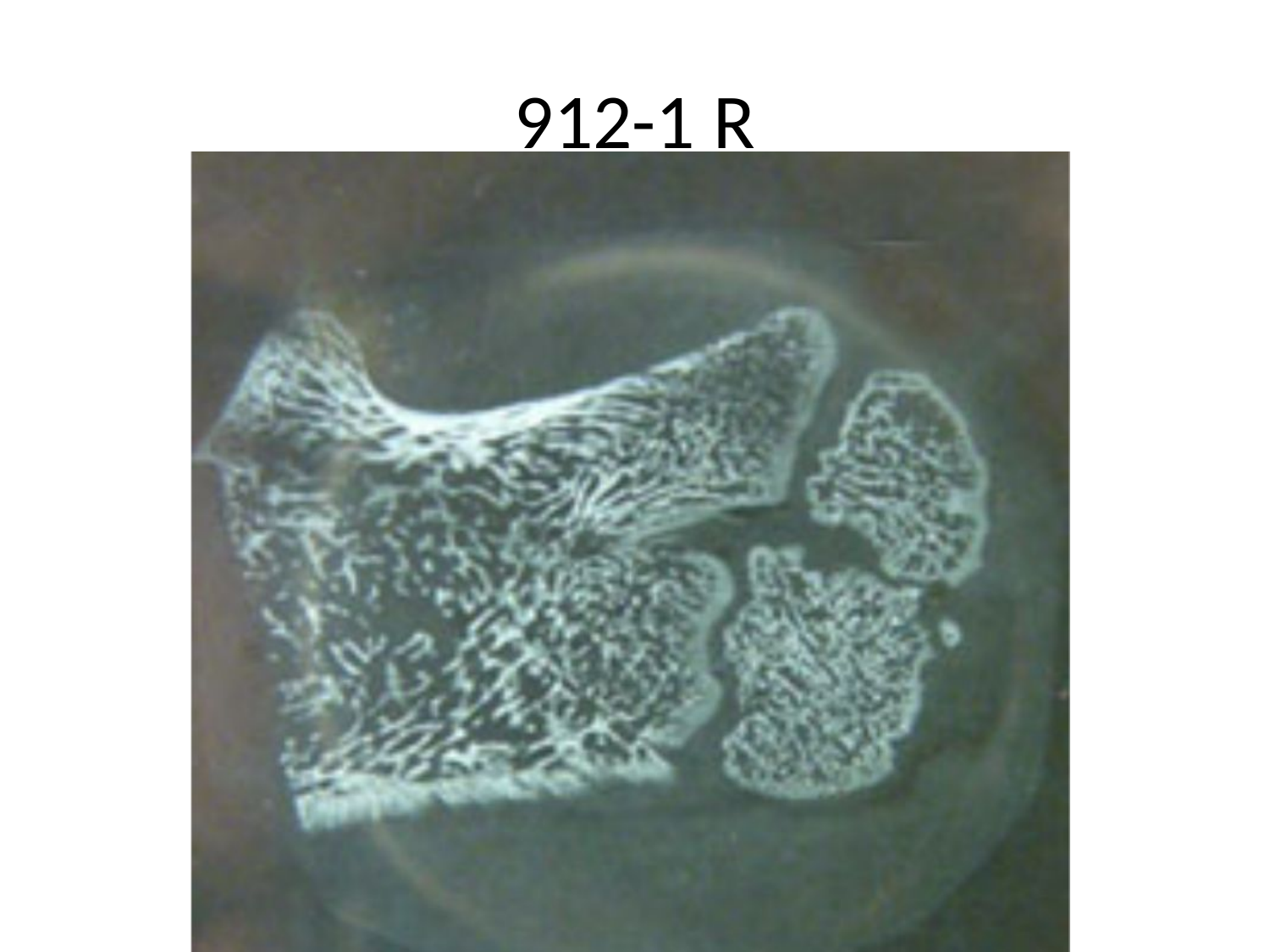

# 912-1 R

## Slide 6
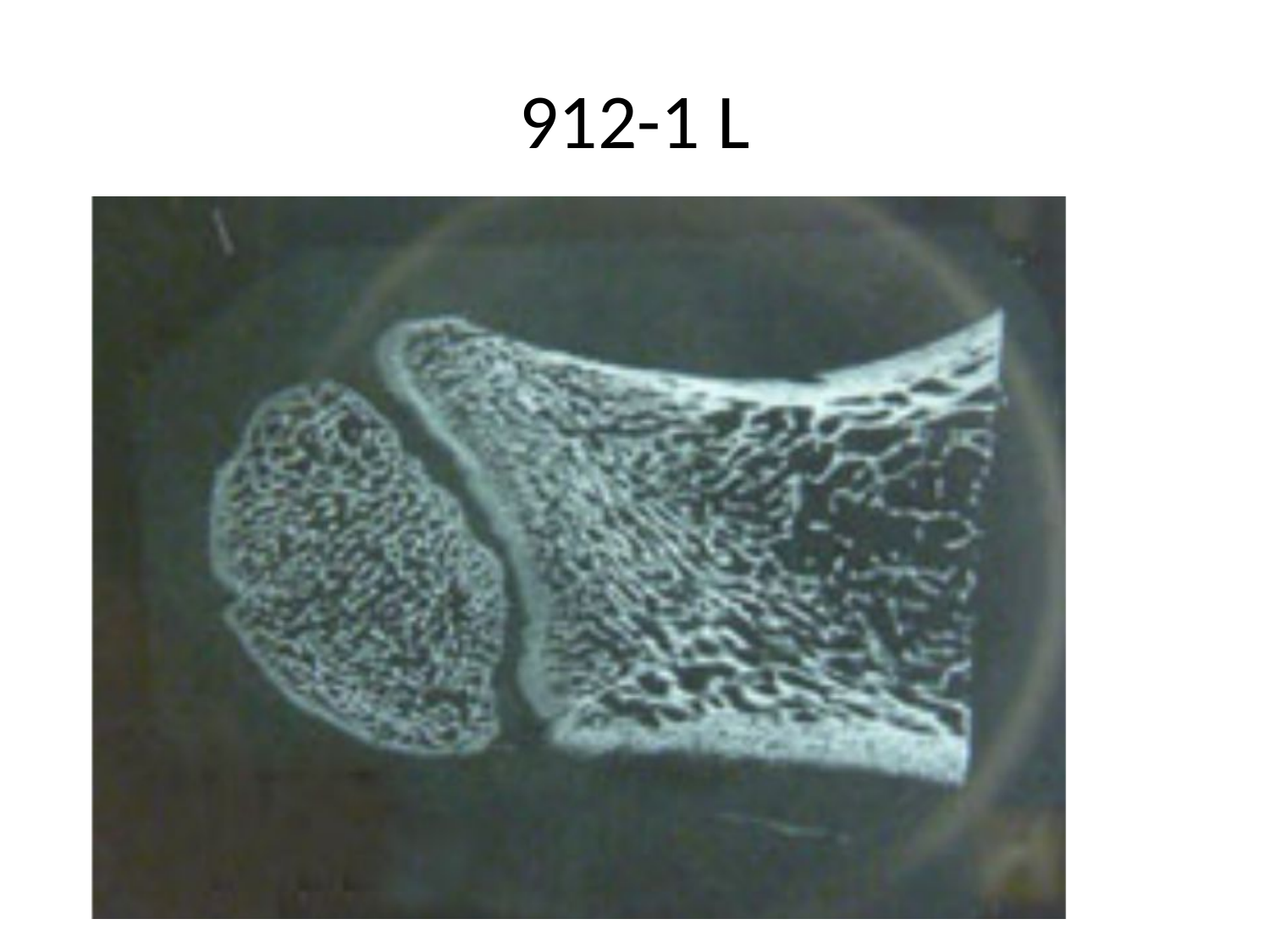

# 912-1 L

## Slide 7
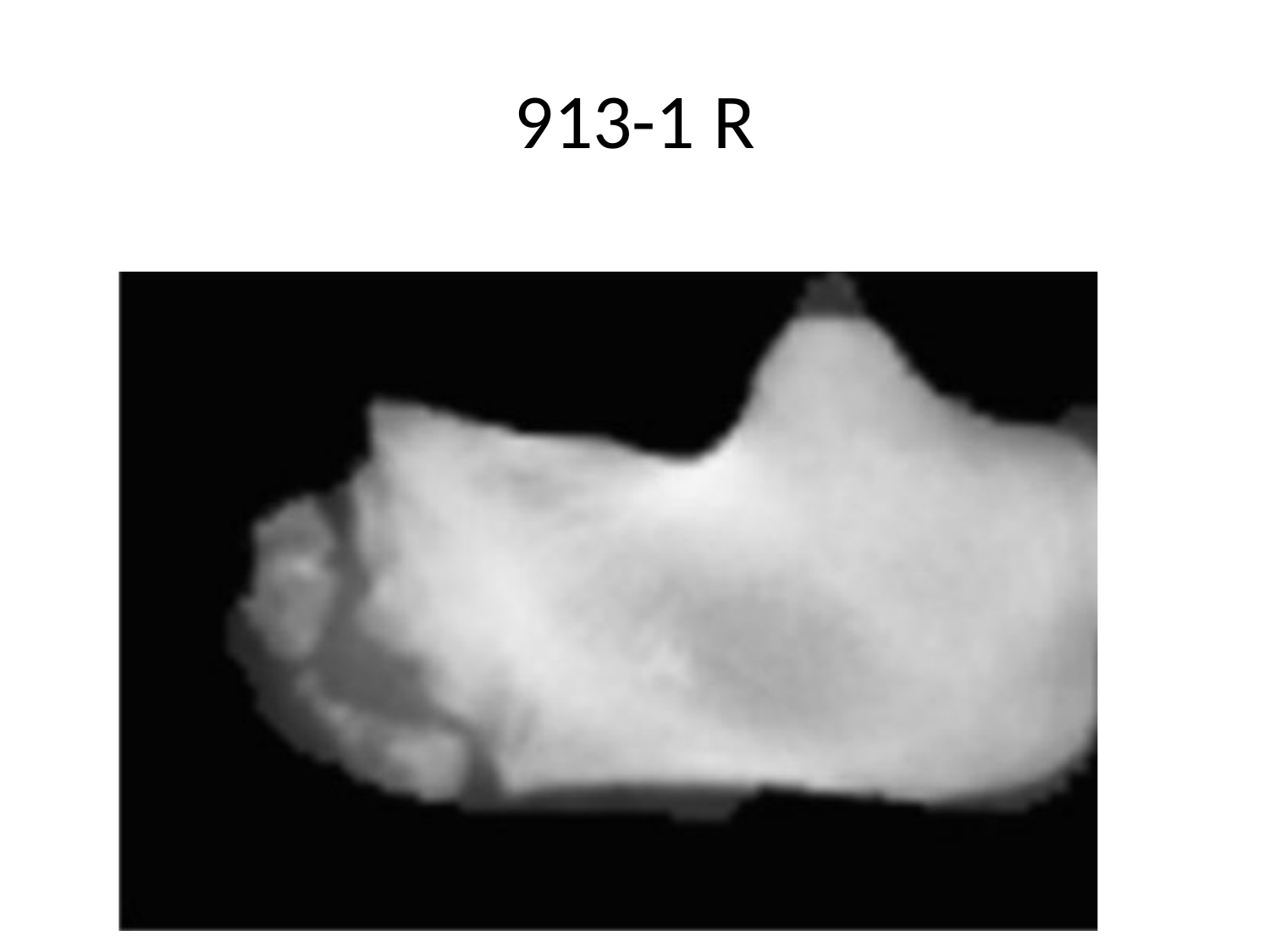

# 913-1 R

## Slide 8
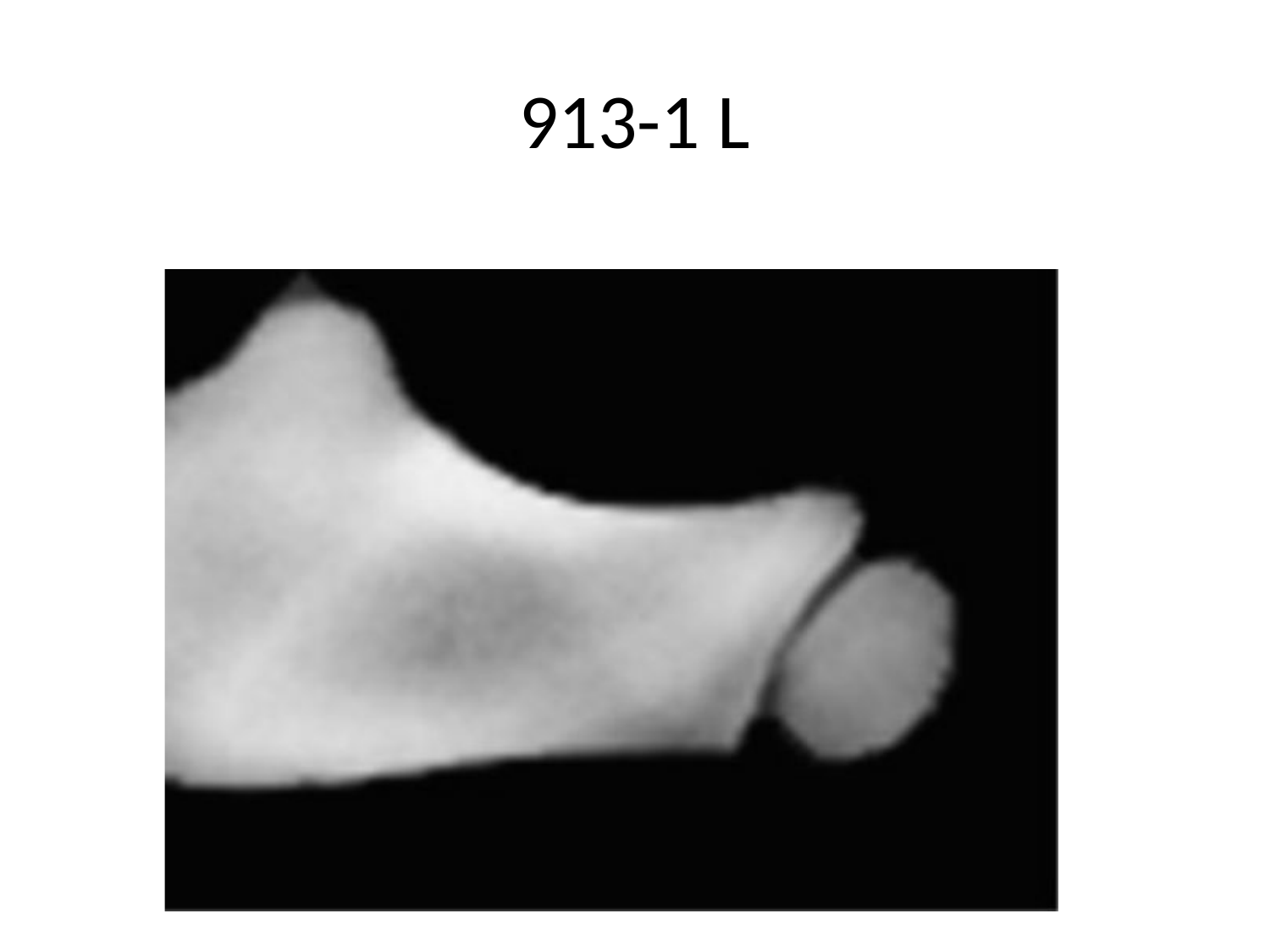

# 913-1 L

## Slide 9
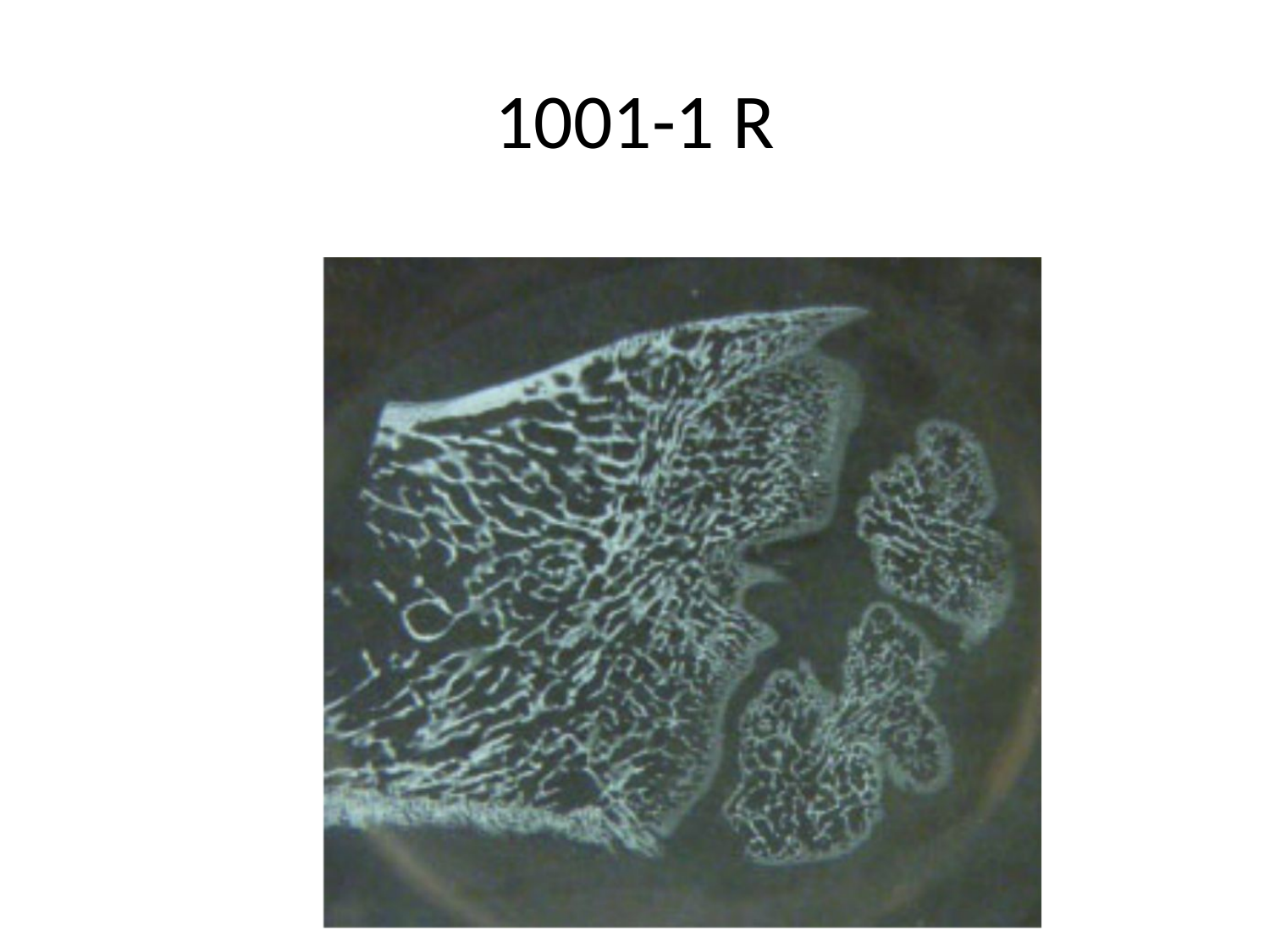

# 1001-1 R

## Slide 10
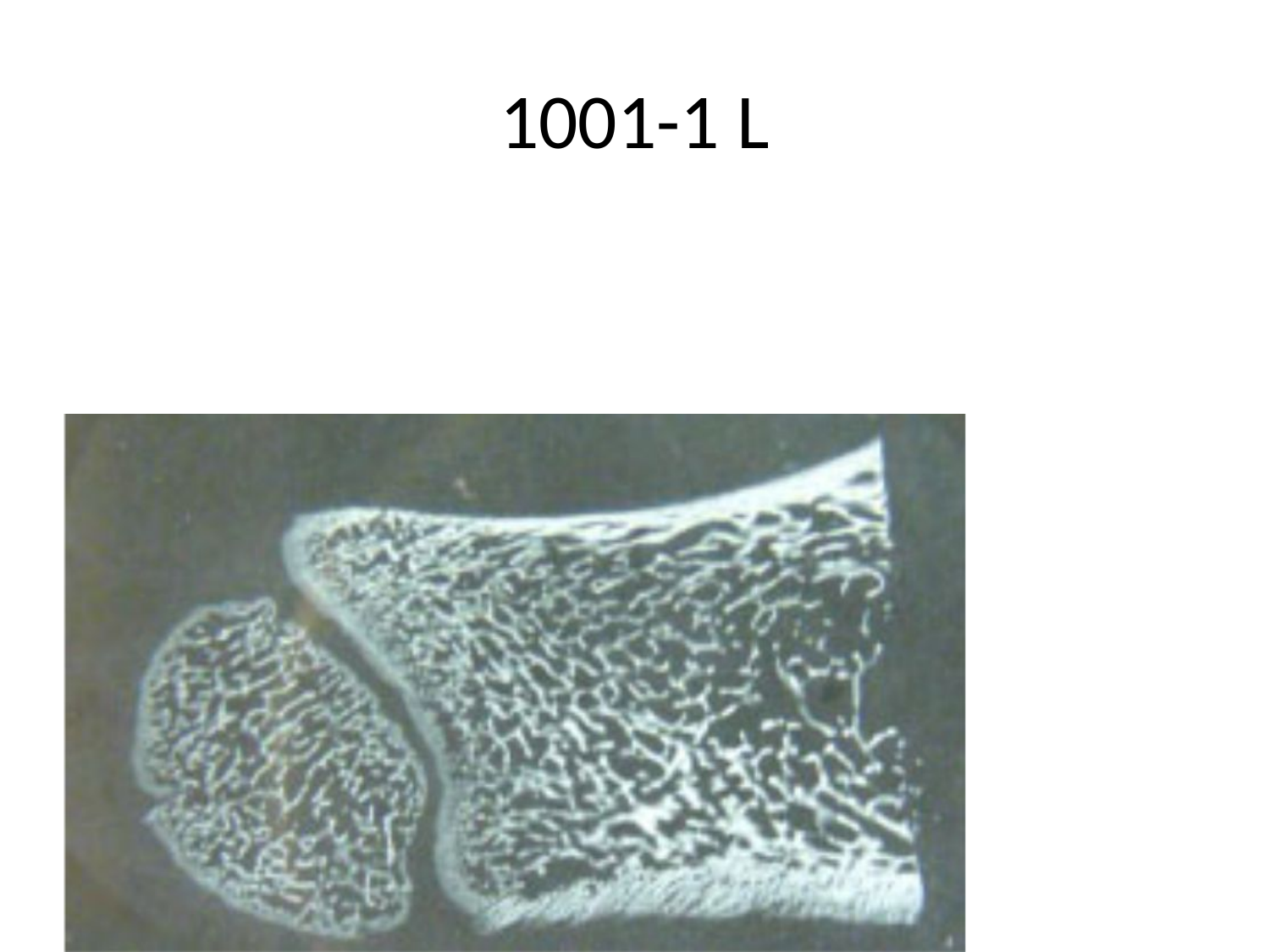

# 1001-1 L

## Slide 11
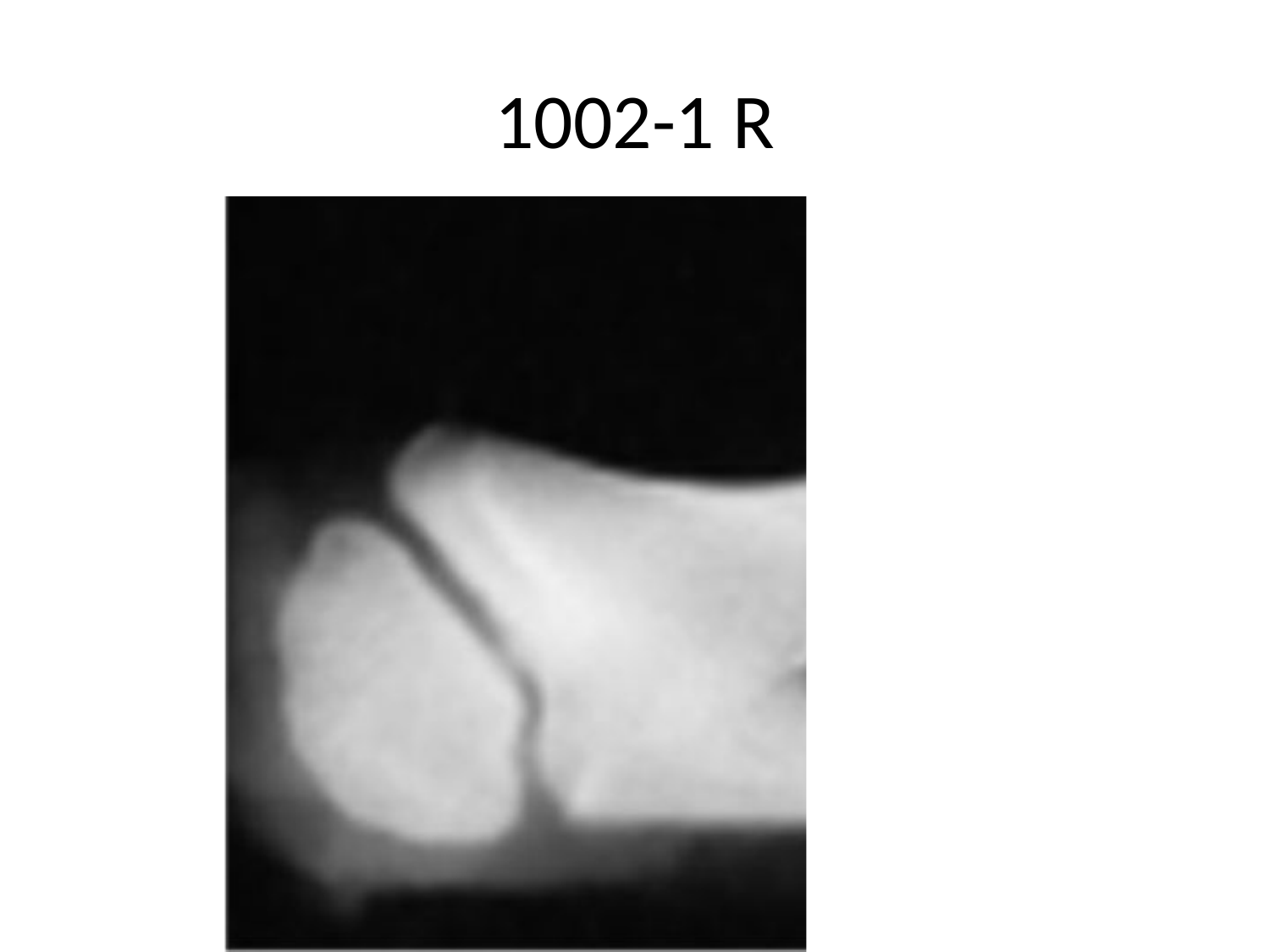

# 1002-1 R

## Slide 12
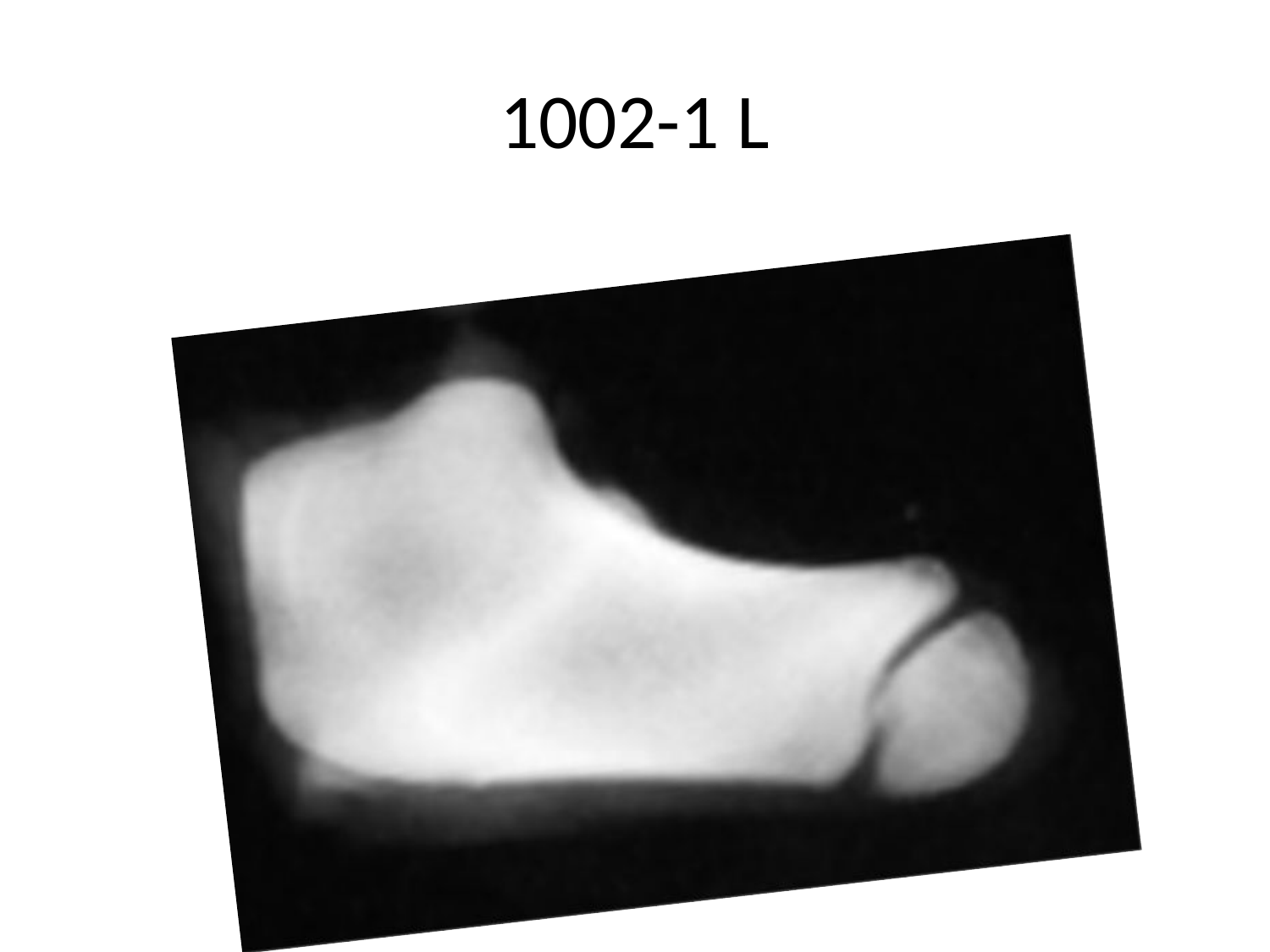

# 1002-1 L

## Slide 13
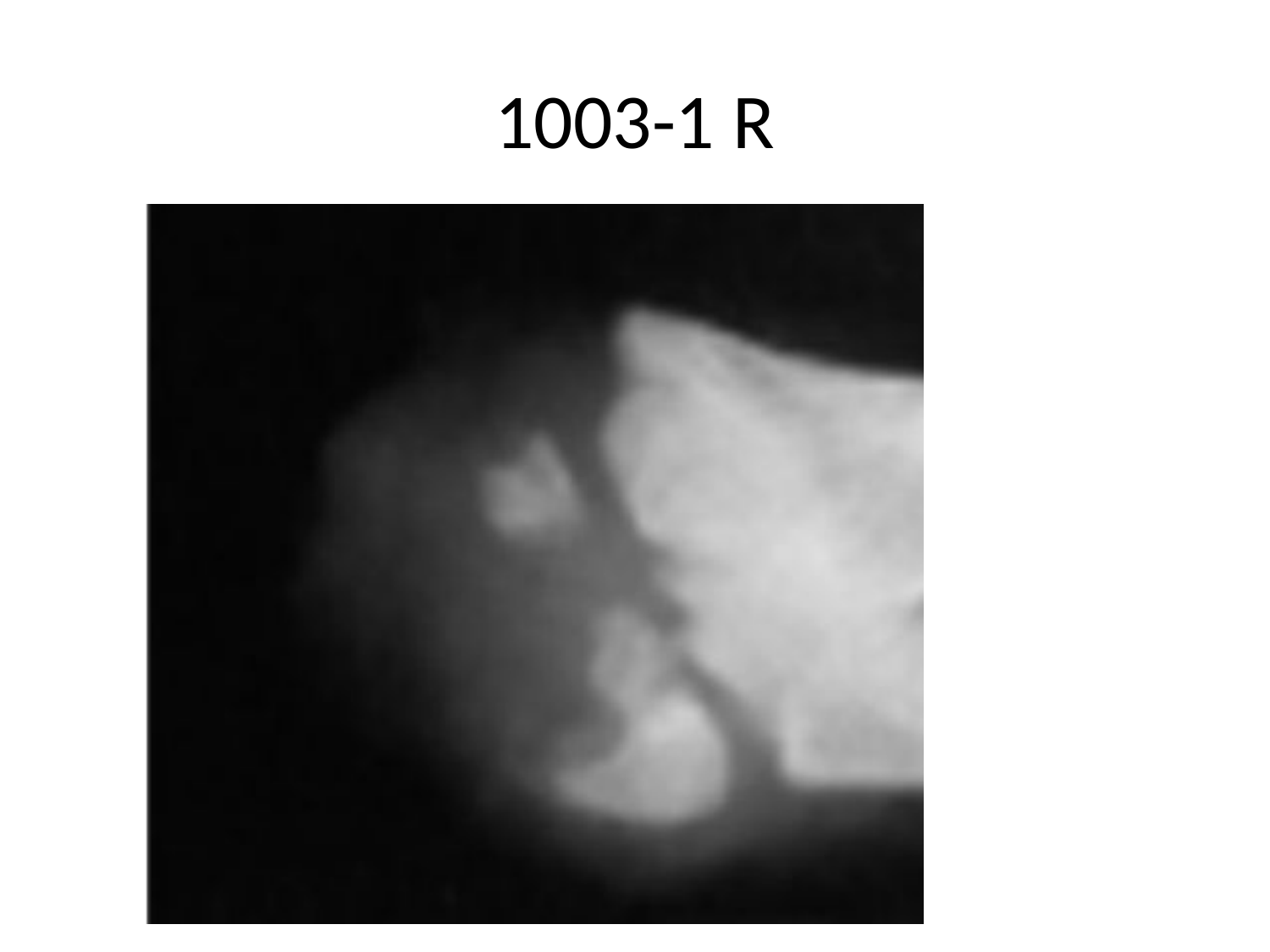

# 1003-1 R

## Slide 14
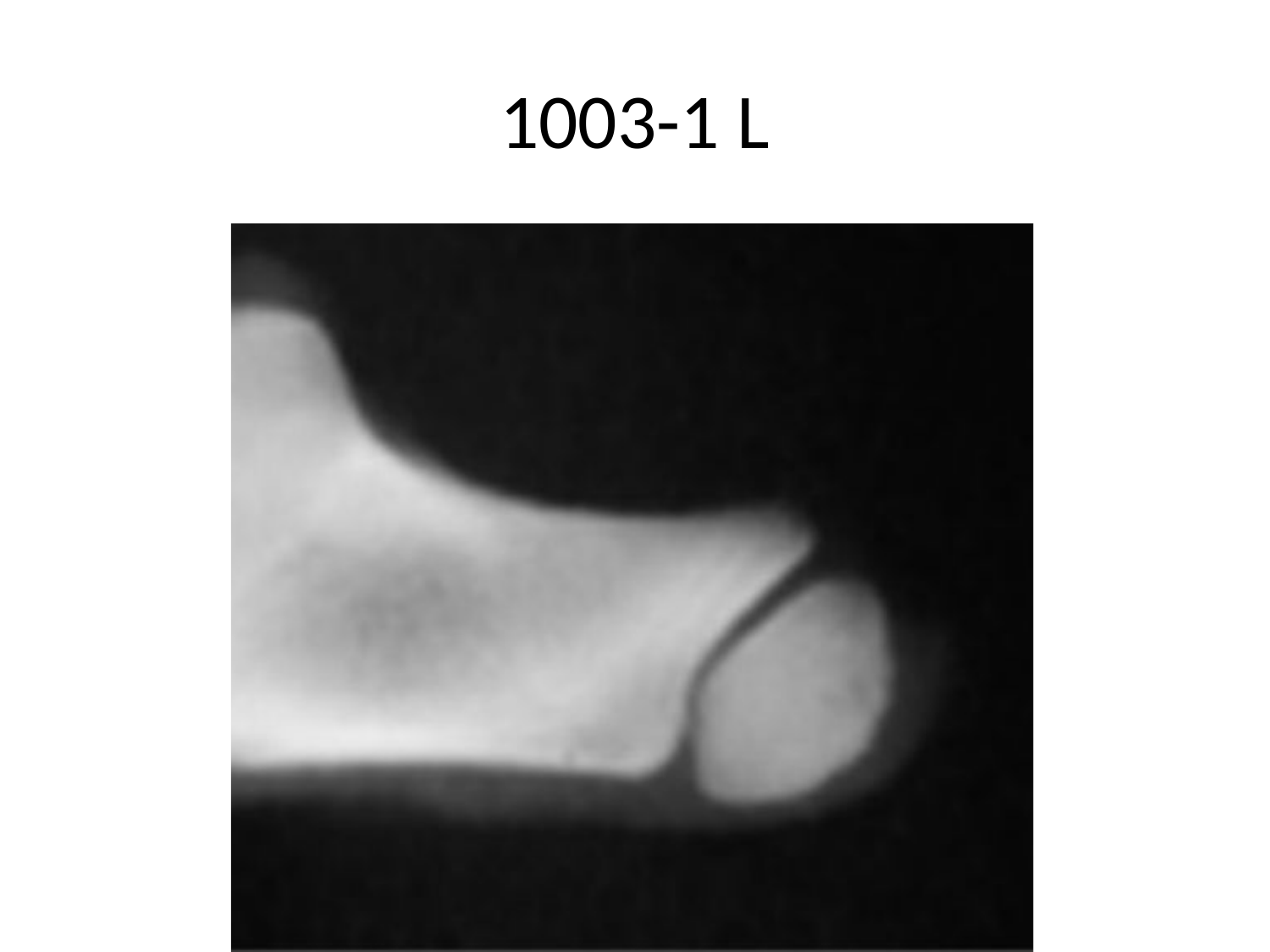

# 1003-1 L

## Slide 15
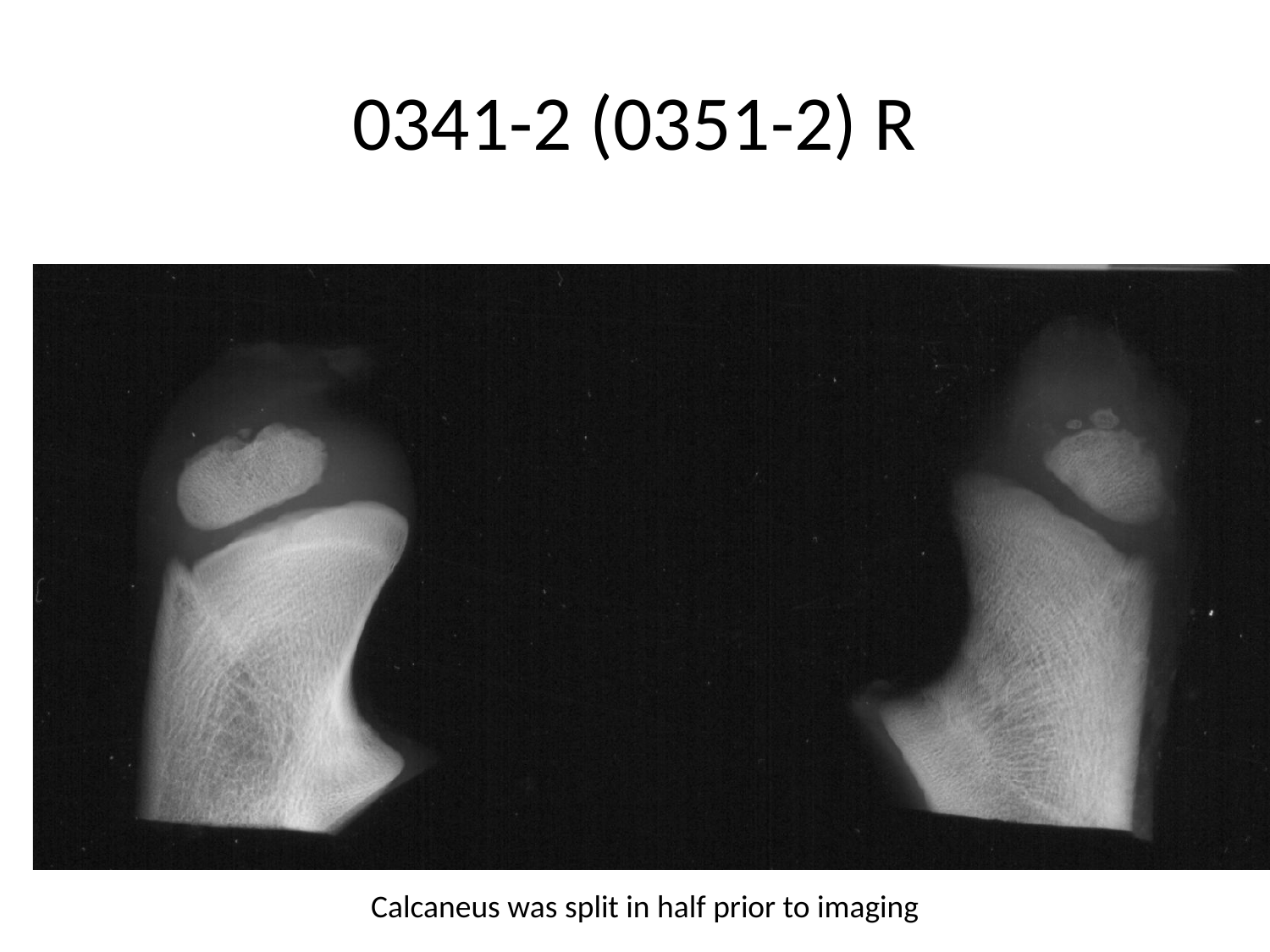

# 0341-2 (0351-2) R
Calcaneus was split in half prior to imaging

## Slide 16
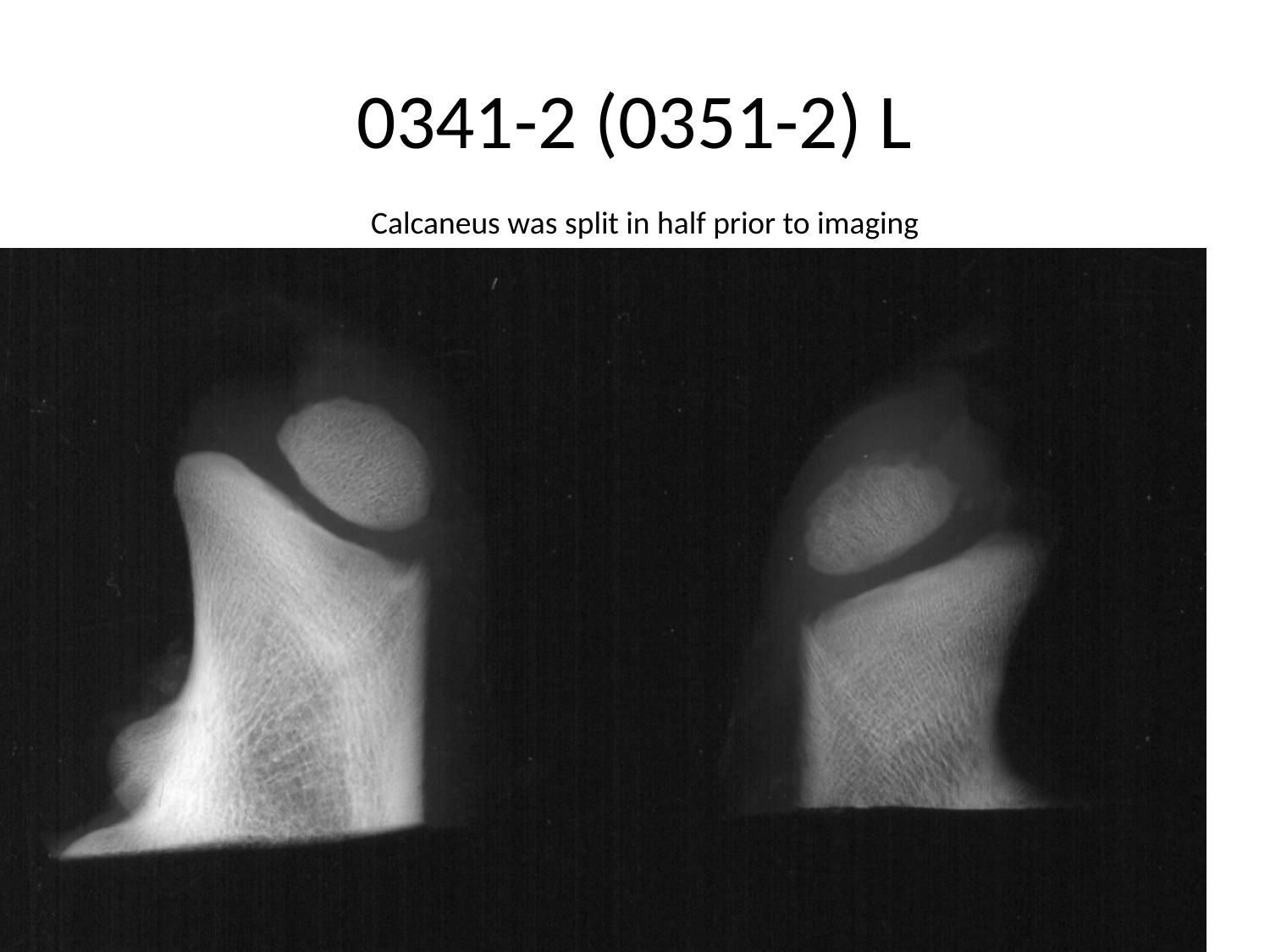

# 0341-2 (0351-2) L
Calcaneus was split in half prior to imaging

## Slide 17
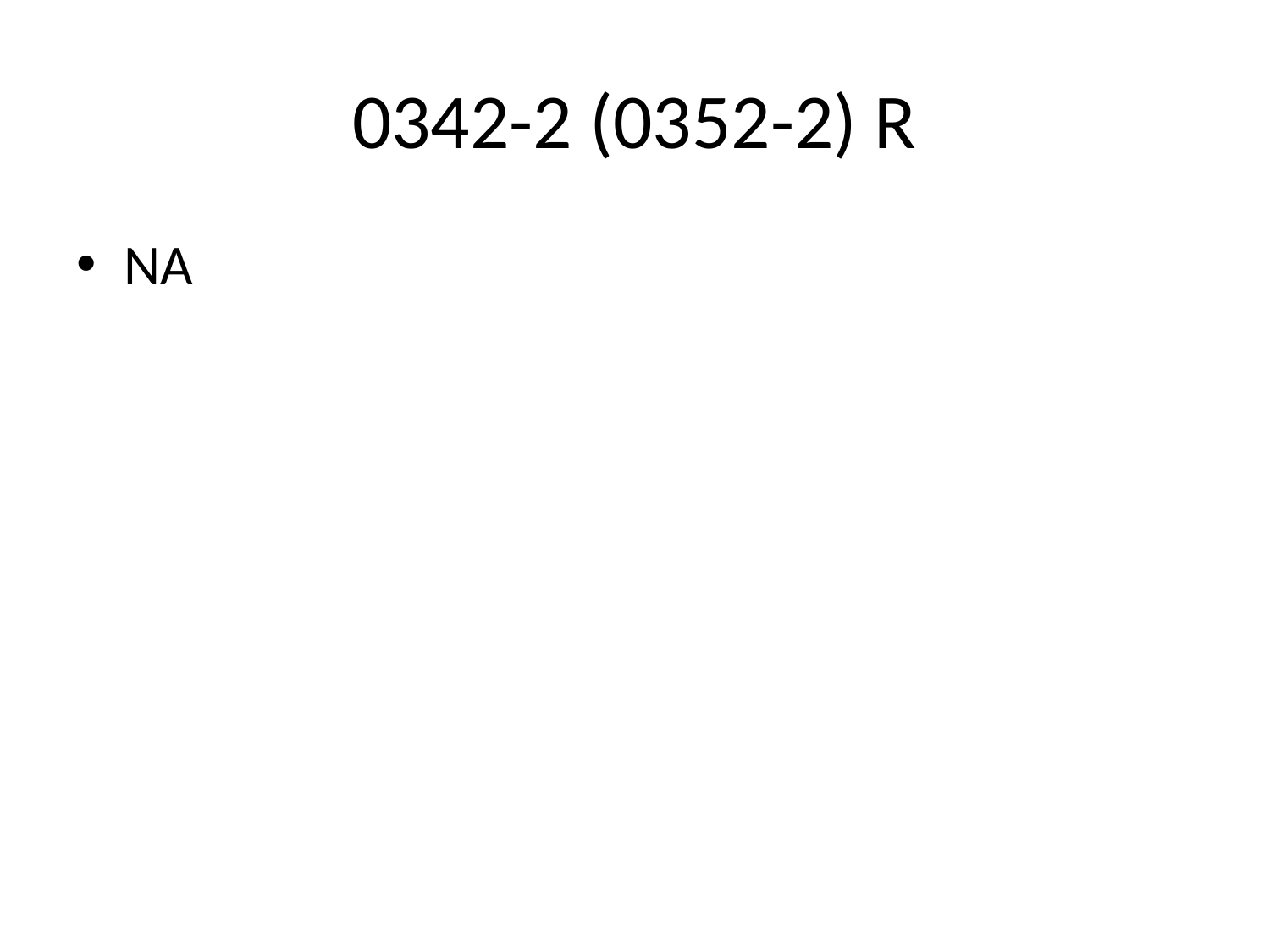

# 0342-2 (0352-2) R
NA

## Slide 18
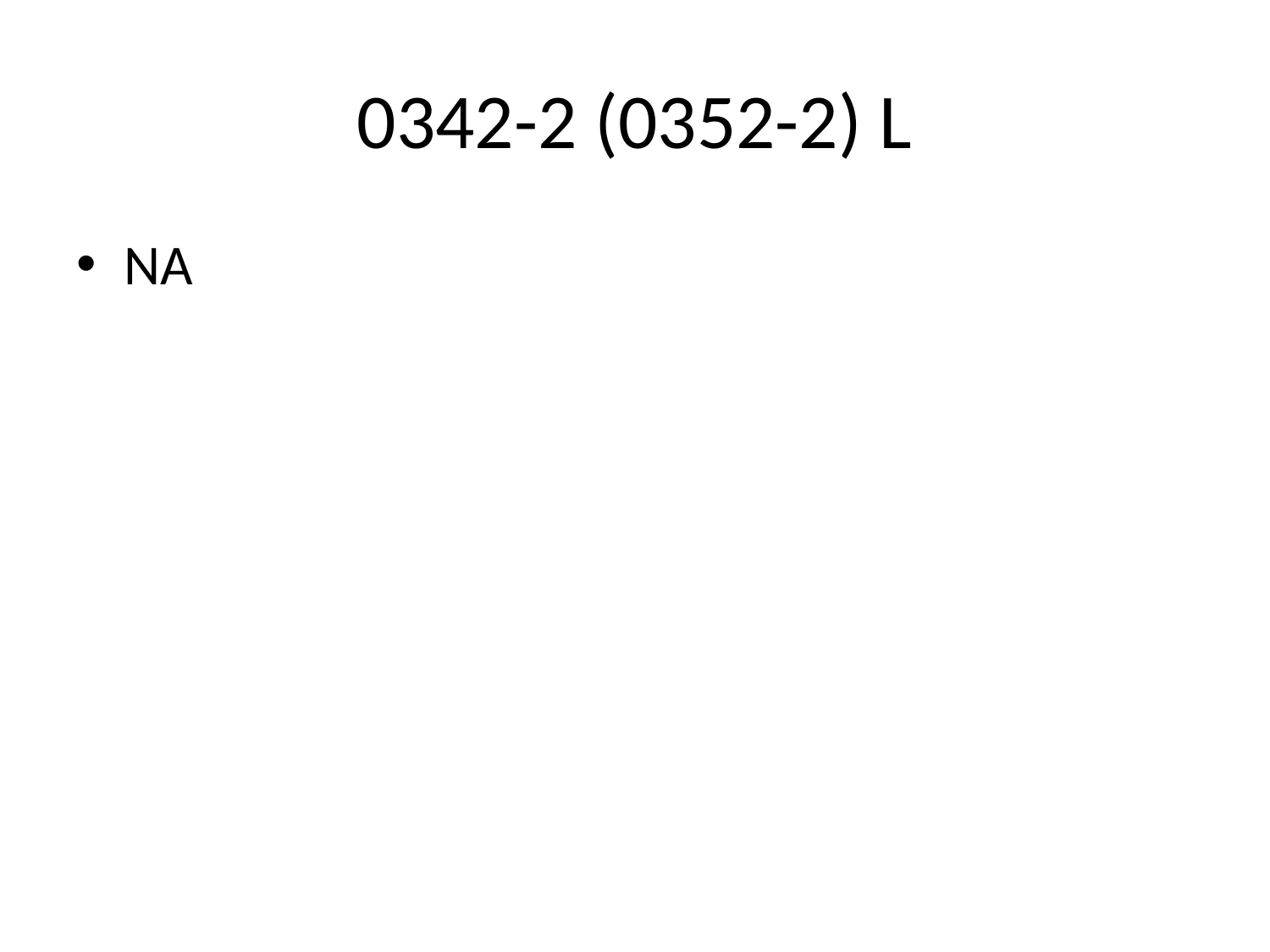

# 0342-2 (0352-2) L
NA

## Slide 19
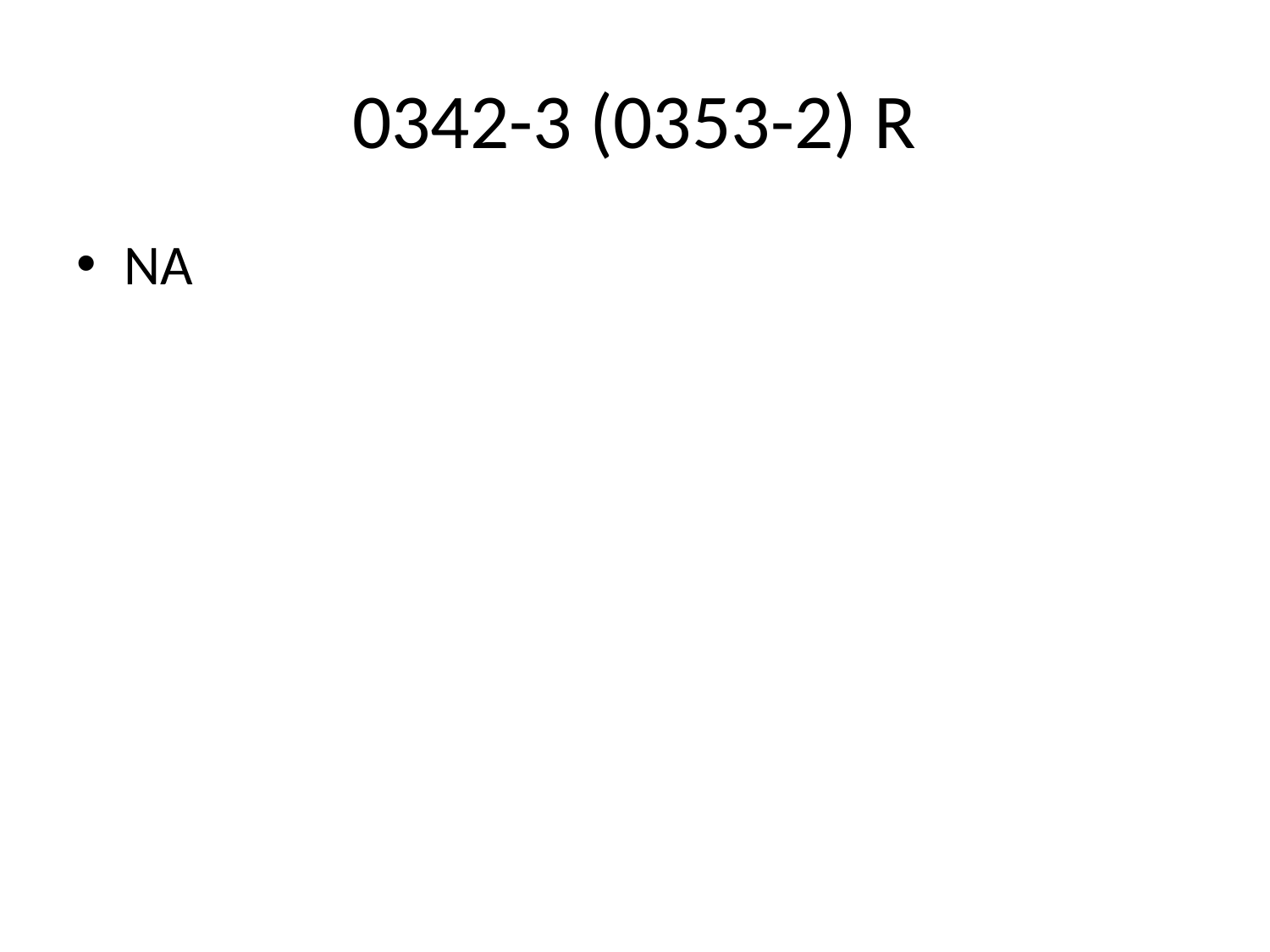

# 0342-3 (0353-2) R
NA

## Slide 20
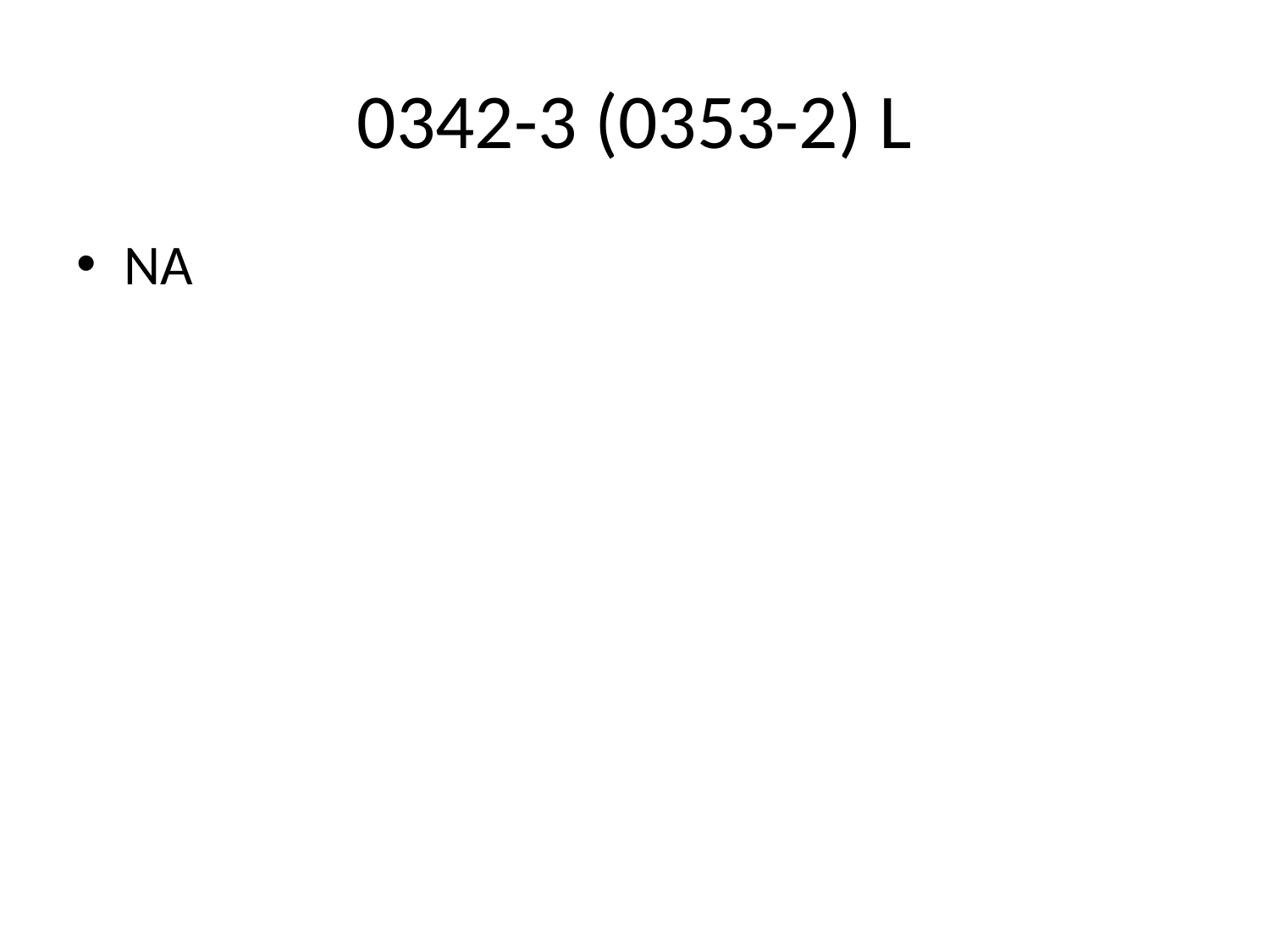

# 0342-3 (0353-2) L
NA

## Slide 21
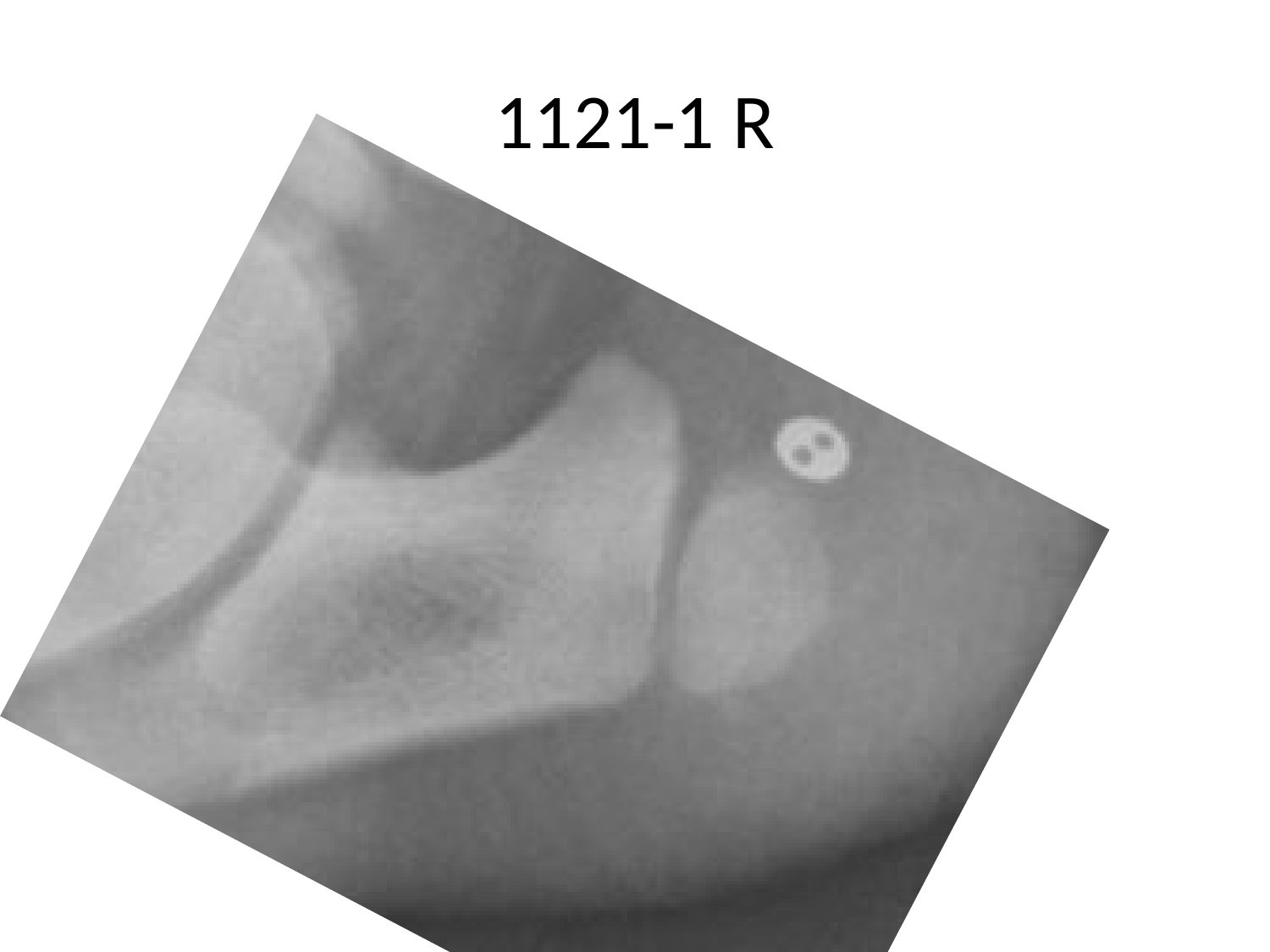

# 1121-1 R

## Slide 22
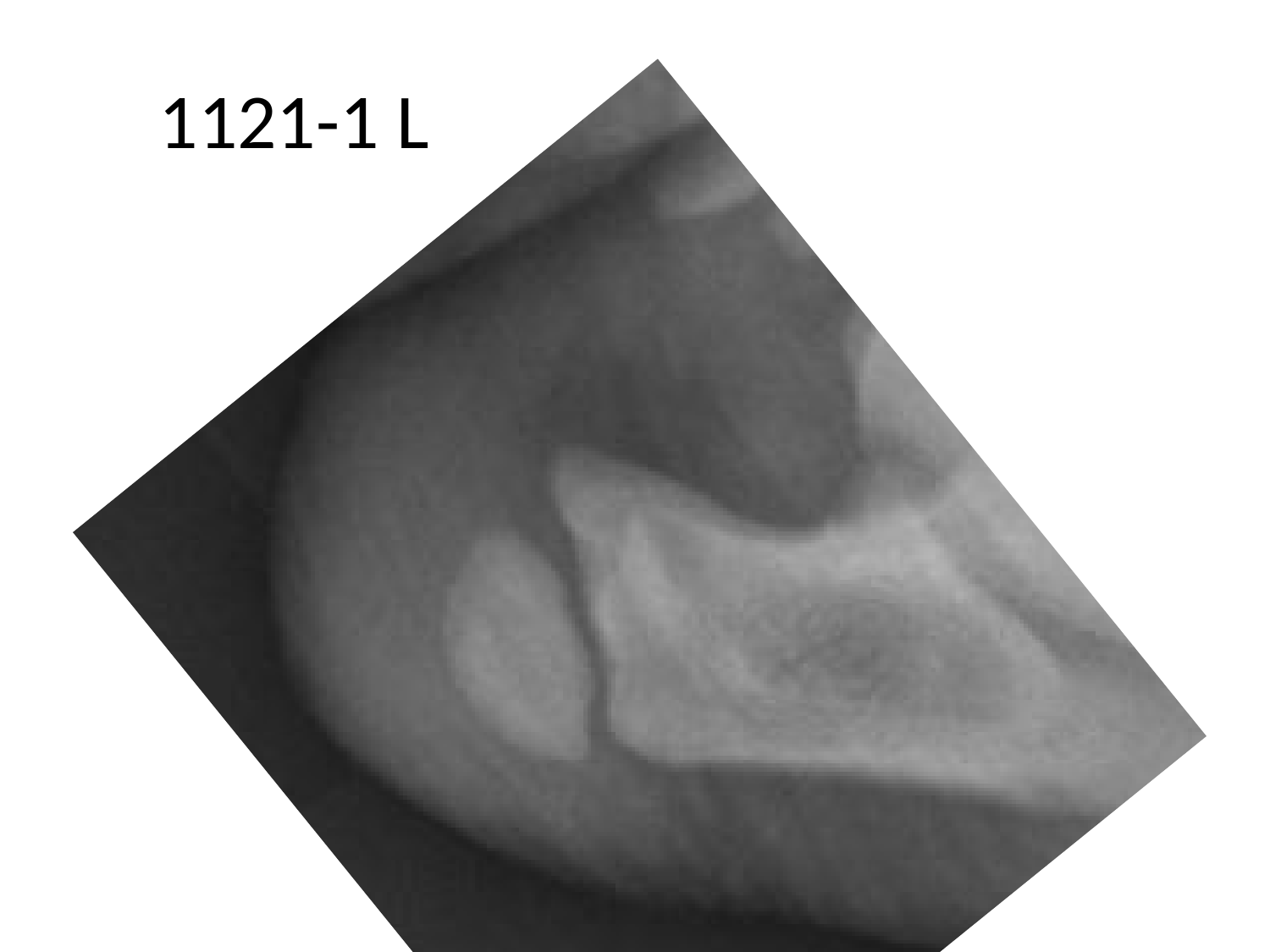

# 1121-1 L

## Slide 23
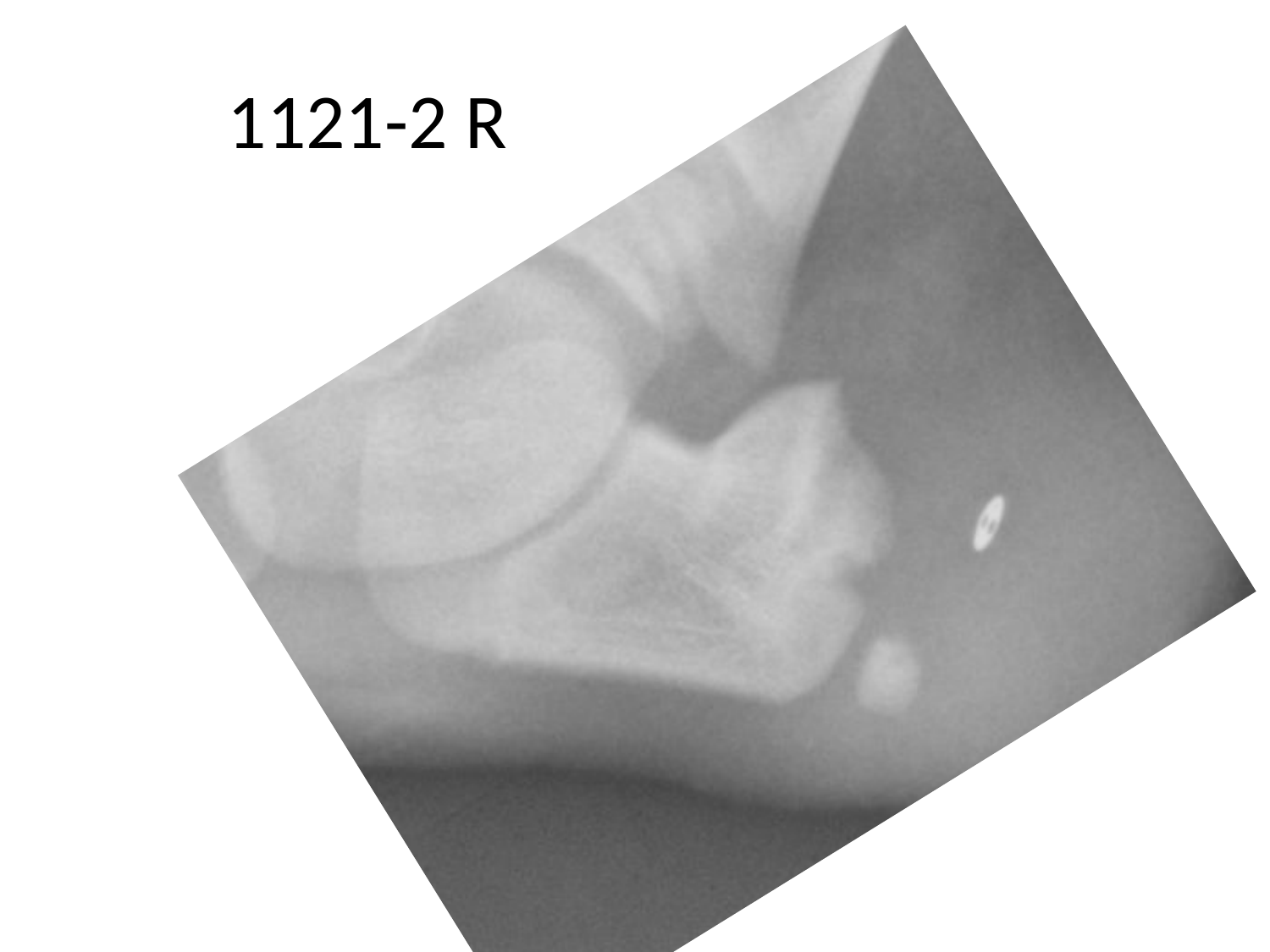

# 1121-2 R

## Slide 24
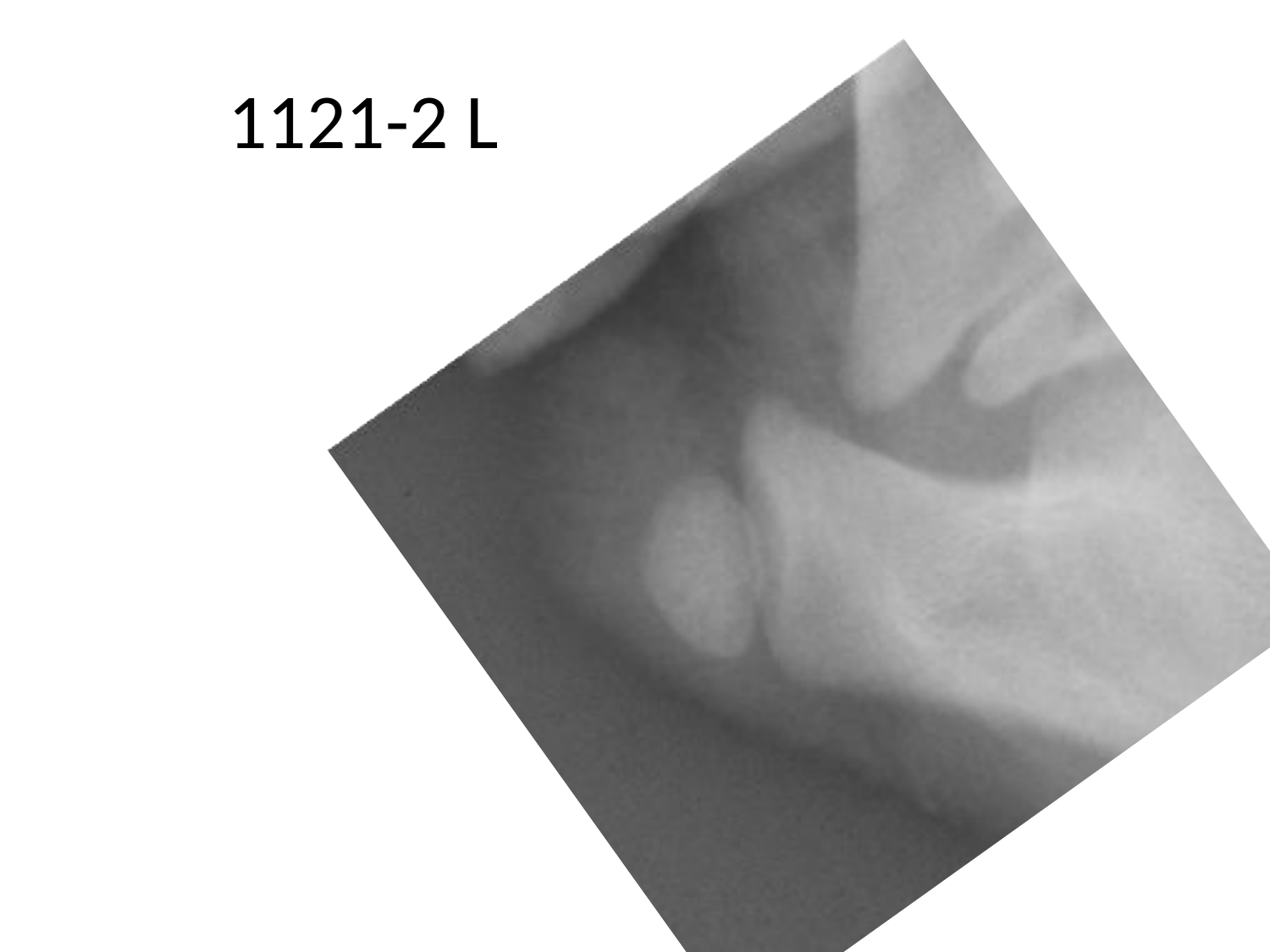

# 1121-2 L

## Slide 25
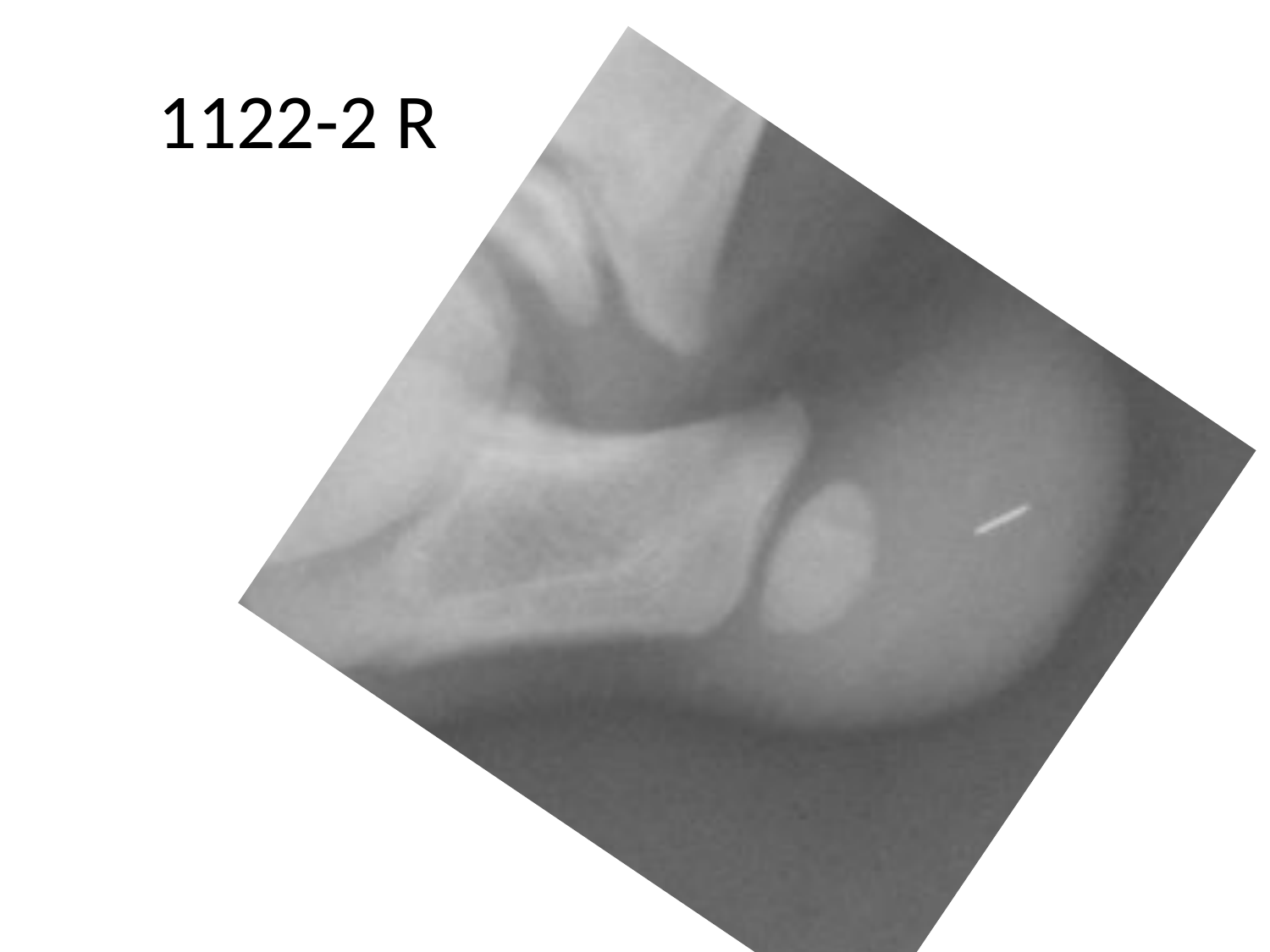

# 1122-2 R

## Slide 26
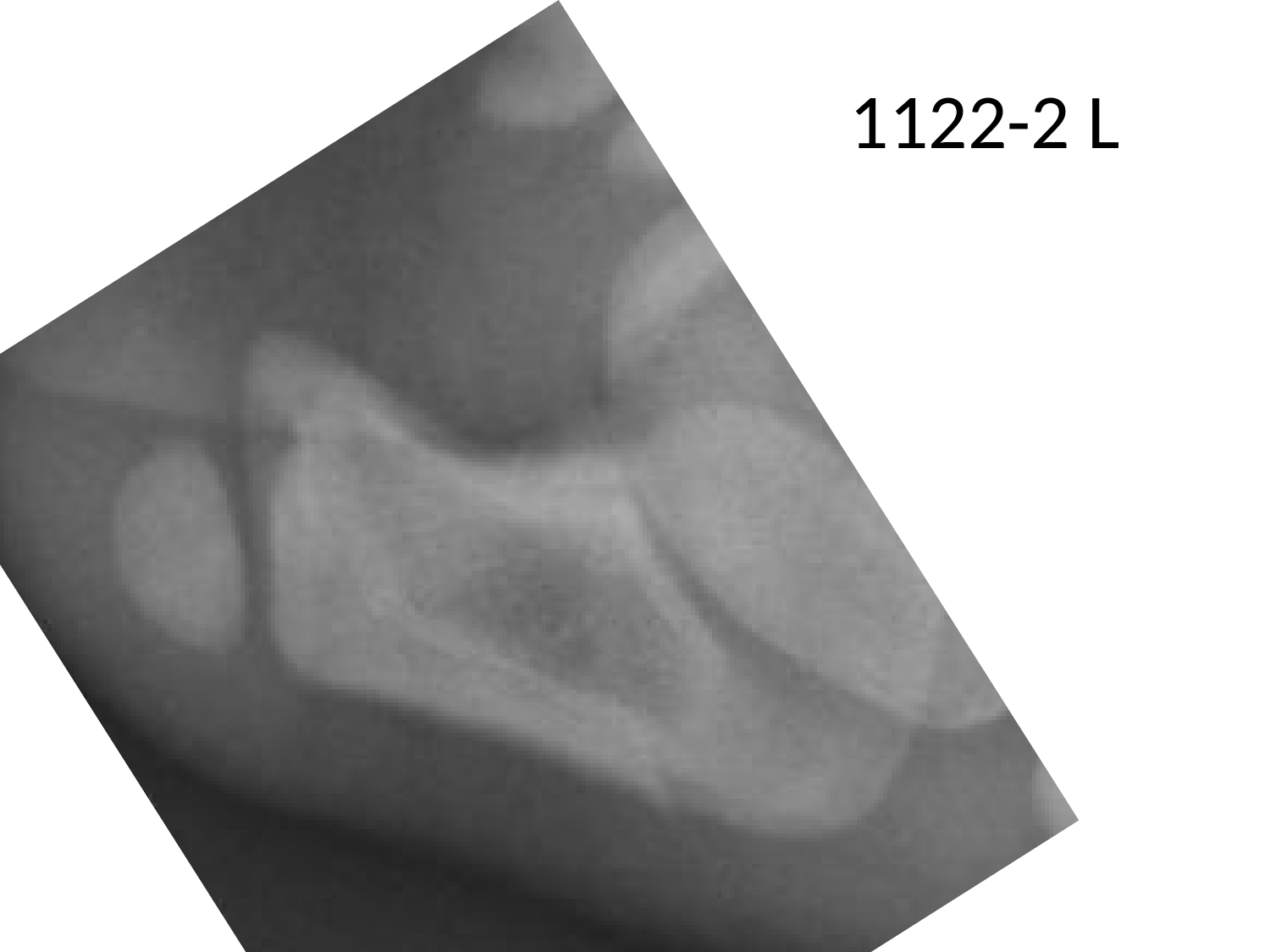

# 1122-2 L

## Slide 27
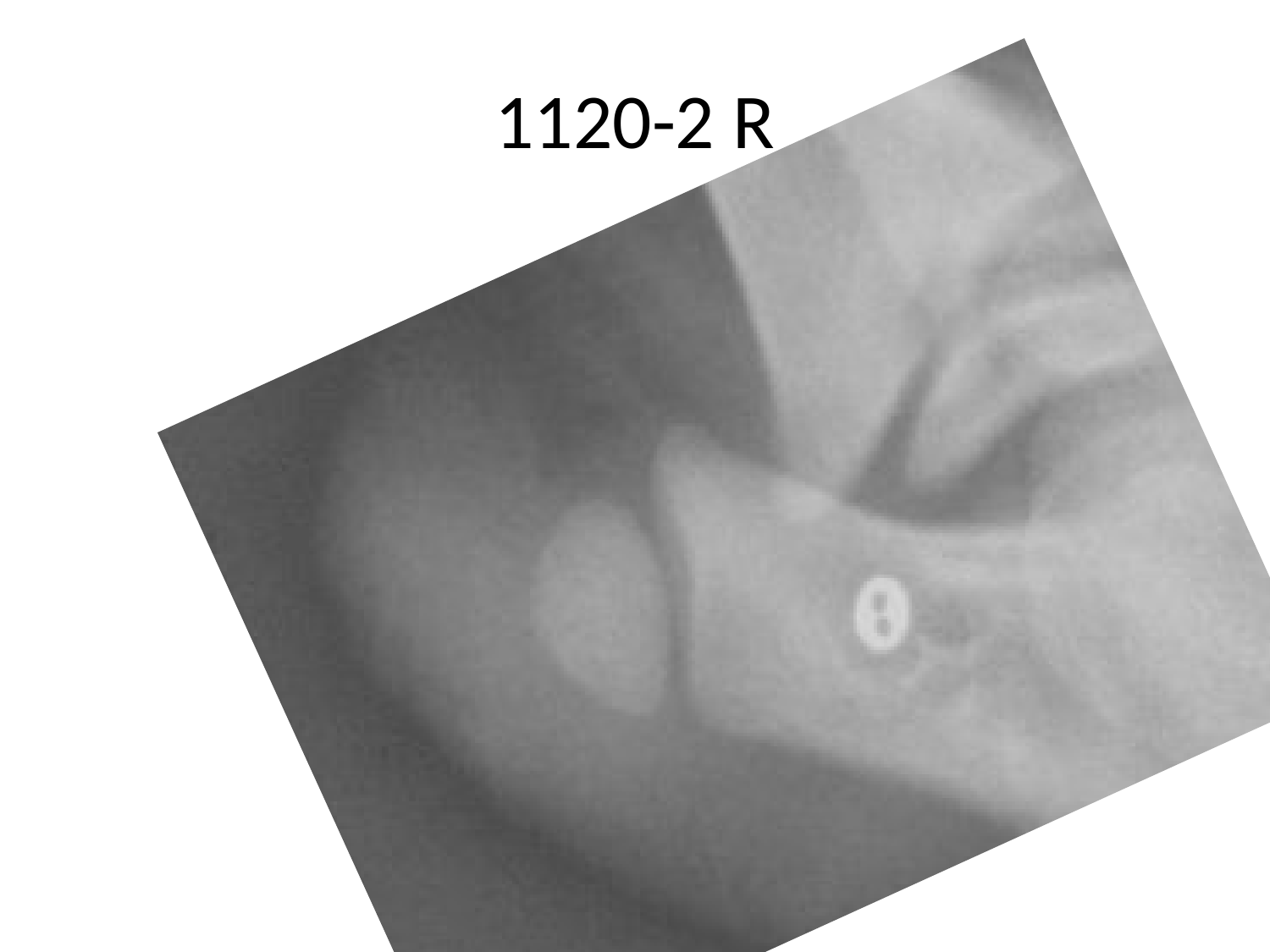

# 1120-2 R

## Slide 28
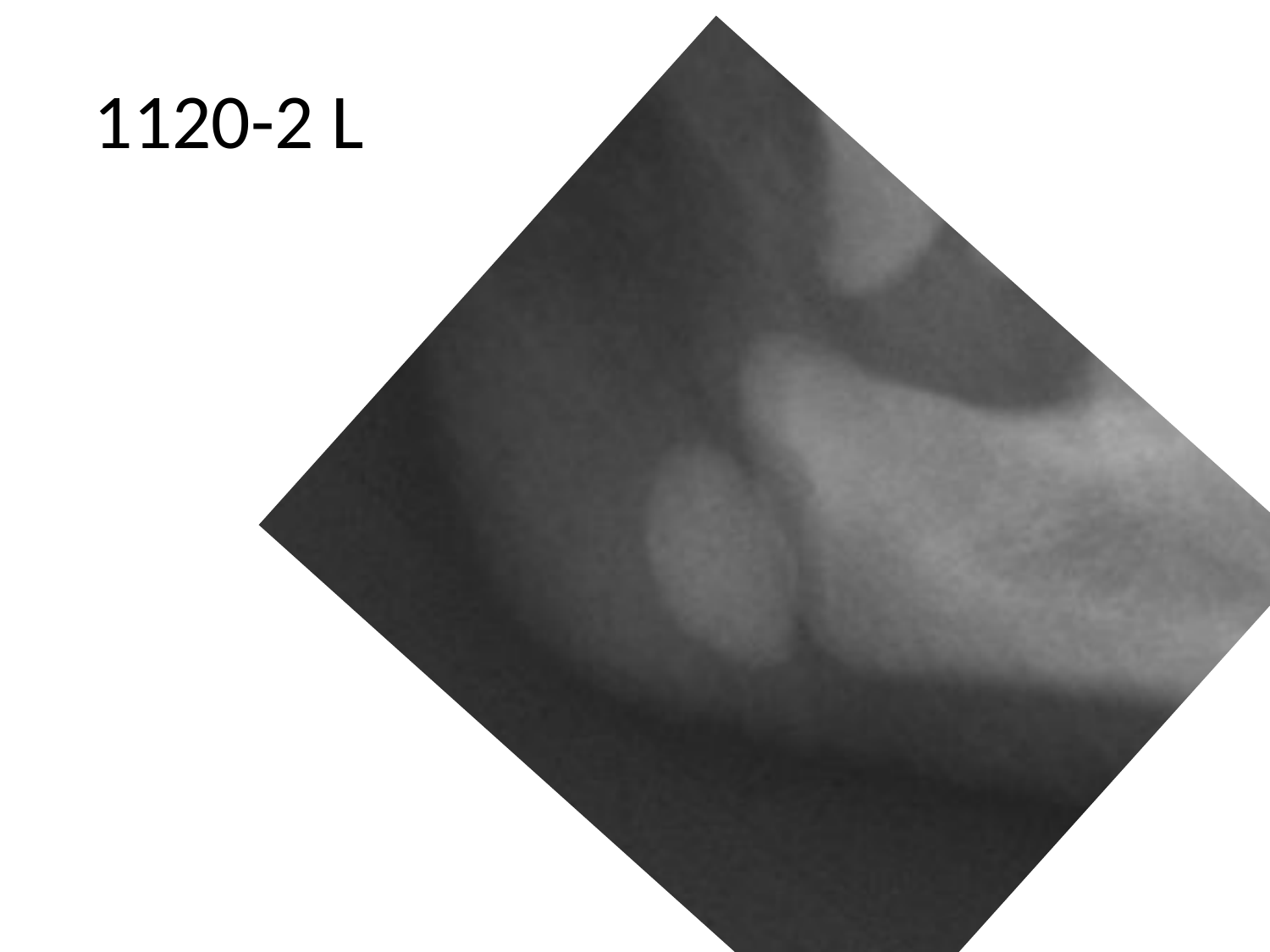

# 1120-2 L

## Slide 29
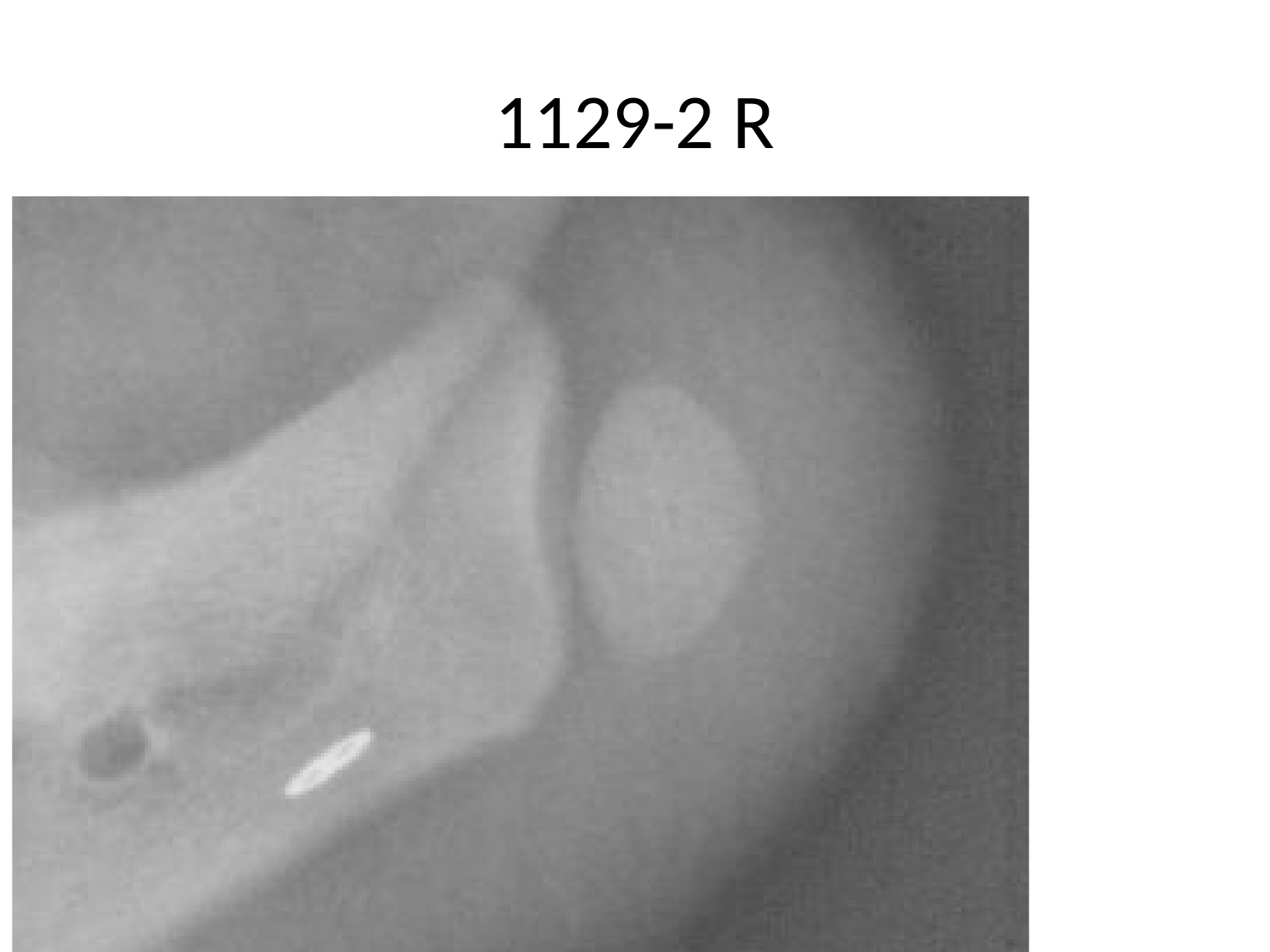

# 1129-2 R

## Slide 30
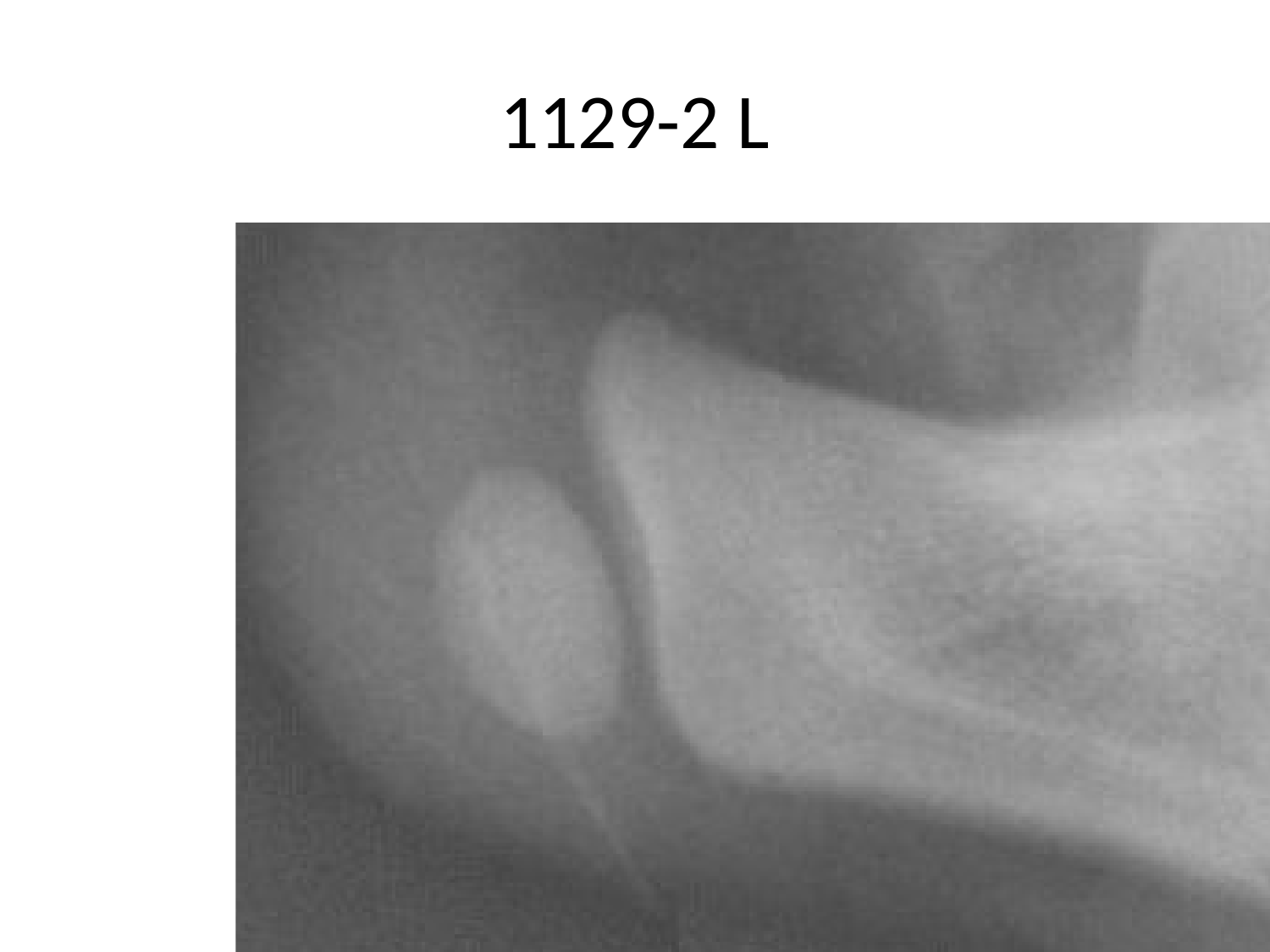

# 1129-2 L

## Slide 31
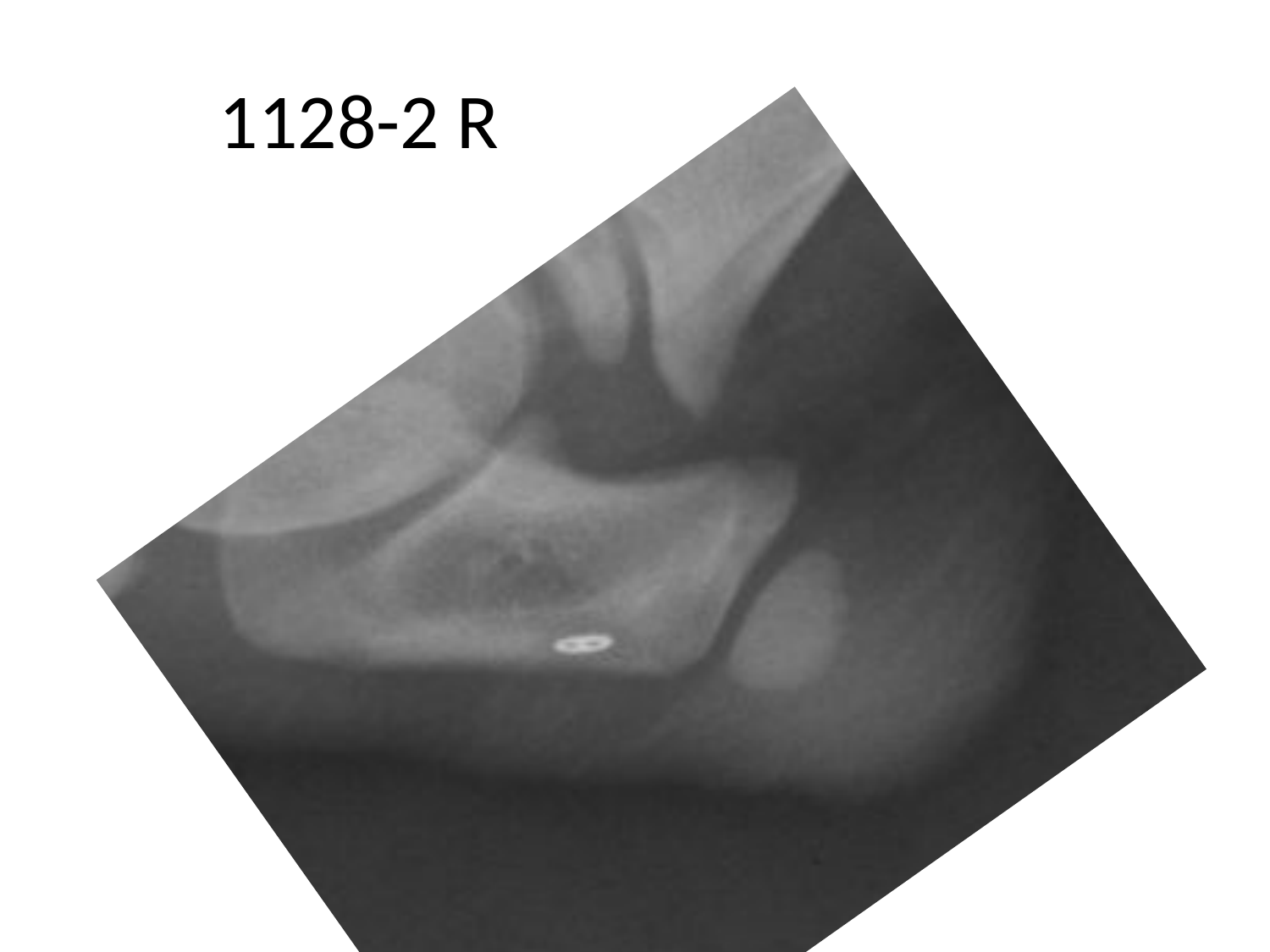

# 1128-2 R

## Slide 32
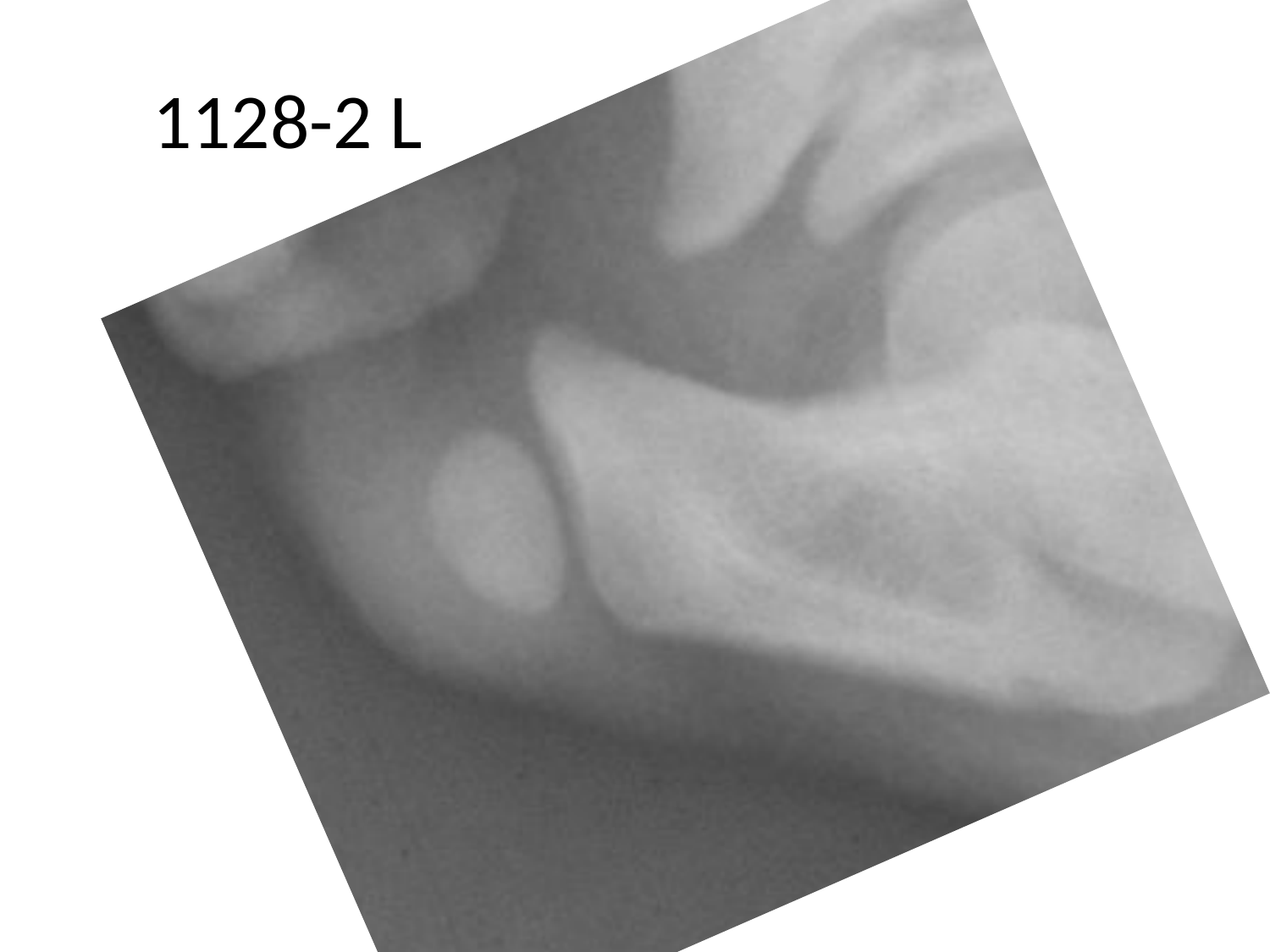

# 1128-2 L

## Slide 33
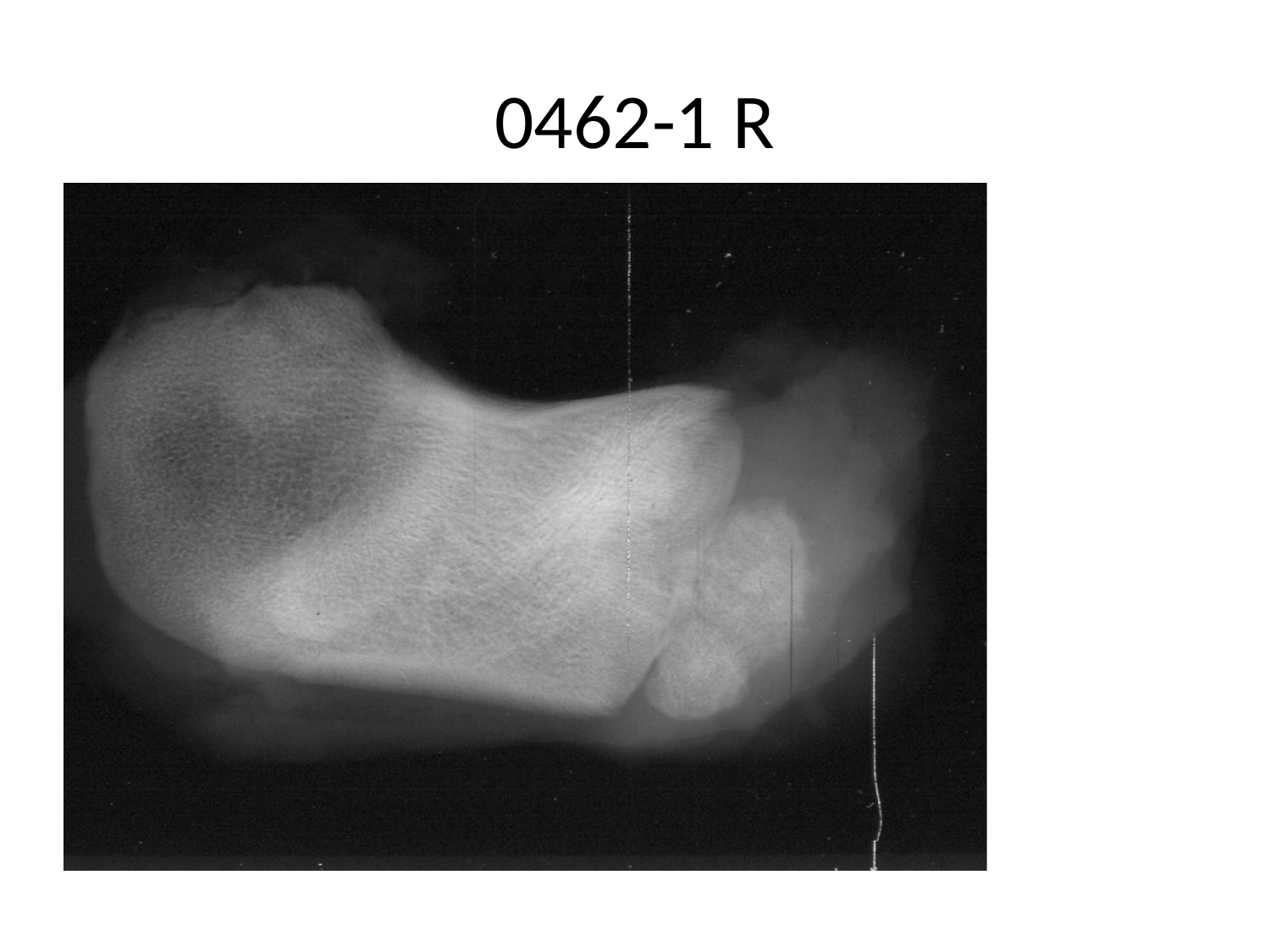

# 0462-1 R

## Slide 34
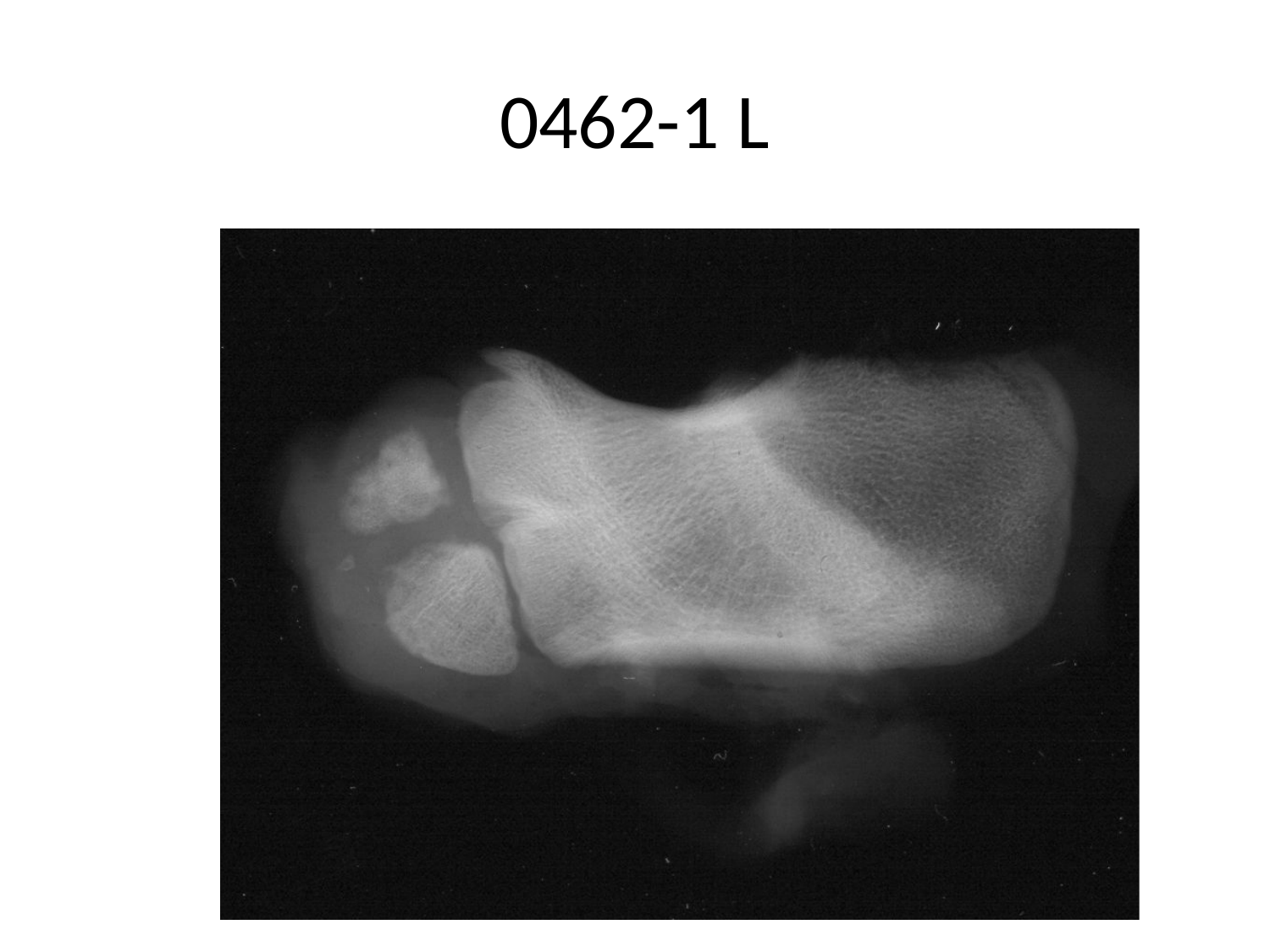

# 0462-1 L

## Slide 35
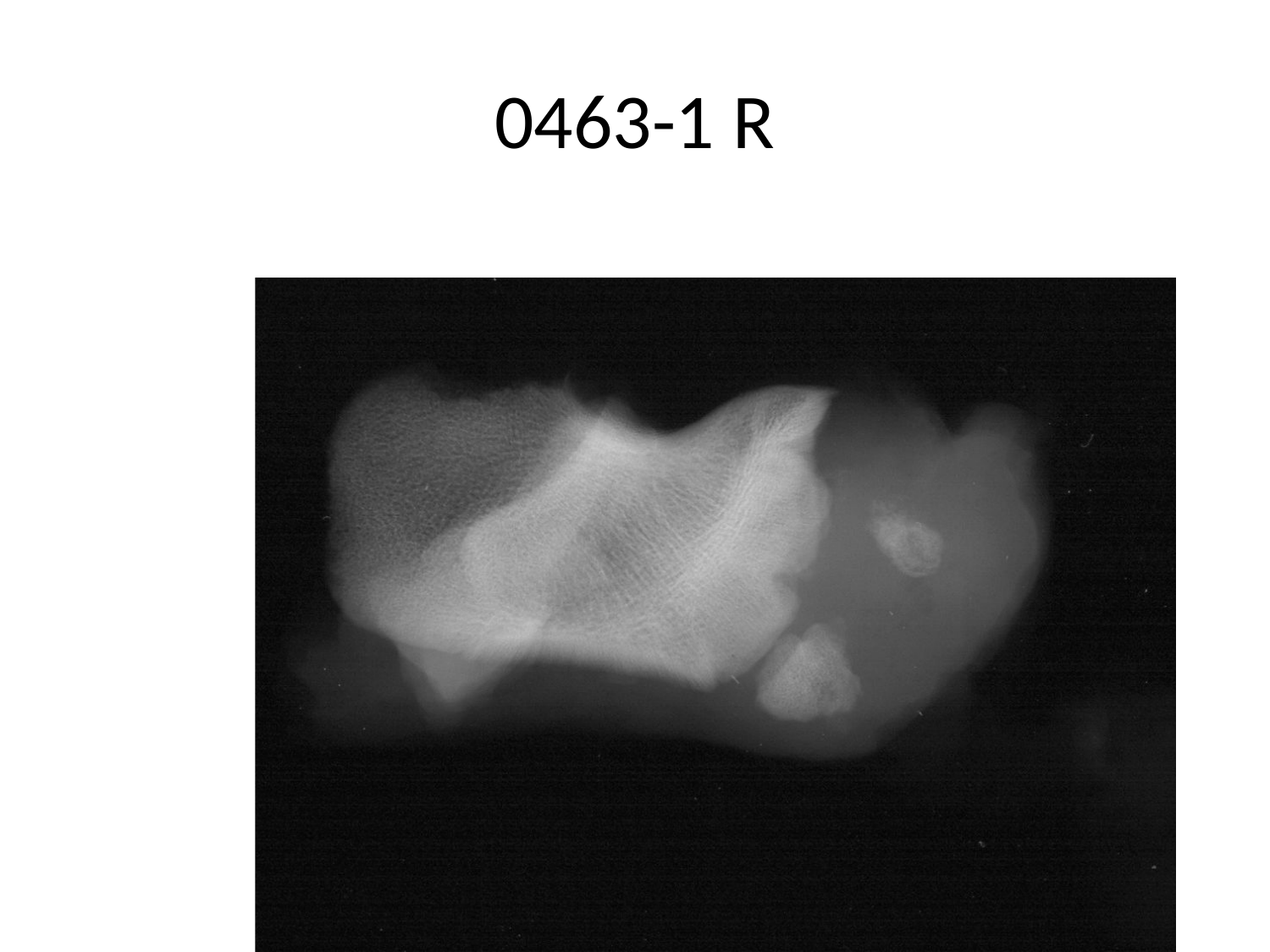

# 0463-1 R

## Slide 36
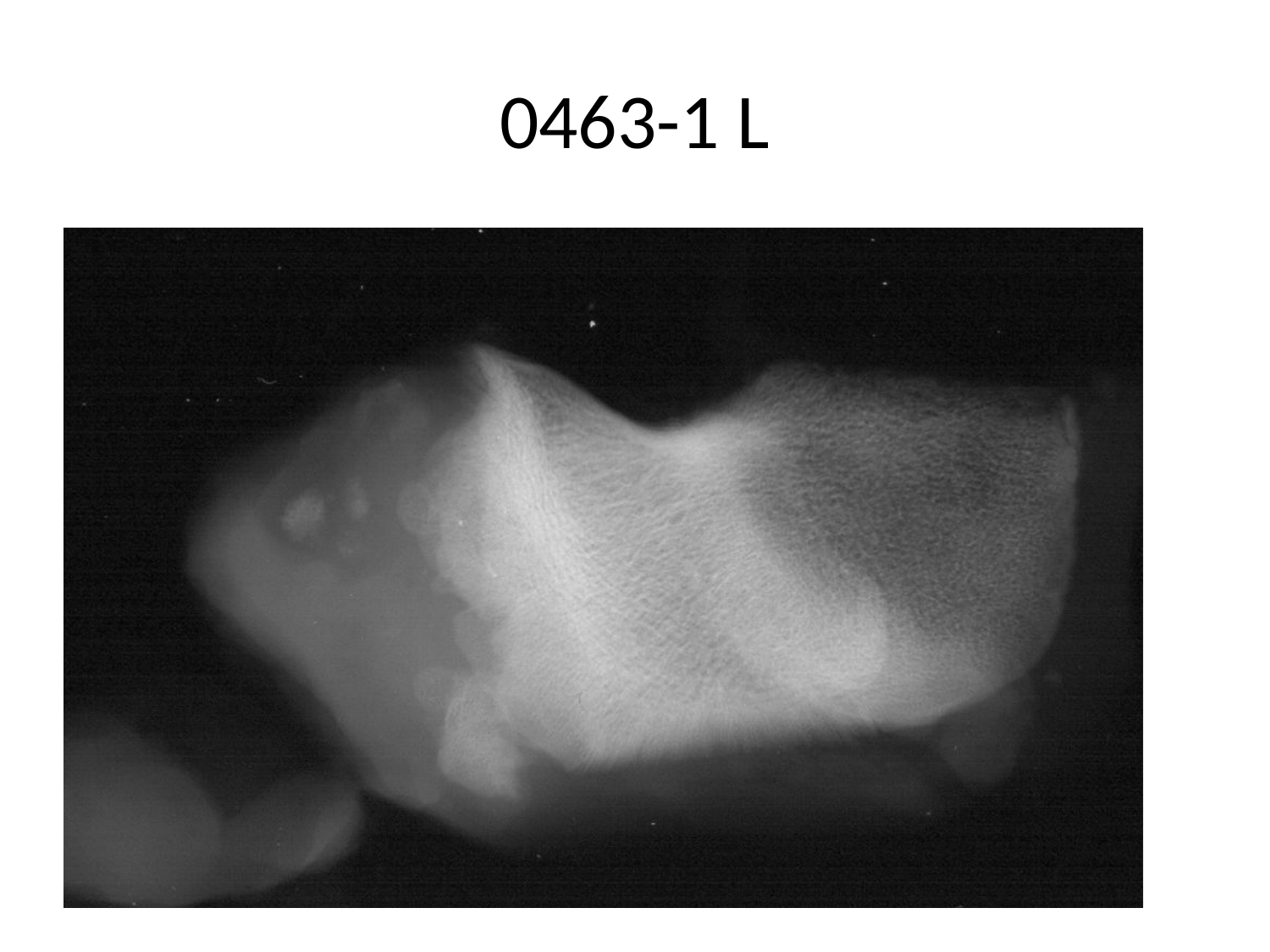

# 0463-1 L

## Slide 37
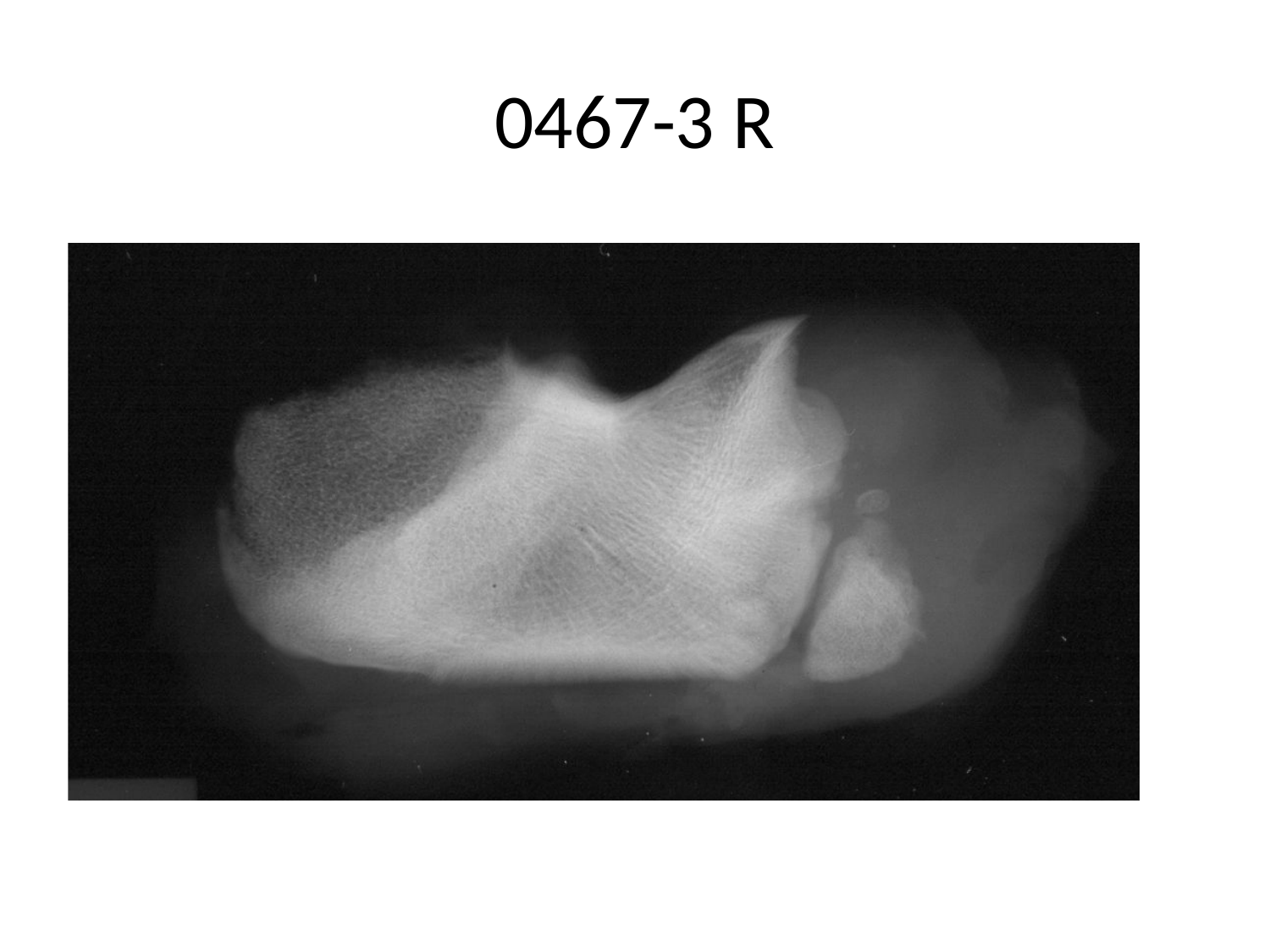

# 0467-3 R

## Slide 38
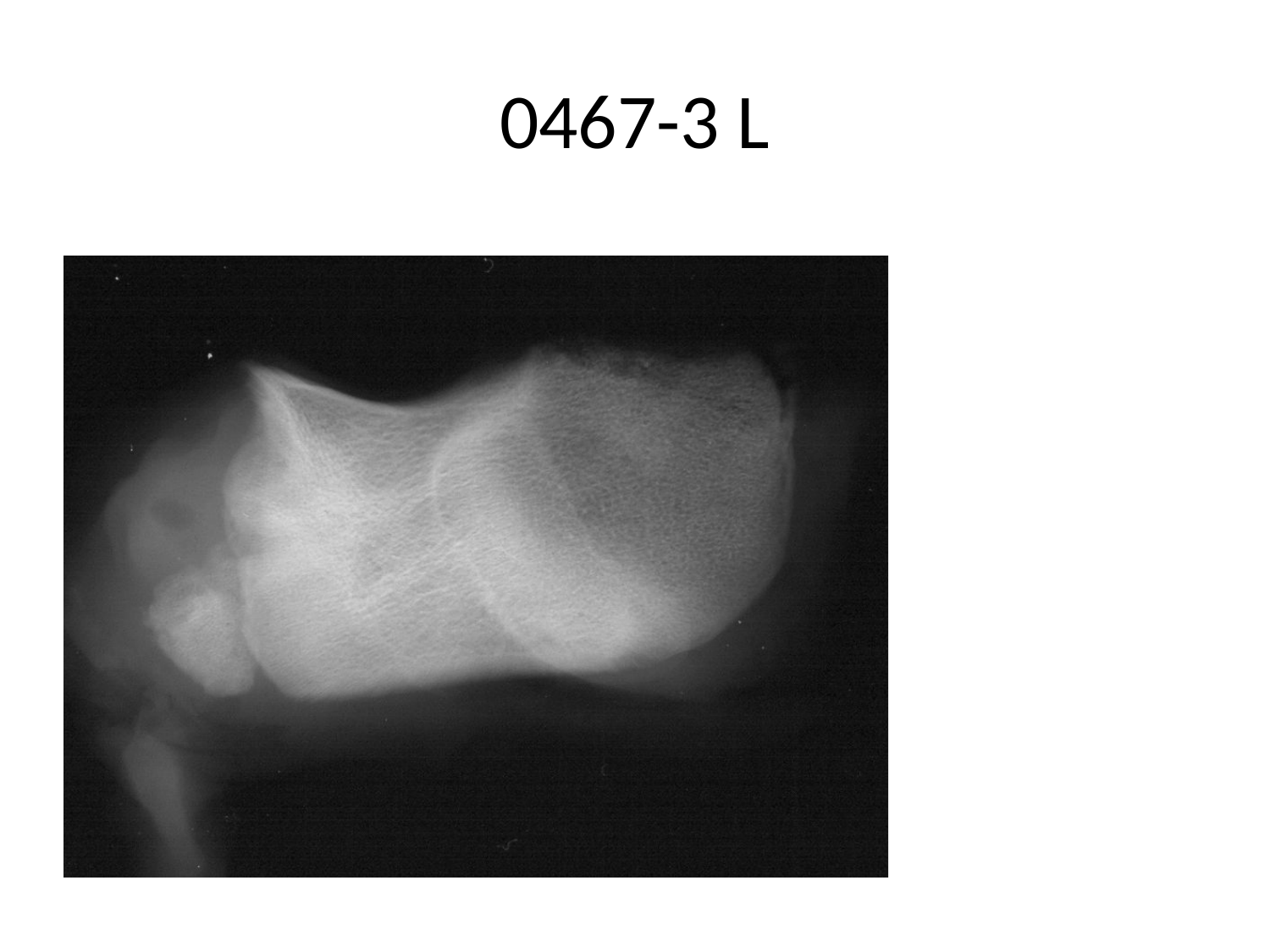

# 0467-3 L

## Slide 39
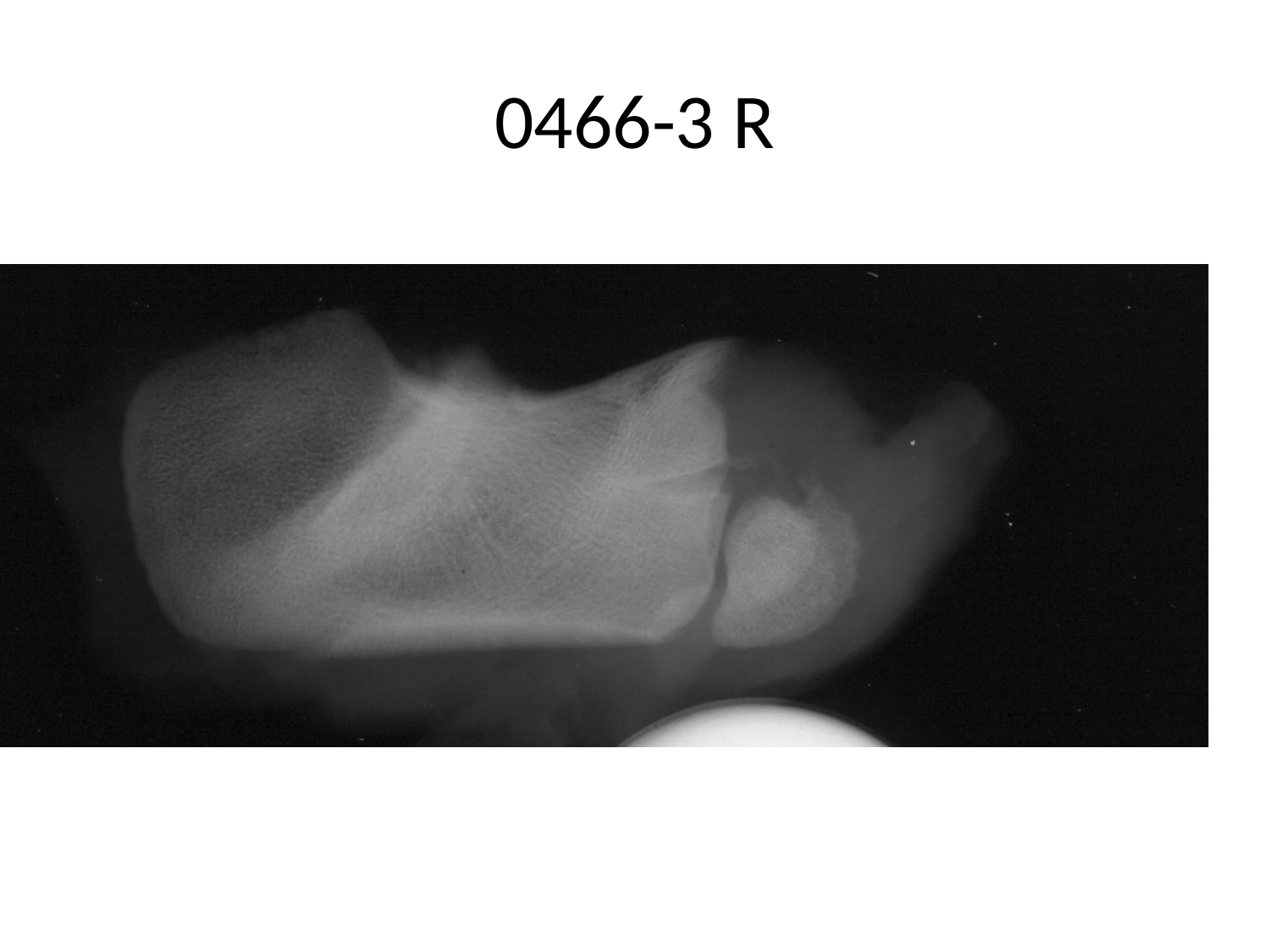

# 0466-3 R

## Slide 40
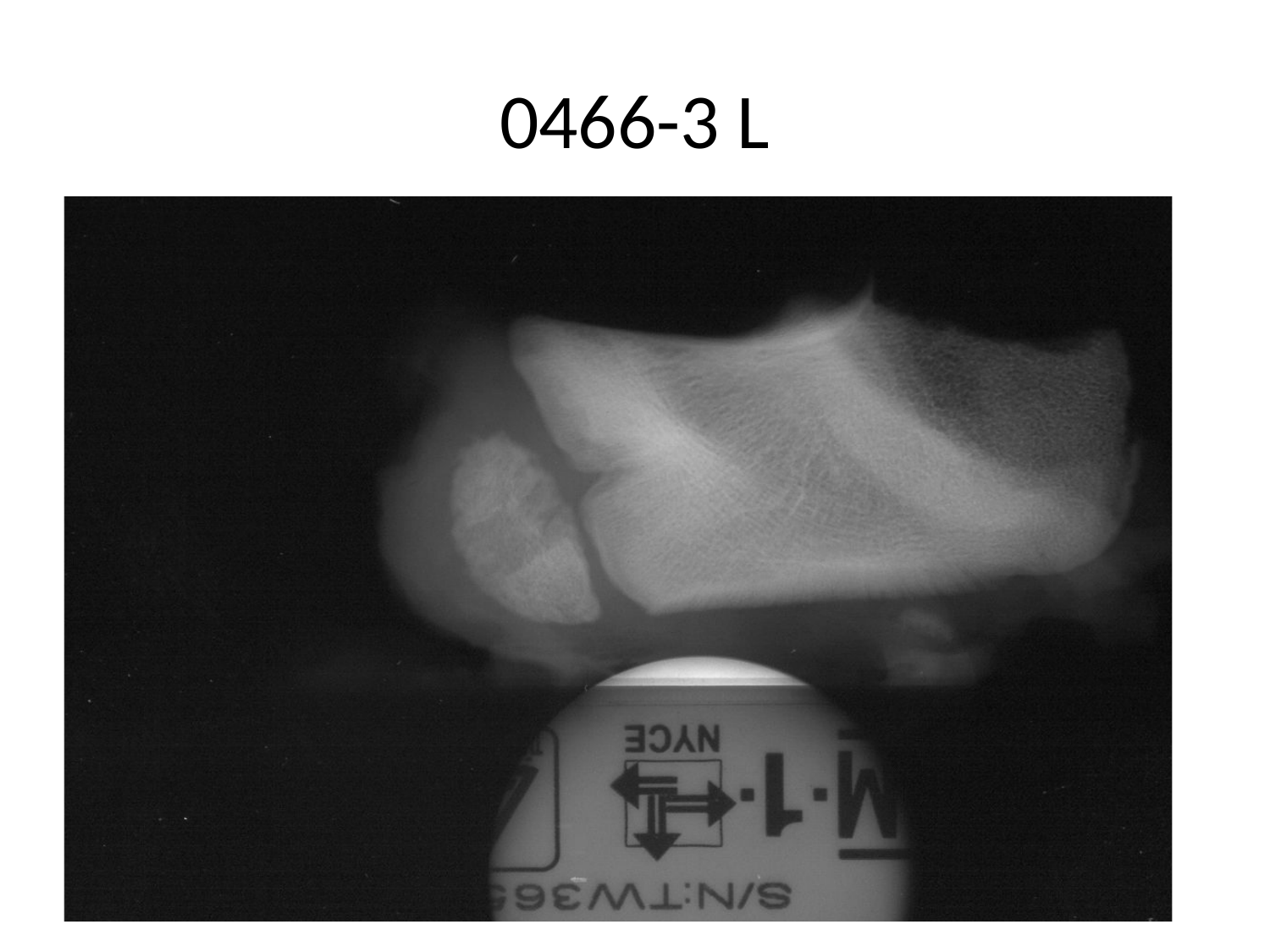

# 0466-3 L

## Slide 41
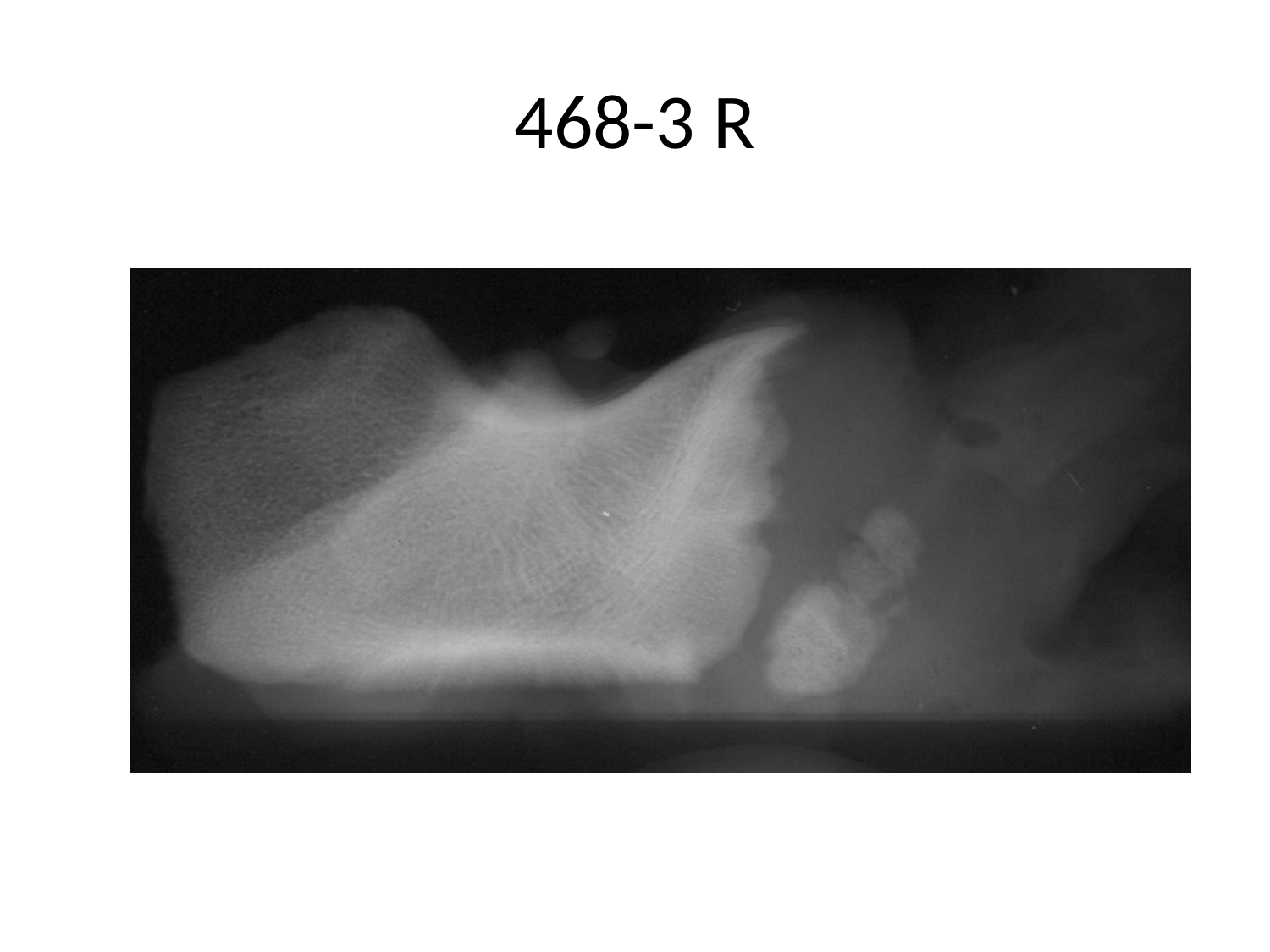

# 468-3 R

## Slide 42
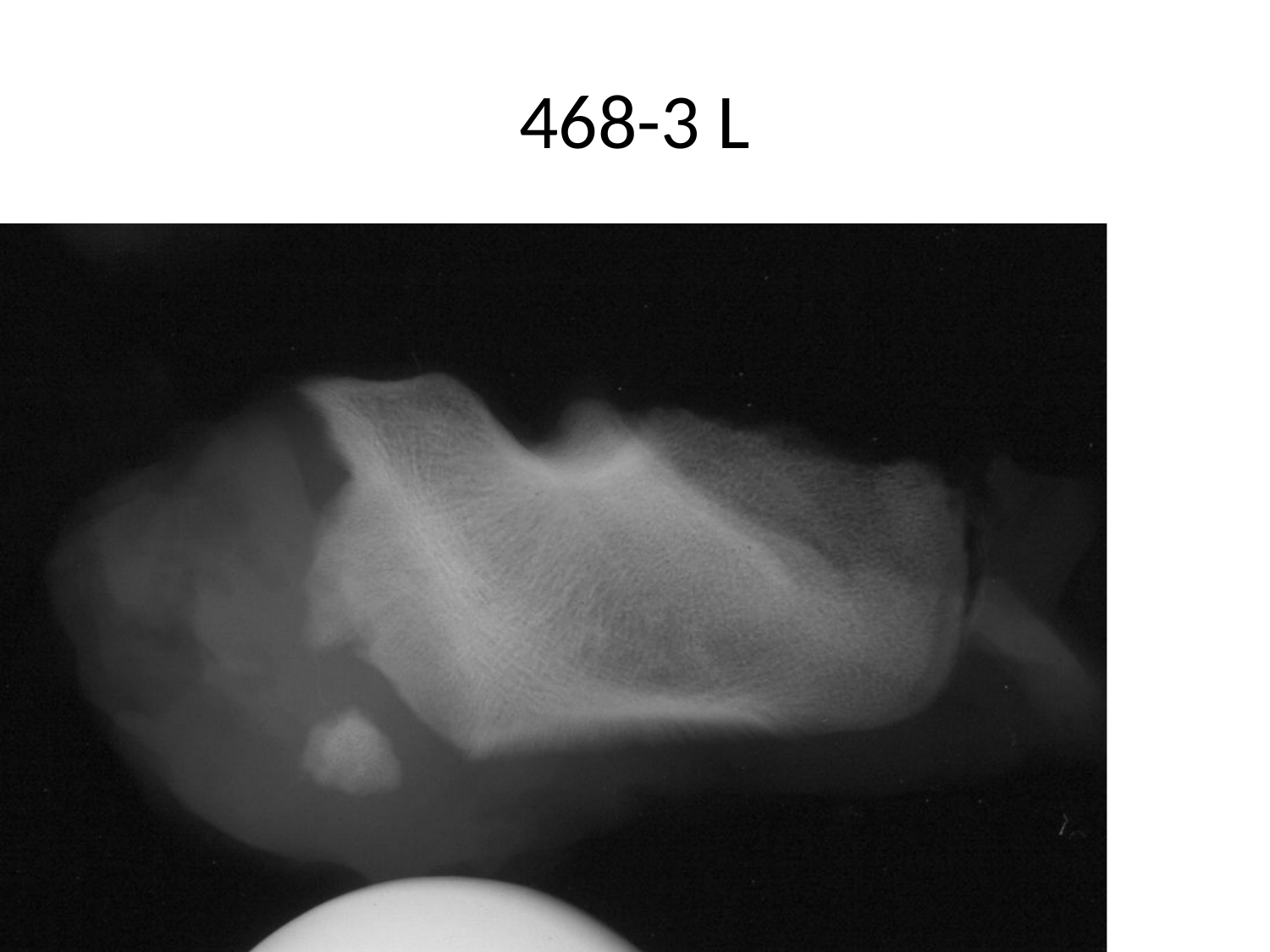

# 468-3 L

## Slide 43
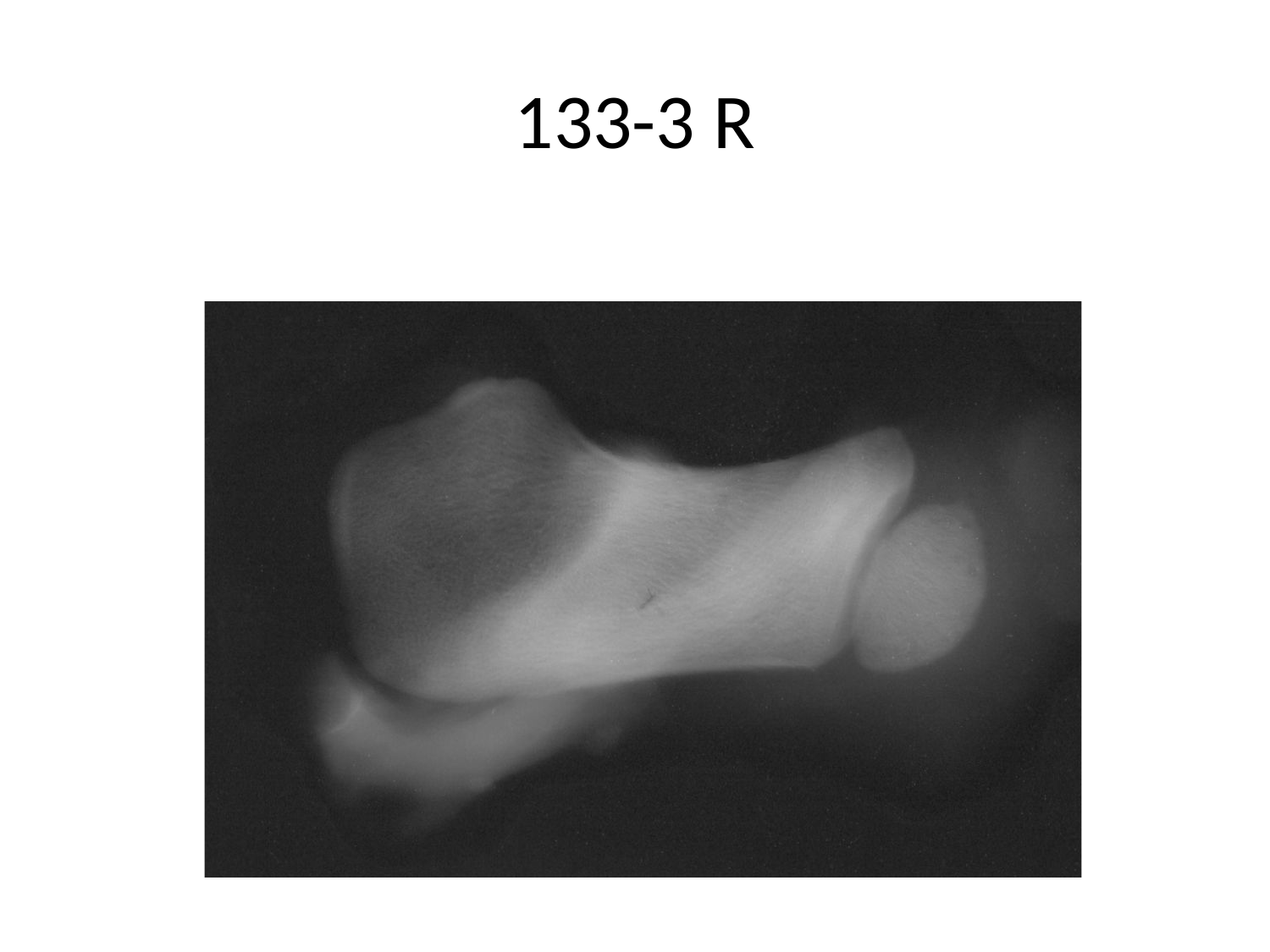

# 133-3 R

## Slide 44
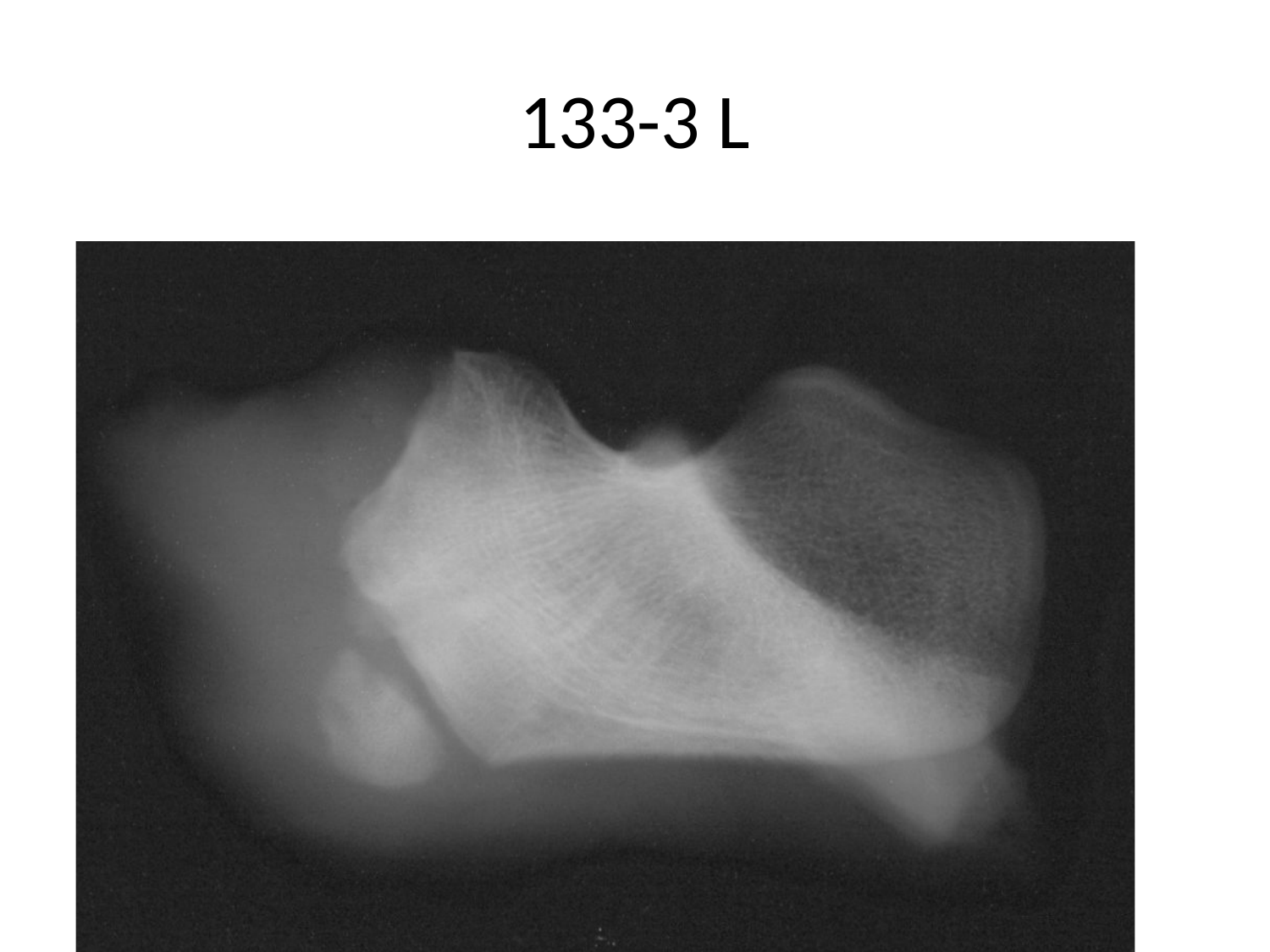

# 133-3 L

## Slide 45
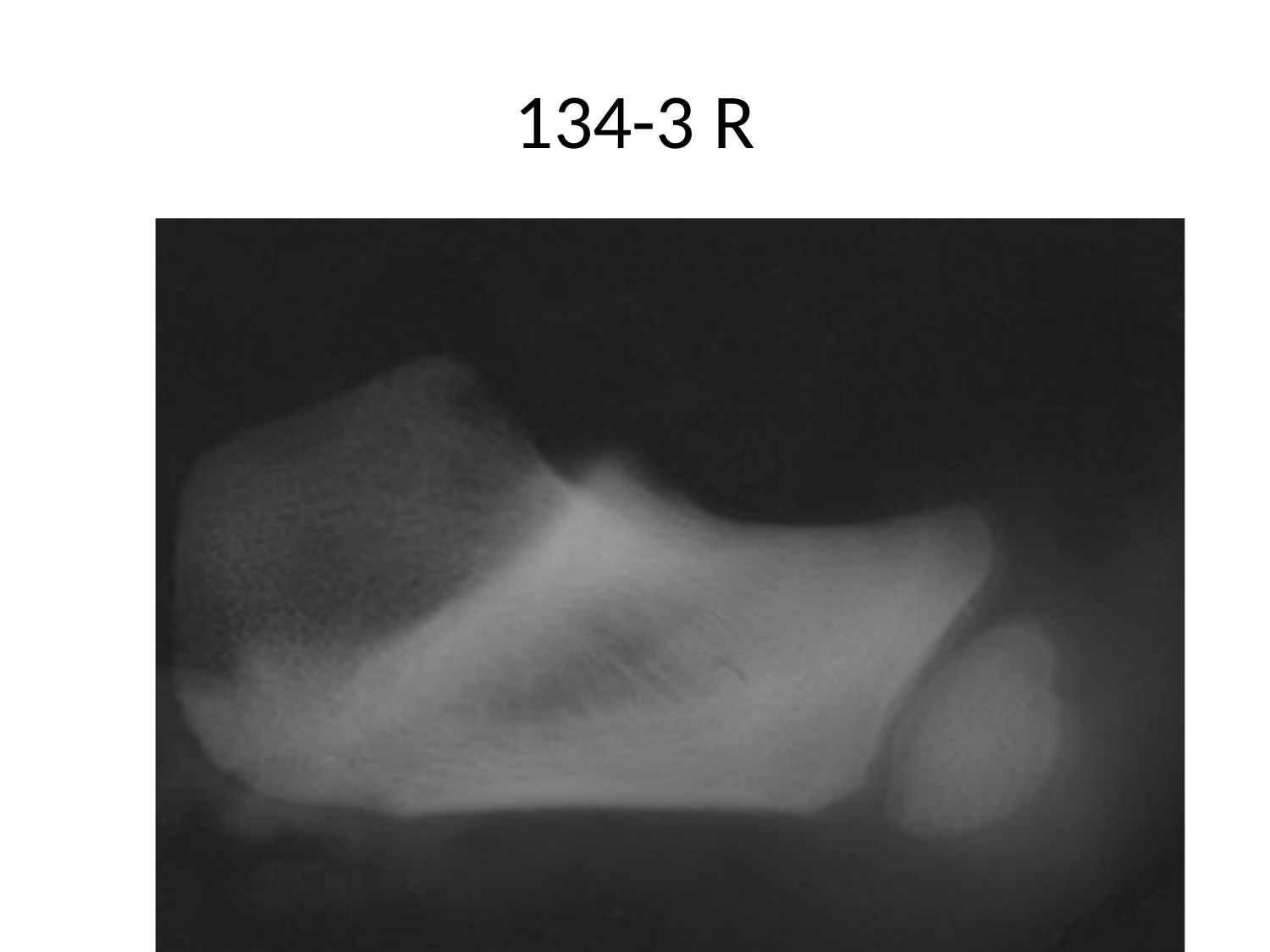

# 134-3 R

## Slide 46
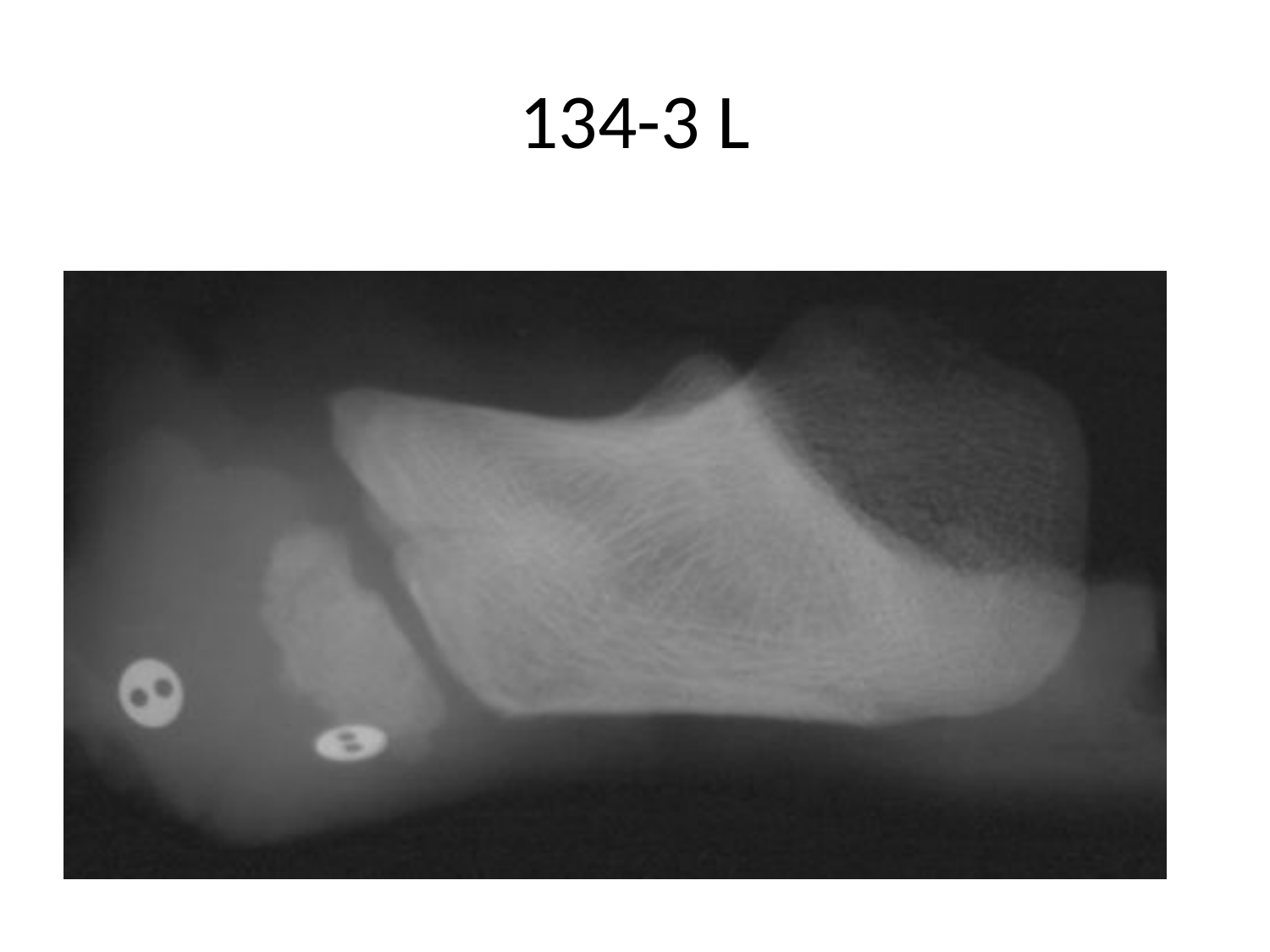

# 134-3 L

## Slide 47
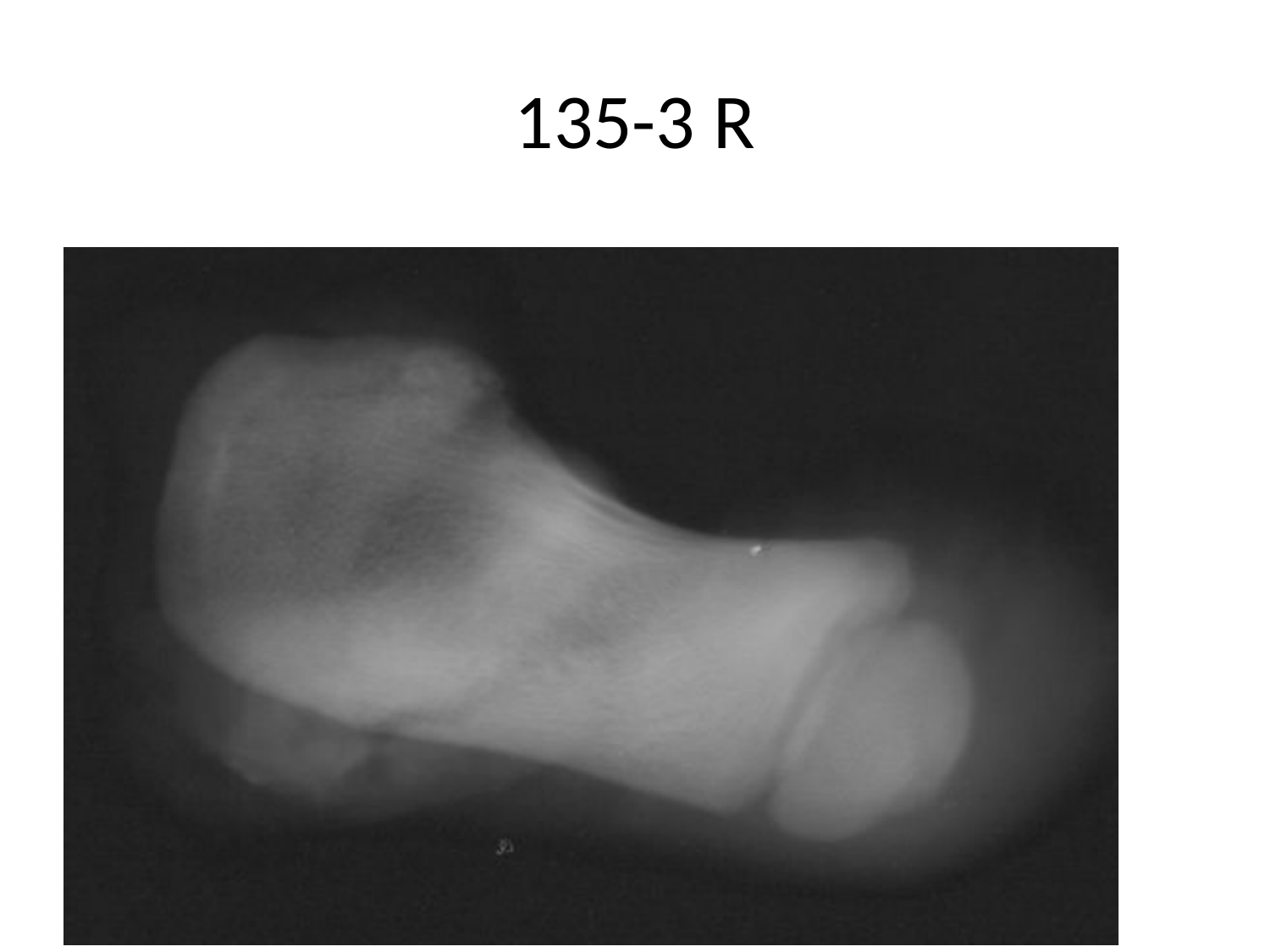

# 135-3 R

## Slide 48
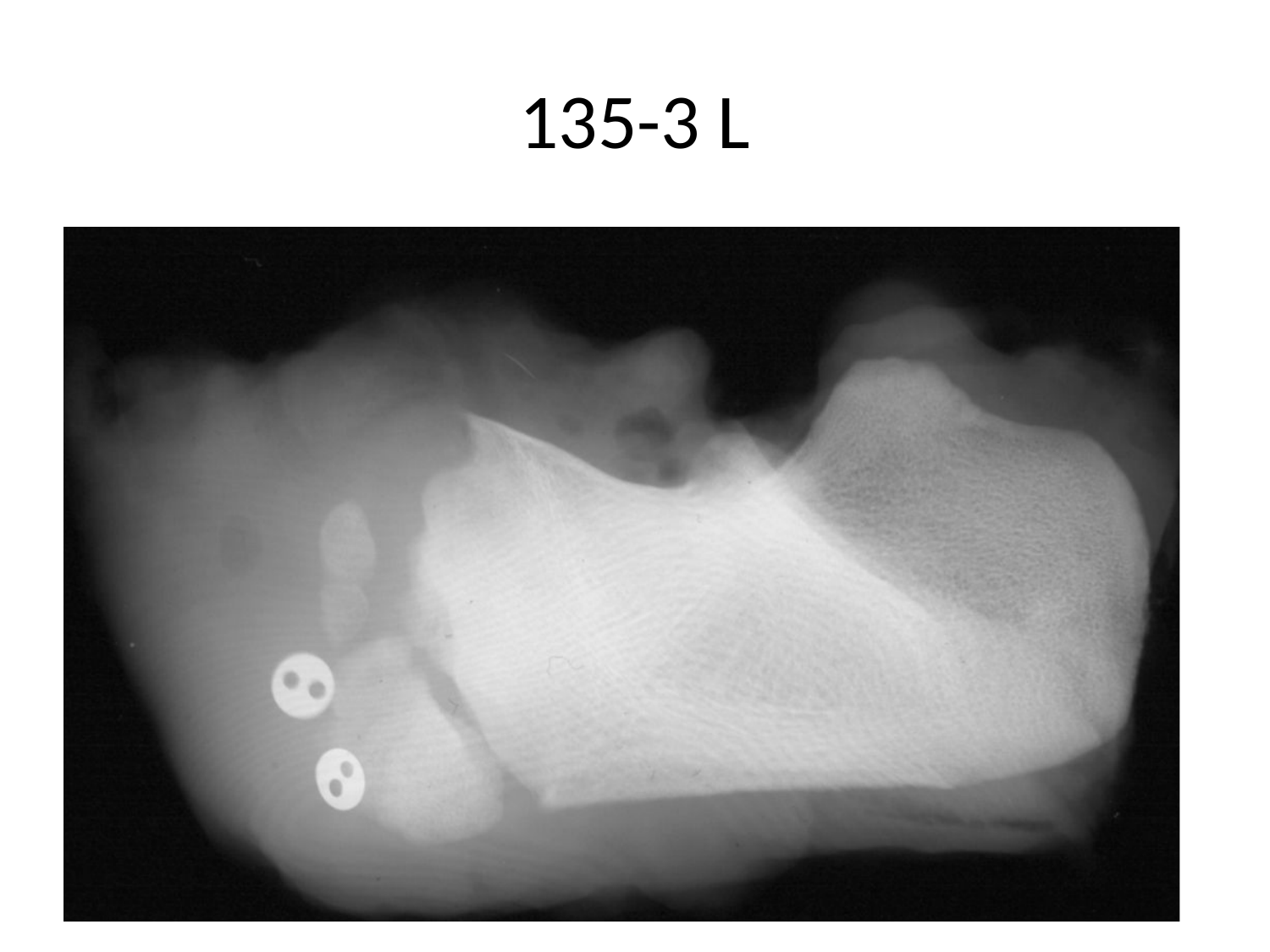

# 135-3 L

## Slide 49
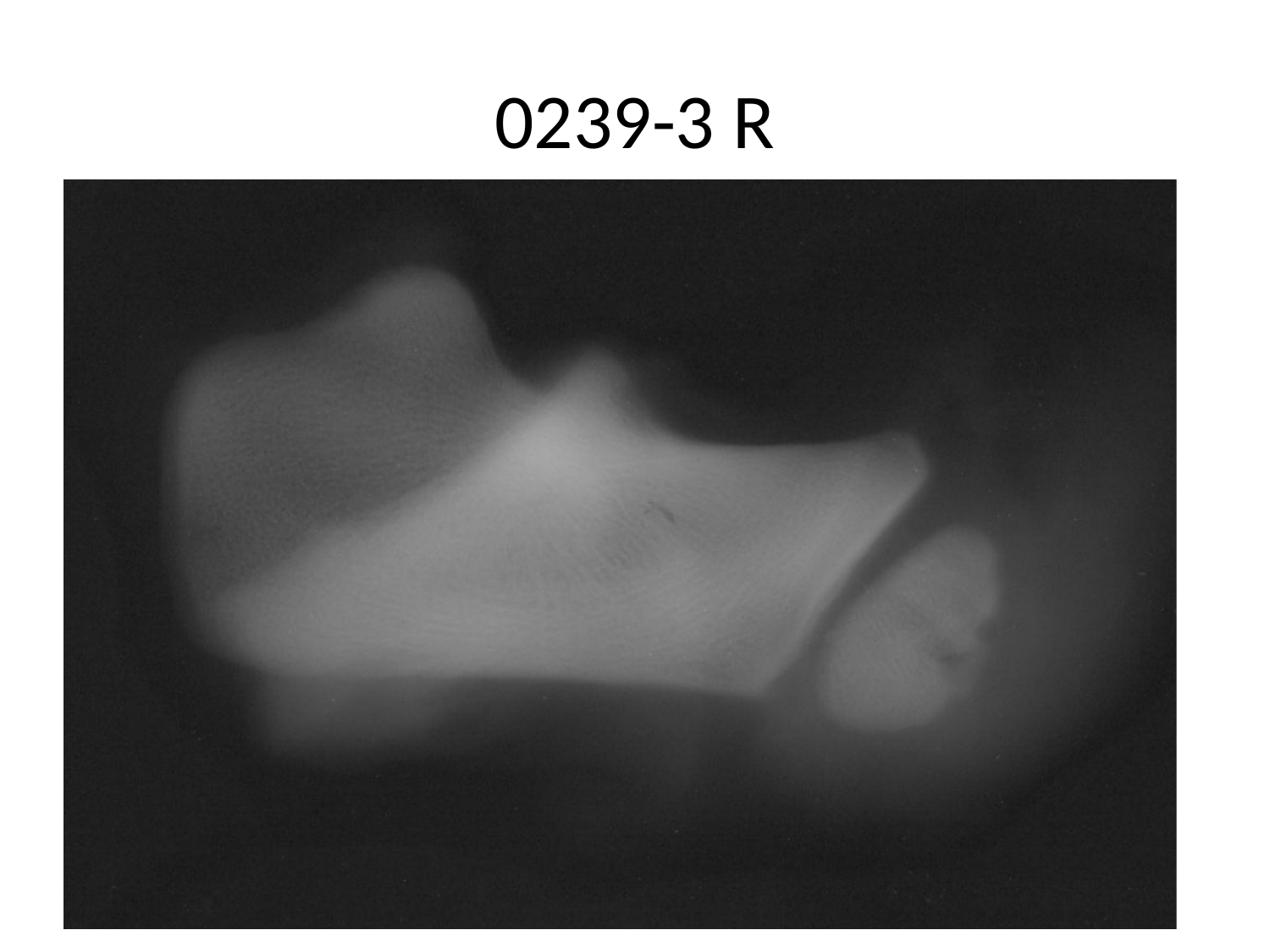

# 0239-3 R

## Slide 50
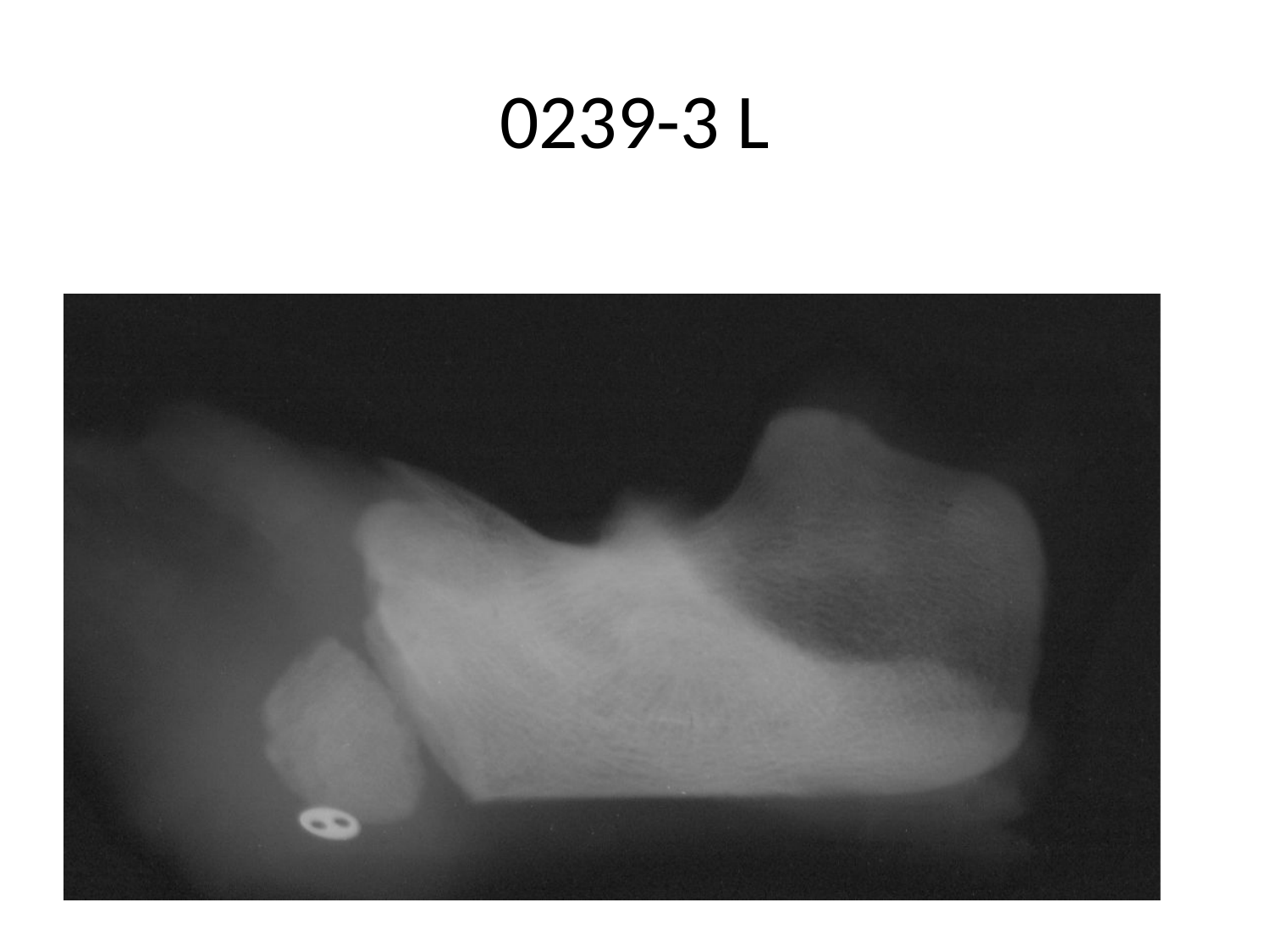

# 0239-3 L

## Slide 51
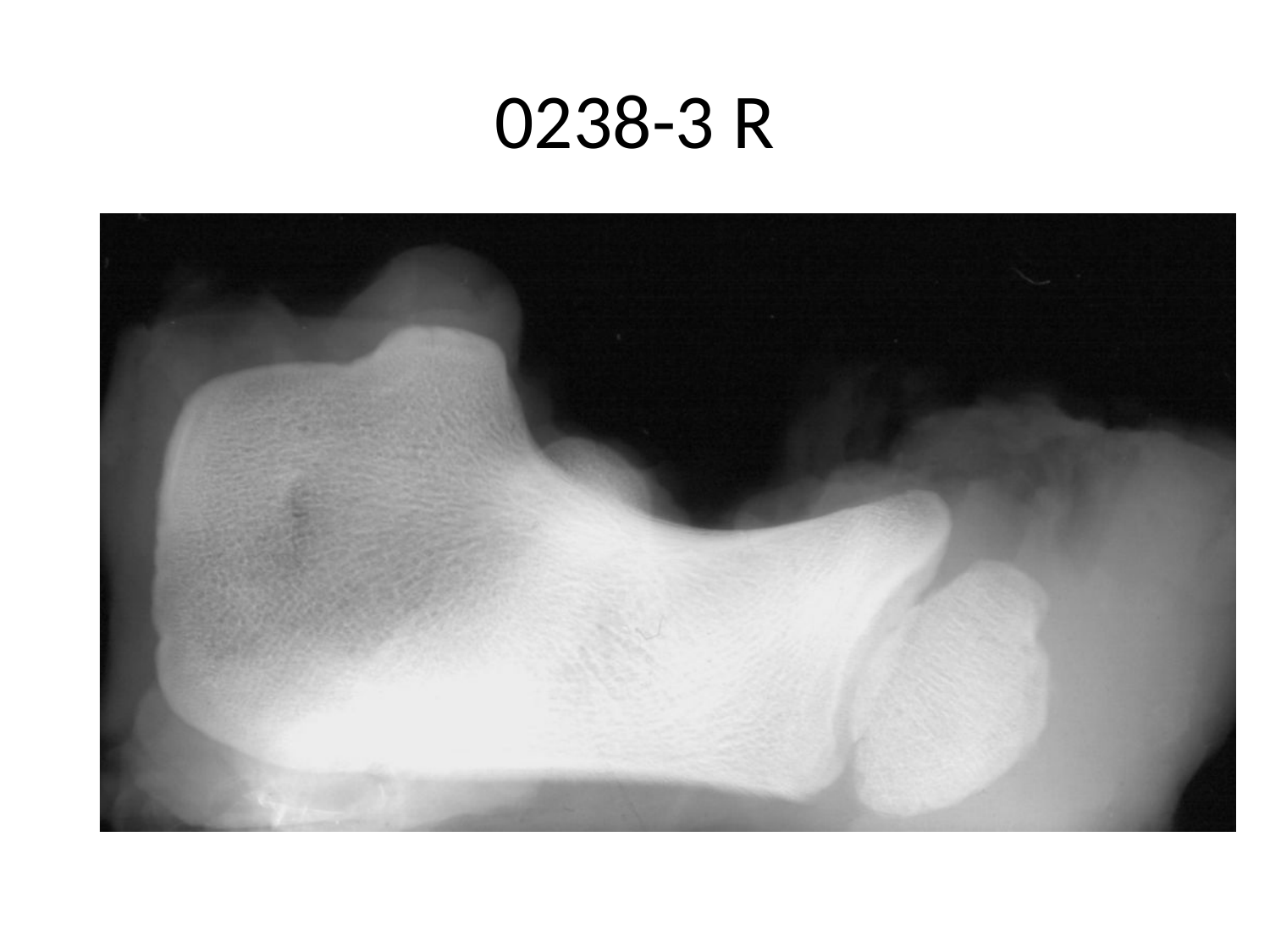

# 0238-3 R

## Slide 52
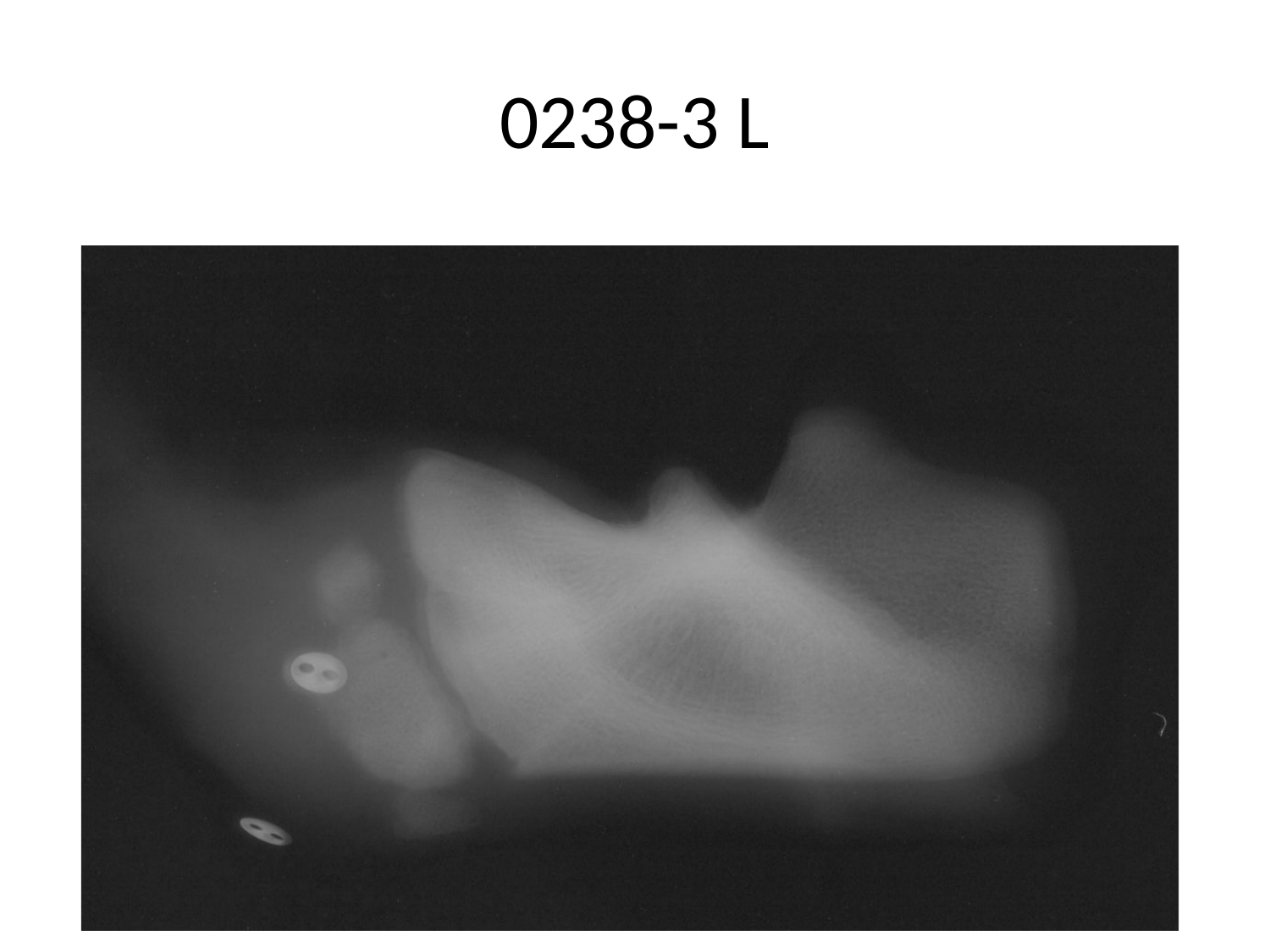

# 0238-3 L
